# Notoginsenoside R1 Ameliorates Cardiac Lipotoxicity Through AMPK Signaling Pathway

**DOI:** 10.3389/fphar.2022.864326

**Published:** 2022-03-17

**Authors:** Xue Tian, Xu Chen, Qianqian Jiang, Qianbin Sun, Tiantian Liu, Yiqin Hong, Yawen Zhang, Yanyan Jiang, Mingyan Shao, Ran Yang, Chun Li, Qiyan Wang, Yong Wang

**Affiliations:** ^1^ School of Life Sciences, Beijing University of Chinese Medicine, Beijing, China; ^2^ School of Traditional Chinese Medicine, Beijing University of Chinese Medicine, Beijing, China; ^3^ Guang’anmen Hospital, Chinese Academy of Traditional Chinese Medicine, Beijing, China; ^4^ Key Laboratory of TCM Syndrome and Formula (Beijing University of Chinese Medicine), Ministry of Education, Beijing, China; ^5^ Beijing Key Laboratory of TCM Syndrome and Formula, Beijing, China; ^6^ Modern Research Center for Traditional Chinese Medicine, Beijing University of Chinese Medicine, Beijing, China

**Keywords:** heart failure, cardiac lipotoxicity, NGR1, AMPK, apoptosis

## Abstract

**Aims**: Cardiac lipotoxicity is the common consequence of lipid metabolism disorders in cardiomyocytes during development of heart failure (HF). Adenosine 5′monophosphate-activated protein kinase (AMPK) acts as an energy sensor and has a beneficial effect in reducing lipotoxicity. Notoginsenoside R1 (NGR1) is extracted from the traditional Chinese medicine Panax notoginseng (Burkill) F.H.Chen (*P. notoginseng*) and has definite cardioprotective effects. However, whether NGR1 can attenuate HF by mitigating lipotoxicity has not been elucidated yet. This study aimed to explore whether NGR1 plays a protective role against HF by ameliorating cardiac lipotoxicity via the AMPK pathway.

**Methods**: In this study, HF mice model was established by left anterior descending (LAD) ligation. palmitic acid (PA) stimulated H9C2 cell model was applied to clarify the effects and potential mechanism of NGR1 on lipotoxicity. *In vivo*, NGR1 (7.14 mg/kg/days) and positive drug (simvastatin: 2.9 mg/kg/days) were orally administered for 14 days. Echocardiography was applied to assess heart functions. Lipid levels were measured by Enzyme-linked immunosorbent assay (ELISA) and key proteins in the AMPK pathway were detected by western blots. *In vitro*, NGR1 (40 μmol/L) or Compound C (an inhibitor of AMPK, 10 μmol/L) was co-cultured with PA stimulation for 24 h in H9C2 cells. CCK-8 assay was used to detect cell viability. Key lipotoxicity-related proteins were detected by western blots and the LipidTOX™ neutral lipid stains were used to assess lipid accumulation. In addition, Apoptosis was assessed by Hoechst/PI staining.

**Results**: NGR1 could significantly improve the cardiac function and myocardial injury in mice with HF and up-regulate the expression of p-AMPK. Impressively, NGR1 inhibited the synthesis of diacylglycerol (DAG) and ceramide and promoted fatty acid oxidation (FAO) *in vivo*. Moreover, NGR1 significantly promoted expression of CPT-1A, the key enzyme in FAO pathway, and down-regulated the expression of GPAT and SPT, which were the key enzymes catalyzing production of DAG and ceramide. *In vitro* experiments showed that NGR1 could significantly attenuate lipid accumulation in PA-induced H9C2 cells and the Hoechst/PI staining results showed that NGR1 ameliorated lipotoxicity-induced apoptosis in PA-stimulated H9C2 cell model. Furthermore, co-treatment with inhibitor of AMPK abrogated the protective effects of NGR1. The regulative effects of NGR1 on lipid metabolism were also reversed by AMPK inhibitor.

**Conclusion**: NGR1 could significantly improve the heart function of mice with HF and reduce cardiac lipotoxicity. The cardio-protective effects of NGR1 are mediated by the activation of AMPK pathway.

## 1 Introduction

Heart failure (HF) after acute myocardial infarction (MI) is the major cause of death globally (Jinawong et al., 2021; Writing et al., 2021). Percutaneous coronary intervention (PCI) is an effective method to treat MI and can significantly improve the survival rate of patients (Ozaki et al., 2018). However, reperfusion aggravates the damage of the ischemic heart and the risk of developing HF remains high (Bulluck et al., 2016; Ottani et al., 2018). Therefore, it is important to develop new strategies to prevent myocardial cell death and thus limit myocardial damage in patients with MI.

As an organ with high oxygen consumption, high energy consumption and high metabolic rate, the heart needs a large amount of energy to maintain its normal physiological function and metabolic requirements. Under normal conditions, adult cardiomyocytes derive approximately 50–70% of their energy from FAO (Lopaschuk and Jaswal 2010). Under hypoxic conditions, myocardial cells undergo metabolic disturbances due to acute ischemia. FAO is reduced and there is an accumulation of toxic lipid intermediates such as ceramide and diacylglycerol (DAG). This imbalance in lipid metabolism caused by accumulation of ceramide and DAG is commonly referred as cardiac lipotoxicity (D'Souza et al., 2016). Numerous clinical data have shown that myocardial ceramide levels increase dramatically 24 h after MI (Hadas et al., 2020) and ventricular remodeling in patients with HF is closely related to the accumulation of toxic lipids (Chokshi et al., 2012; Ji et al., 2017). However, the pathogenesis and potential treatment of lipotoxicity remain to be further studied.

AMPK, as a “cellular fuel gauge,” plays a critical role in regulating intracellular glycolipid and energy metabolism (Hardie 2015). Evidence suggest that inactivation AMPK plays an important role in the development of lipotoxicity and AMPK may be a key target in the prevention and treatment of lipotoxicity in HF (Sakamoto et al., 2017). Expressions of key enzymes in lipid metabolism are regulated by AMPK. Serine-palmitoyl transferase (SPT) is a heterodimer composed of two subunits, SPT long chain 1 (SPTLC1) and SPT long chain 2 (SPTLC2), and is the rate-limiting enzyme for *de novo* ceramide synthesis. Glycerol-3-phosphate acyltransferases (GPAT) is the rate-limiting enzyme in the lipid anabolic pathway and is involved in the *de novo* synthesis of DAG (Kojta et al., 2020). Carnitine palmitoyltransferase-1A (CPT-1A) is located in the inner membrane of cell mitochondria and is a key rate-limiting enzyme in the FAO. It has been shown that AMPK can effectively regulate SPT, GPAT, and CPT-1A. DAG and ceramide are second messengers in lipid metabolism and are often associated with impaired cardiac function, which in turn leads to mitochondrial dysfunction (Engin 2017). Moreover, recent studies shown that ceramide and DAG can decrease the expression of the anti-apoptotic protein B cell lymphoma-2 (Bcl-2), which in turn promote apoptosis (Korshunova et al., 2021; Sramek et al., 2021). Up to now, it is not clear whether myocardial ischemic-induced cardiac damage can be improved by reducing lipotoxicity via AMPK signaling pathway.

*Panax notoginseng* (Burk.) F. H. Chen (*P. notoginseng*) is a traditional Chinese herbal medicine, which is widely used in the prevention and treatment of cardiovascular diseases (Wang et al., 2016). Clinical and *in vivo* studies showed that *P. notoginseng* has the pharmacological effects of activating blood and removing stasis, and reducing inflammation and analgesia (Han et al., 2013; Yang et al., 2016; Luo et al., 2017; Xu et al., 2018). *Panax notoginseng* saponin (PNS) are the main active ingredients in *P. notoginseng*, and NGR1 is one of the major components of PNS (Liu J. et al., 2014; Xu et al., 2019). Our previous study confirmed that PNS could improve cardiac function and reduce myocardial injury in HF rats (Chen et al., 2021). NGR1 has been reported to exert cardiac protective effects by its anti-inflammatory and anti-fibrotic properties (Yu et al., 2016; Xiao et al., 2018). Meanwhile, NGR1 is one of the main ingredients of Xuesaitong injection, which has been widely used in the treatment of cardiovascular diseases in clinical practice (Pang et al., 2017; Yang et al., 2017). However, the effect of NGR1 on cardiac lipotoxicity and the regulatory mechanism has not been reported yet.

In this study, the effects of NGR1 on cardiac injury were investigated by *in vivo* and *in vitro* experiments. The regulatory effects of NGR1 on AMPK signal pathway and its effect on lipotoxicity were also intensively explored. This research will provide a new insight into the roles of lipotoxicity in HF and novel strategies of managing HF.

## 2 Materials and Methods

### 2.1 Reagents and Chemicals

NGR1 (purity ≥98%, molecular weight = 933.14) was purchased from Aladdin Biochemical Technology Co., Ltd. (Shanghai, China). Simvastatin was purchased from Merck Sharp & Dohme Limited (U.K.). Triglyceride (TG), non-esterified fatty acids (NEFA) and 2′,7′-dichlorodihydrofluorescein diacetate (DCFH-DA) probe were purchased from Nanjing Jiancheng Institute of Biotechnology (Nanjing, China). CK-MB kits were obtained from Beijing Beijian Xinchuangyuan Biotechnology Co., Ltd. (Beijing, China). Cardiac troponin I (cTnI) was purchased from Wuhan Huamei Biotech Co., Ltd. (Wuhan, China). Ceramide were purchased from Ruixin Biotech Co., Ltd. (Fujian, China) and DAG were purchsaed from Mlbio Biotech Co., Ltd. (Shanghai, China). Dulbecco’s modified eagle medium (DMEM), fetal bovine serum (FBS), streptomycin and penicillin were obtained from Corning (New York, United States). Bovine serum albumin (BSA), palmitic acid (PA), LipidTOX™ neutral lipid stains, 4′,6′-Diamidino-2-phenylindole (DAPI) were purchased from Sigma (St. Louis, MO, United States). Hoechst/PI assay was obtained from Beijing Solarbio Technology Co., Ltd. (Beijing, China). The CCK-8 assay (Dojindo, Kumamoto, Japan) was obtained from Dojindo Laboratories (Kumamoto, Japan). RIPA lysis buffer, protein phosphatase inhibitor and bicinchoninic acid (BCA) assay were obtained from Beijing Pulilai Gene Technology (Beijing, China).

### 2.2 Animal Model and Groupings

Forty male-ICR mice (28 ± 2 g) in SPF grade were purchased from Beijing Sibeifu Laboratory Animal Technology Co., Ltd. (Beijing, China). The model of HF was induced by coronary artery left anterior descending (LAD) artery as previously described **(** Zhang et al., 2019). Briefly, the mice were weighed and anaesthetized with pentobarbital sodium (0.5%, 0.08 ml/10 g) by intraperitoneal injection, followed by tracheal intubation and ventilation with an HX-101 E constant-volume rodent ventilator (Chengdu Techman Software Co., Ltd., China). The chest cavity of mice were exposed to accomplish the ligation of LAD with 7–0 surgical line. The sham group only threaded but not ligated. Mice were randomly divided into four groups, including sham group, model group, NGR1 group (7.14 mg/kg/days) and positive drugs group (simvastatin, 2.9 mg/kg/days) after the LAD operation. In this study, the doses administered for NGR1 were converted based on rat dose equivalence (He et al., 2014), and the doses administered for the positive drug simvastatin were converted based on clinical treatment dose equivalence. All mice gavagly administered for 14 days.

### 2.3 Echocardiography

Two-dimensional echocardiography (Vevo TM 2100; Visual Sonics, Toronto, ON, Canada) was used to evaluate the cardiac function. After mice were anesthetized by isoflurane, the ejection fraction (EF), fractional shortening (FS), left ventricular internal diameter at end-diastole (LVID; d) and left ventricular internal diameter at end-systole (LVID; s) of mice were detected and at least three cardiac cycles consecutively were captured.

### 2.4 Histological Assay

Heart tissues were fixed in 4% paraformaldehyde, embedded in paraffin, and cut into 5 mm thick sections. The paraffin sections were stained with hematoxylin-eosin (H&E) and Masson simultaneously, then they were scanned under an optical microscope (Leica Microsystems GmbH).

### 2.5 Biochemical Analysis

The blood was extracted from the abdominal aorta under anesthesia and centrifuged at 3,000 r/min for 20 min. Serum triglyceride (TG), cholesterol (CHOL), high density lipoprotein-cholesterol (HDL-C), low density lipoprotein-cholesterol (LDL-C), non-esterified fatty acids (NEFA) and creatine kinase MB (CK-MB) were detected using automatic biochemical analyzer (HITACHI 7080, Japan). Cardiac troponin I (cTnI) was detected using Enzyme-linked immunosorbent assay (ELISA).

### 2.6 Detection of Diacylglycerol, Ceramide and Non-Esterified Fatty Acids

The clipped myocardial tissue was added to a glass homogenizer with the corresponding volume of phosphate-buffered saline (PBS) (generally at a weight-to-volume ratio of 1:9) and ground thoroughly on ice. The mixture was centrifuged at 2000 r/min for 20 min and the supernatant was carefully collected. After processing the samples, ceramide, DAG and NEFA were performed in strict accordance with the kit instructions.

### 2.7 Cell Culture

H9C2 cells were purchased from China Infrastructure of Cell Line Resources (Chinese Academy of Medical Sciences, China). The cells were cultured in dulbecco’s modified eagle medium (DMEM) with 10% fetal bovine serum (FBS) and 100 mg/ml streptomycin and 100 U/mL penicillin at 37°C in a humidified incubator (Thermo, NYC, United States).

### 2.8 Establishment of Cardiac Lipotoxicity Model

The PA solution was prepared by saponifying PA with sodium hydroxide (NAOH) and mixing it with fatty acid-free bovine serum albumin (BSA) (Yang et al., 2019). As follows, PA was added to NAOH solution and placed in thermostat water bath for 30 min at 70°C. Next, the saponification solution was mixed with 1% fatty acid-free BSA to form a 1 mM stock solution, which was diluted with DMEM containing 5% FBS for use.

PA was used to stimulate H9C2 cells, which is the most commonly used method to induce lipotoxicity *in vitro* (Moscoso et al., 2019; Chang et al., 2021; Li et al., 2021). When H9C2 cell density reached 70–80%, the medium was removed and they were stimulated with 50–800 μmol/L PA for 24 h. In subsequent experiments, H9C2 cells were divided into four groups: normal group, PA (100 μmol/L) group, NGR1 (40 μmol/L) group and NGR1+Compound C (10 μmol/L) group.

### 2.9 Cell Viability Assay

The CCK-8 assay was used to determine cell viability. Briefly, after different treatments, the original medium was discarded and CCK-8 solution was added to each well and incubated for 2 h at 37°C in the dark. Then cell viability was measured at 450 nm using a microplate reader (Thermo, NYC, United States).

### 2.10 LipidTOX™ Neutral Lipid Staining

After the cells were treated, cells were fixed with 4% paraformaldehyde for 15 min at room temperature. Then, the LipidTOX™ neutral lipid stains was performed at a dilution ratio of 1:1,000 for at least 30 min, followed by 3 times washes in PBS, then nuclei were stained with 4′,6′- Diamidino-2-phenylindole (DAPI) for 15 min and photographed after 3 times washes in PBS. Images were collected using a laser confocal microscopy (Leica Microsystems GmbH) and analyzed with ImageJ software (National Institutes of Health).

### 2.11 Detection of Non-Esterified Fatty Acids and Triglyceride in Cells

Cells were collected, adequately lysed and fragmented after treatment. NEFA and TG assay were performed in strict accordance with the manufacturer’s instructions.

### 2.12 Apoptosis Measurement

Hoechst/PI staining was used to assess cell apoptosis. After treatment, cells were co-stained with Hoechst 33,342 and PI for 20 min at 4°C in the dark and subsequently photographed by a laser confocal microscopy. ImageJ software was used to analyze fluorescent images.

### 2.13 Reactive Oxygen Species (ROS) Measurement

According to the manufacturer’s instructions, the intracellular ROS levels were measured by the ROS assay. After different stimuli, cells were incubated with 2′,7′-dichlorodihydrofluorescein diacetate (DCFH-DA) probe for 30 min at 37°C in the dark, and finally washed 3 times with PBS and photographed. The fluorescence images of intracellular ROS obtained by confocal microscopyImages were acquired by a laser confocal microscopy and analyzed with ImageJ software.

### 2.14 Western Blots

Total protein was obtained from myocardial tissue and H9C2 cells using pre-cold RIPA lysis buffer and 1% protein phosphatase inhibitor and protein concentration was measured by bicinchoninic acid (BCA) assay. Subsequently, protein samples were separated by sodium dodecyl sulfate polyacrylamide gel electrophoresis (SDS-PAGE) and transferred to polyvinylidene fluoride (PVDF) membranes. After that, PVDF membranes were blocked in 5% milk, incubated with primary antibodies at 4°C overnight, incubated with secondary antibody for 1 h at room temperature and exposed to the ECL in gel imager. Image-Lab software was used to analyze data. The antibodies were used as follows: rabbit anti-AMPKα (5,831, Cell Signaling Technology, Germany), rabbit anti-AMPK-phosphor-T172 (CY5608, Abways, China), rabbit anti-SPTLC1 (DF12752, Affinity, China), rabbit anti-SPTLC2 (DF12231, Affinity, China), mouse anti-GPAT (sc-398135, Santa cruz, China), rabbit anti-CPT-1A (DF12004, Affinity, China), rabbit anti-phosphor-AKT (CY6569, Abways, China), rabbit anti-AKT (CY5551, Abways, China), mouse anti-Bcl-2 (AB3359, Abways, China), rabbit anti-Bax (00089105, proteinch, China), rabbit anti-GAPDH (AB0037, Abways, China).

### 2.15 Statistical Analysis

All data were presented as the mean ± standard deviation (SD) and all statistical analyses were performed using Graphpad Prism 8 software. The data were determined by One-way analysis of variance (ANOVA) and *t*-test. Significance was accepted at *p* < 0.05.

## 3 Results

### 3.1 NGR1 Improved Cardiac Functions and Protected Myocardial Structure in the Heart Failure Mice Model

**FIGURE 1:**
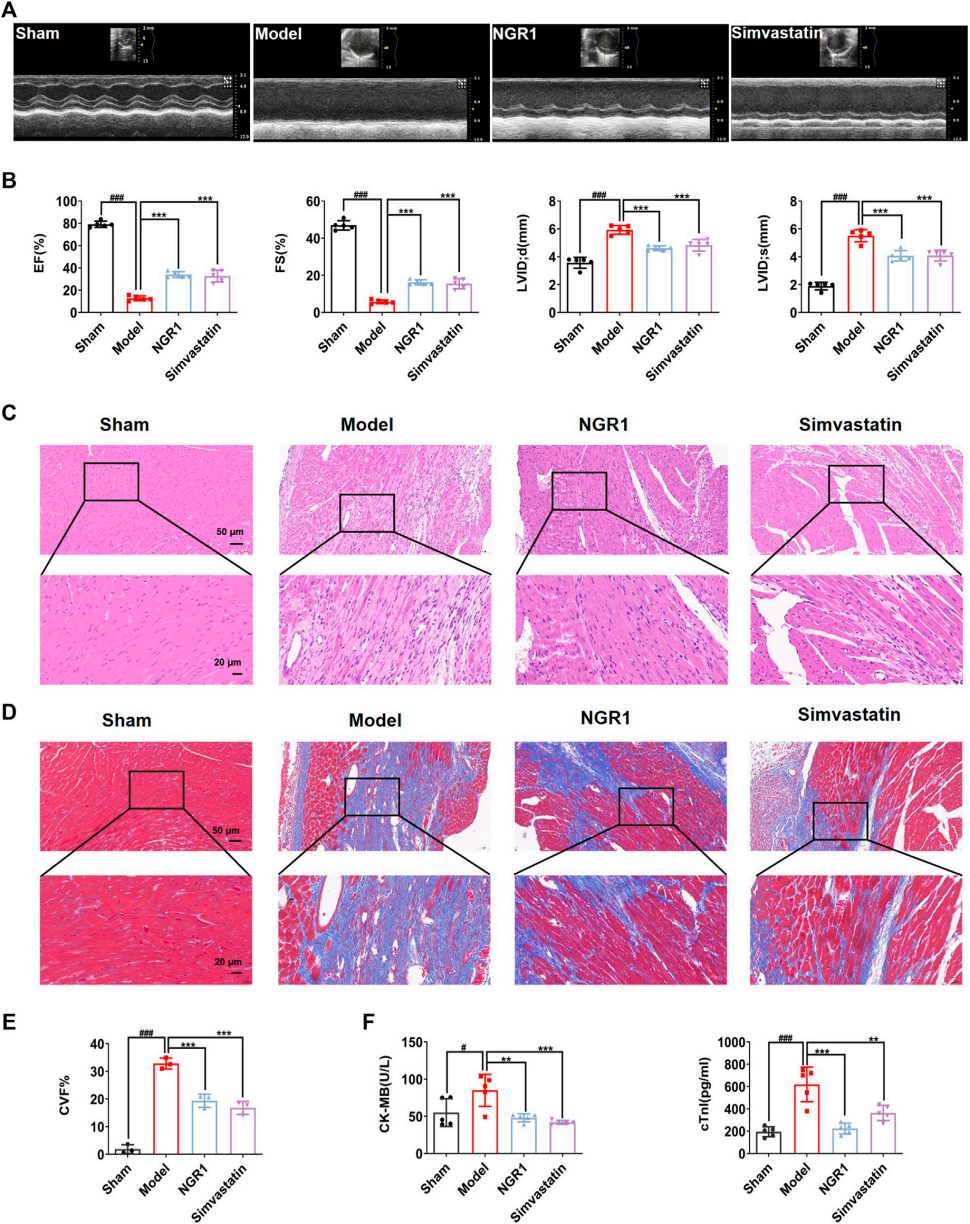
NGR1 treatment enhanced cardiac function and alleviated myocardial pathological changes in the HF mice model. **(A)** Representative images of 2M-mode echocardiography in the sham, model, NGR1 and simvastatin group. **(B)** Quantitative analysis of the ejection fraction (EF), fractional shortening (FS), left ventricular internal diameter at end-diastolic (LVID;d) and left ventricular internal diameter at end-systolic (LVID;s). *N* = 5 per group **(C)** Representative pictures of H&E staining of each group. Scale bar = 20 μm/50 μm, *N* = 3 per group. **(D)** Representative pictures of Masson staining of each group. Scale bar = 20 μm/50 μm, *N* = 3 per group **(E)** Semi-quantitative results of collagen volume fraction (CVF), the formula was CVF = collagen fiber area/myocardial surface area × 100%. **(F)** Heart failure biomarkers including CK-MB and cTnI in serum were detected. **p < 0.01, ***p < 0.001 vs. model group, ^#^ *p* < 0.05, ^###^ *p* < 0.001 vs. sham group, *N* = 5 per group.

After 14 days of treatment, echocardiography indicated that the HF model was successfully established, as evidenced by a significant decrease in EF and FS in the mice of the model group as compared with the sham group (*p* < 0.001) (Figures 1A,B). After NGR1 intervention, the EF and FS values were up-regulated (*p* < 0.001) and the LVID; d and LVID; s values were down-regulated (*p* < 0.001) (Figures 1A,B). H&E staining showed that the myocardial cells in the sham group were well-arranged, with intact nuclei and no inflammatory cell infiltration. However, rupture and disorder of myocardial fibers and edema of myocardial tissue were observed in the model group. The pathological changes were reversed by NGR1 treatment (Figure 1C). Results of masson staining indicated that the degree of fibrosis was increased in the model group, which could be attenuated after NGR1 treatment (Figures 1D,E). CK-MB and cTnI are known as specific markers of myocardial injury (Xie et al., 2019). Our results showed that compared with the sham group, the levels of serum CK-MB and cTnI were elevated in the model group (*p* < 0.05, *p* < 0.001), while NGR1 treatment reversed these changes (*p* < 0.01, *p* < 0.001) (Figure 1F). These results suggested that NGR1 treatment exerted a cardio-protective effect in the HF mice model. Simvatatin had similar effects as NGR1 (Figures 1A,F).

### 3.2 NGR1 Regulated Lipid Metabolism in the HF Mice Model

**FIGURE 2:**
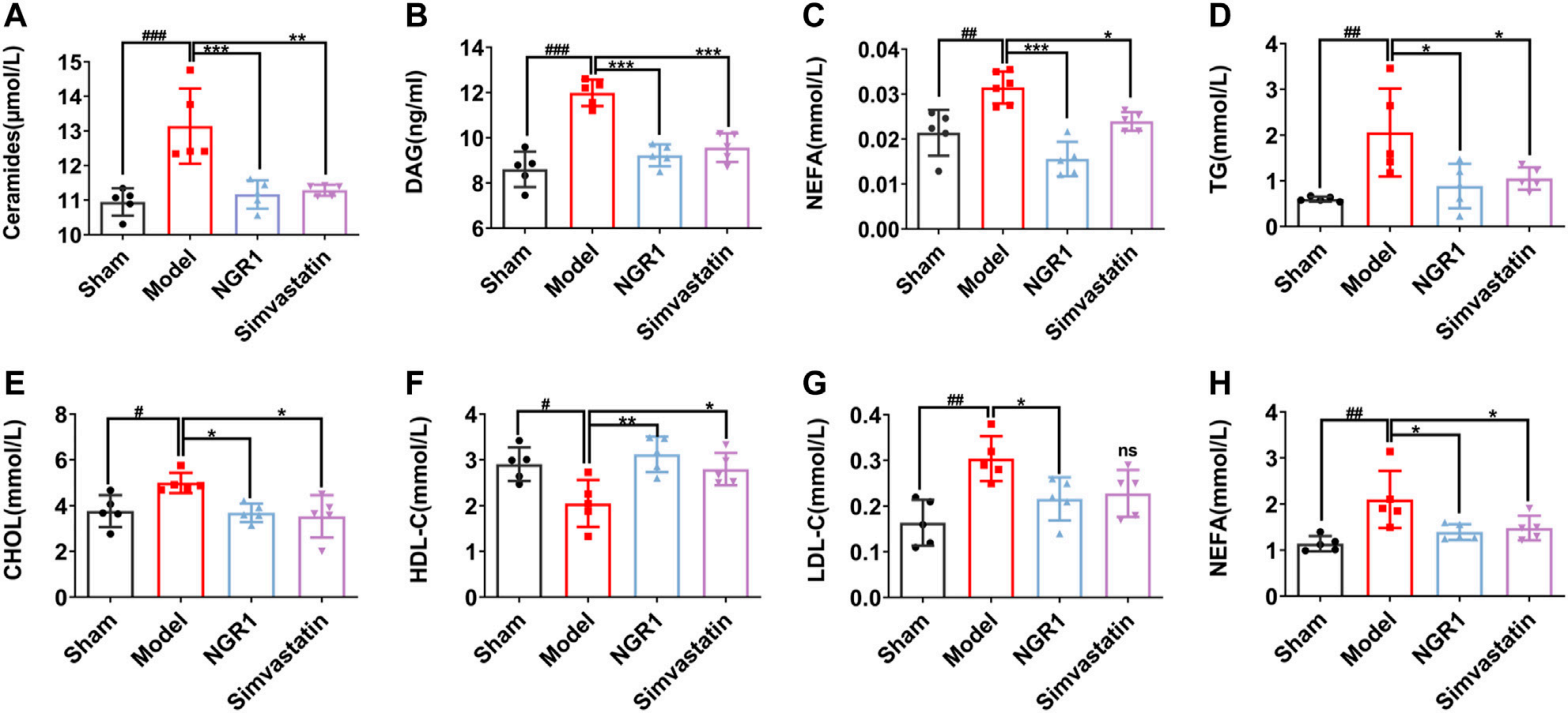
NGR1 regulated serum lipid levels in HF mice model. ELISA was used to detect the levels of lipotoxicity mediators including ceramide, diacylglycerol (DAG) and non-esterified fatty acids (NEFA) in myocardial tissue **(A-C)**. Effects of NGR1 on serum **(D)** triglyceride (TG) **(E)** cholesterol (CHOL) **(H)** high density lipoprotein-cholesterol (HDL-C) **(G)** low density lipoprotein-cholesterol (LDL-C) and **(E)** Non-esterified fatty acids (NEFA) of mice in each group. *p < 0.05, **p < 0.01, ***p < 0.001 vs. model group, ^#^ *p* < 0.05, ^##^ *p* < 0.01, ^###^ *p* < 0.001 vs. sham group, *N* = 5 per group.

Ceramide, DAG and NEFA are considered as lipotoxicity mediators due to their effect on heart dysfunction (D'Souza et al., 2016; Kretzschmar et al., 2021). The levels of ceramide, DAG and NEFA in the model group were up-regulated compared with those in the sham group (*p* < 0.001, *p* < 0.01). After NGR1 intervention, ceramide, DAG and NEFA levels were reduced (*p* < 0.001) (Figures 2A,C). Cardiac lipotoxicity is a manifestation of disorders of cardiac lipid metabolism. Therefore, we used an automated biochemical analyzer to measure serum lipid levels. The results showed that levels of TG, CHOL, LDL-C and NEFA were increased (*p* < 0.05) and HDL-C level was reduced in the model group (*p* < 0.001), as compared with sham group. These results suggest that lipid metabolism disorders occurred in HF mice. NGR1 treatment effectively reversed the changes of the serum lipid levels, demonstrating that NGR1 could regulate lipid metabolism (Figures 2D,H).

### 3.3 NGR1 Regulated Expressions of Lipotoxicity-Related Key Enzymes and Attenuated Lipotoxicity in the HF Mice Model

**FIGURE 3:**
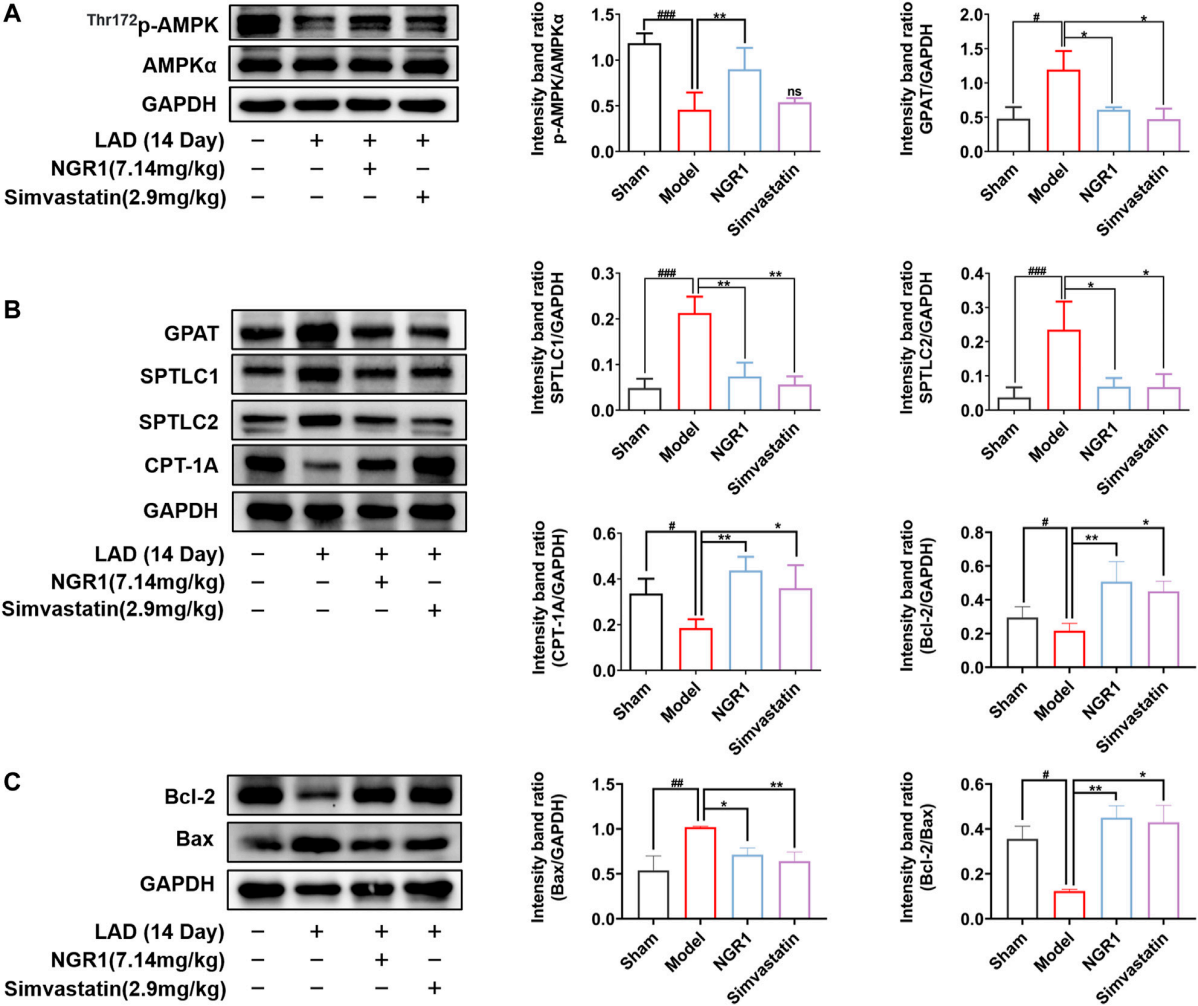
NGR1 regulated lipotoxicity-related proteins and reduced apoptosis in the HF mice model. (**A-C)**Western blot analysis of p-AMPK, AMPK, GPAT, SPTLC1, SPTLC2, CPT-1A, Bcl-2 and Bax in each group of mice. GAPDH served as the internal control, *p < 0.05, **p < 0.01 vs. model group, ^#^ *p* < 0.05, ^##^ *p* < 0.01, ^###^ *p* < 0.001 vs. sham group, ns stands for not statistically significant, *N* = 3-5 per group.

AMPK is an important regulator of energy homeostasis in cardiomyocyte. The α subunit was the main catalytic subunit of AMPK and it could be activated when phosphorylated at Thr172 site (Feng et al., 2018). Activated AMPK can effectively reduce myocardial dysfunction, inhibit myocardial cell apoptosis and attenuated lipotoxicity by regulating downstream targets, including SPT and GPAT. We investigated whether NGR1 attenuated cardiac lipotoxicity in HF mice by regulating the AMPK pathway. We found that the expressions of p-AMPK were significantly decreased (*p* < 0.001) and the expressions of GPAT, SPTLC1 and SPTLC2 were all increased in the model group (*p* < 0.05, *p* < 0.001) compared with sham group, suggesting that production of toxic lipids were increased in HF mice heart. In addition, the expression of CPT-1A was significantly down-regulated in the model group (*p* < 0.05), suggesting that FAO was impaired in the hearts of model mice. NGR1 treatment could regulate the expressions of p-AMPK and key enzymes involved in toxic lipid production and lipid metabolic pathway (*p* < 0.05, *p* < 0.01) **(** Figures 3A,B **)**. We further examined the expression of anti-apoptotic protein Bcl-2 and pro-apoptotic protein Bcl-2 Associated X Protein (Bax). The results showed that NGR1 treatment could regulate expressions of Bcl-2 and Bax (*p* < 0.05, *p* < 0.01), thereby attenuating the lipotoxicity-induced apoptosis (Figure 3C).

### 3.4 NGR1 Increased Cell Viability in PA-Stimulated H9C2 Cells *in vitro*

**FIGURE 4:**
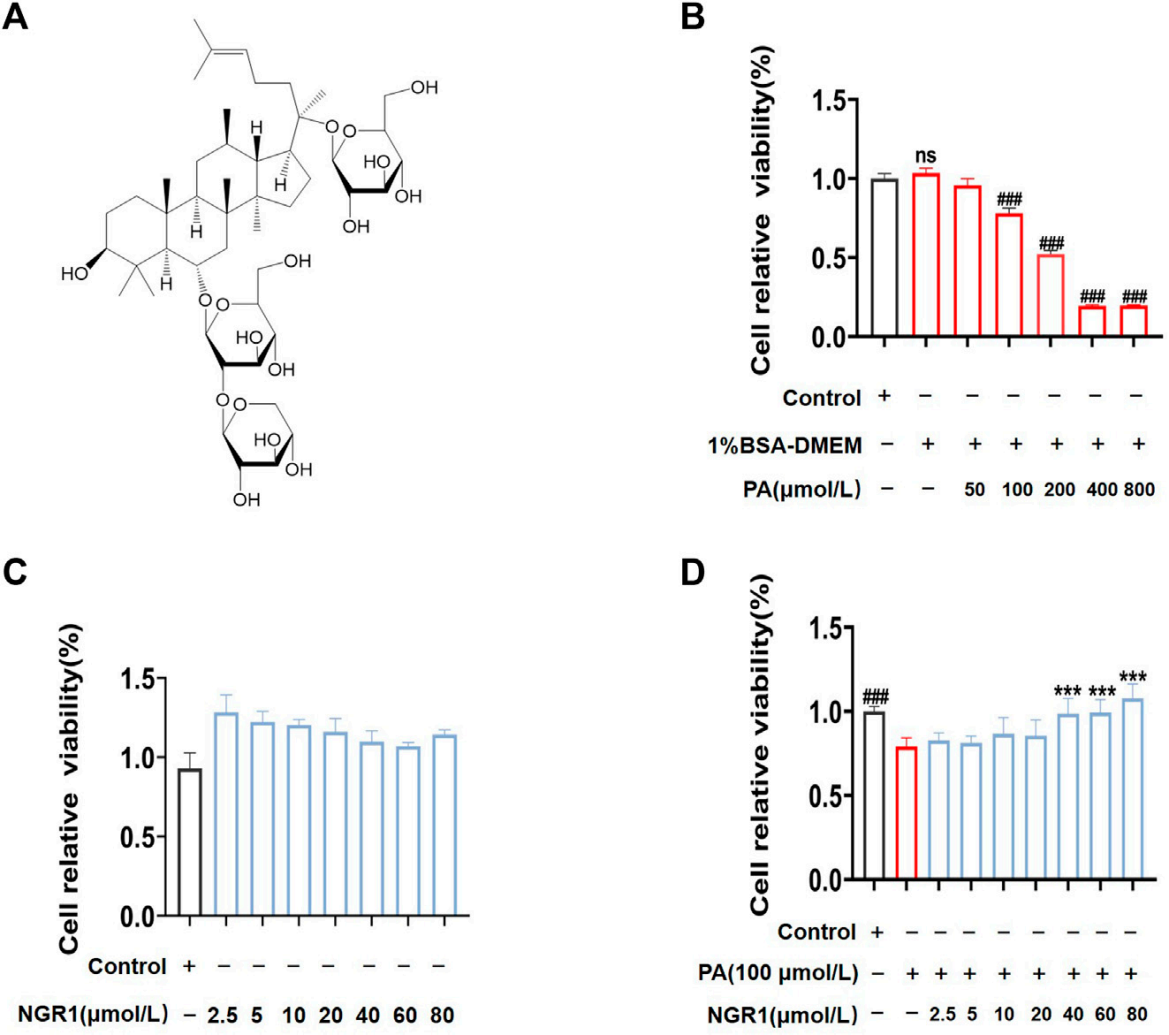
NGR1 increased the survival rate in PA-induced lipotoxicity H9C2 cell model. **(A)** Chemical structure formula of NGR1 **(B)** PA reduced cell viability in a concentration-dependent manner by CCK-8 assay. **(C)** H9C2 cells were incubated with different dosage (2.5 μmol/L to 80 μmol/L) of NGR1 for 24 h. CCK-8 assay was used to detect cell viability. **(D)** Cells were co-incubated with NGR1 (dose from 2.5 μmol/L to 80 μmol/L) and PA (100 μmol/L) for 24 h, then cell viability were detected by CCK-8 assay. ***p < 0.001 vs. model group, ^###^ *p* < 0.001 vs. control group, ns stands for not statistically significant, repeat the experiment three times independently.

To further verify the potential regulatory mechanisms of NGR1 on lipotoxicity, we established a PA-stimulated H9C2 cell model. When stimulated cells for 24 h, we found that cell viability significantly decreased in a dose-dependent manner (*p* < 0.001) (Figure 4B). BSA was used to solubilize PA, so we compared BSA with the normal group and found that it had no effect on cell viability (Figure 4B). Based on CCK8 results, 100 μmol/L PA was chosen as working concentration to induce lipotoxicity in H9C2 cells for subsequent experiments (Figure 4B). The effect of NGR1 on PA-stimulated H9C2 lipotoxicity cell model were investigated. The CCK-8 assay results showed that NGR1 was not toxic to cells at concentrations ranging from 2.5 μmol/L to 80 μmol/L. NGR1 increased cell viability in PA-stimulated cells in a dose-dependent manner and 40 μmol/L was chosen as working concentration for subsequent investigations (Figures 4C,D).

### 3.5 NGR1 Regulated Lipid Metabolisms in PA-Stimulated Cells Through AMPK Signaling Pathway

**FIGURE 5:**
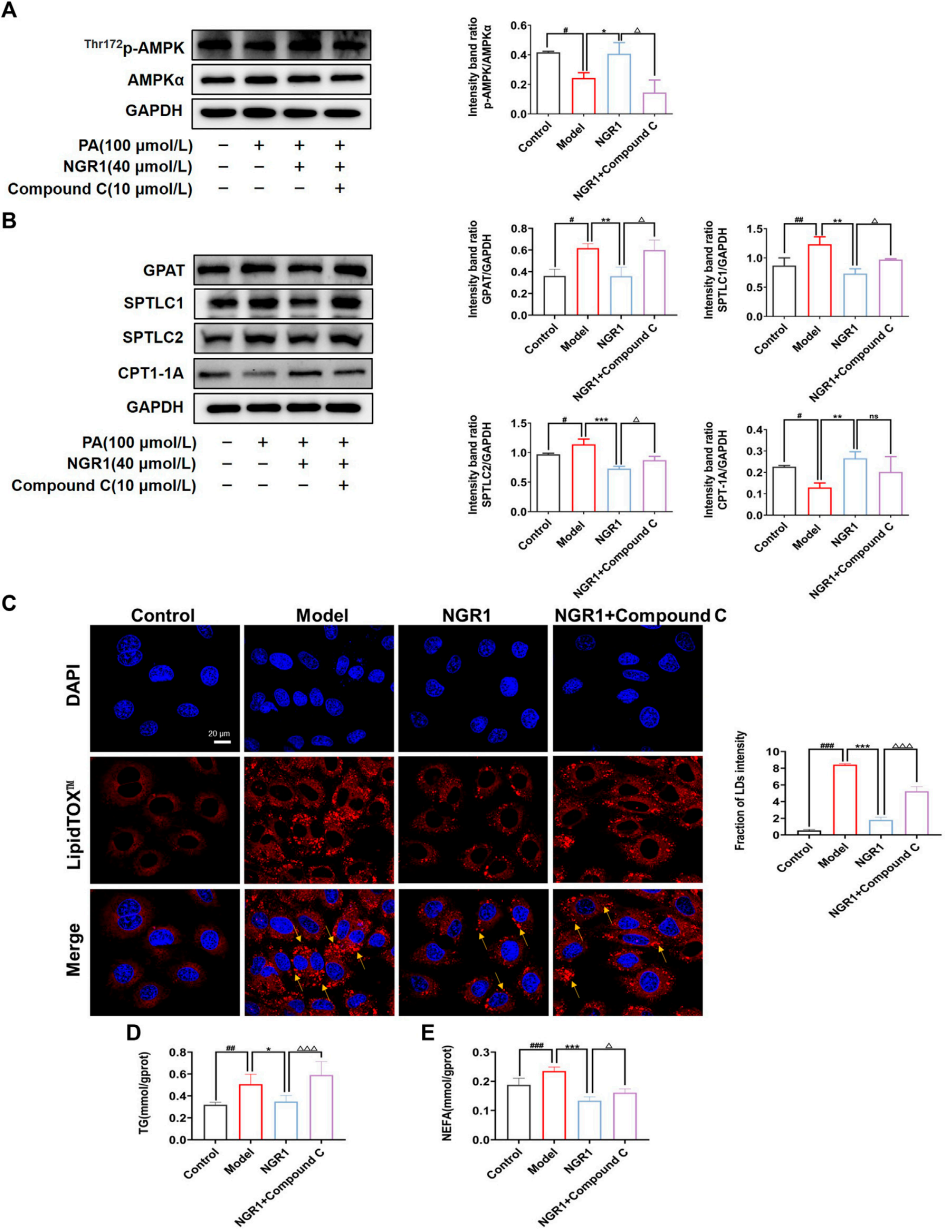
NGR1 reduced cardiac lipotoxicity through AMPK pathway *in vitro*. **(A)** NGR1 could increase the expression of p-AMPK in PA-induced model. **(B)** Western blots analysis of GPAT, SPTLC1, SPTLC2, CPT-1A protein levels in PA-induced H9C2 model treated with NGR1. **(C)** Representative images of LipidTOX™ staining in PA-induced H9C2 cells with the treatment of NGR1. Scale bar = 20 μm. **(D)** TG assay results showed NGR1 could decrease TG content in cells. **(E)** NEFA assay results showed NGR1 could decrease NEFA content in PA-induced model. When cells were incubated with Compound C, the effect of NGR1 on cardiac lipotoxicity were aboragated. *p < 0.05, **p < 0.01, ***p < 0.001 vs. model group, ^#^ *p* < 0.05, ^##^ *p* < 0.01, ^###^ *p* < 0.001 vs. control group, ^△^ *p* < 0.05, ^△△△^ *p* < 0.001 vs. NGR1 group, ns stands for not statistically significant. The experiments were repeated three times independently.

The effects of NGR1 in a PA-stimulated cell model were further investigated. As shown in Figure 5A, PA treatment significantly down-regulated the expression of p-AMPK (*p* < 0.05). Moreover, the expressions of GPAT, SPTLC1 and SPTLC2 were significantly up-regulated (*p* < 0.05, *p* < 0.01) and the expression of CPT-1A (*p* < 0.05) was down-regulated by PA challenge (Figure 5B). Impressively, NGR1 treatment could reverse these changes and regulate expressions of p-AMPK and enzymes involved in lipid metabolism (Figures 5A,B). Moreover, when cells were co-treated with NGR1 and Compound C, an inhibitor of AMPK, the regulative effects of NGR1 on lipid metabolic enzymes were abrogated, demonstrating that the effects of NGR1 on lipid metabolism are mediated by AMPK (Figure 5B).

We also observed lipid accumulation in PA-stimulated cells, as shown by LipidTOX™ staining (Figure 5C). Lipid accumulation might be caused by reduced levels of FAO. NGR1 treatment could reduce lipid accumulation in H9C2 cells, whereas AMPK inhibitor could abrogate the effects of NGR1 (Figure 5C). We further assessed the levels of TG and NEFA. NGR1 could significantly reduce the levels of TG and NEFA in PA stimulated H9C2 cells (*p* < 0.01, *p* < 0.001), whereas AMPK inhibitor ameliorated the regulative effects of NGR1. These results demonstrated that NGR1 could attenuate lipid accumulation in PA-stimulated cells and this effect was mediated via AMPK pathway.

### 3.6 NGR1 Attenuated Lipotoxicity-Induced Oxidative Stress and Apoptosis by AMPK Pathway *in vitro*

**FIGURE 6:**
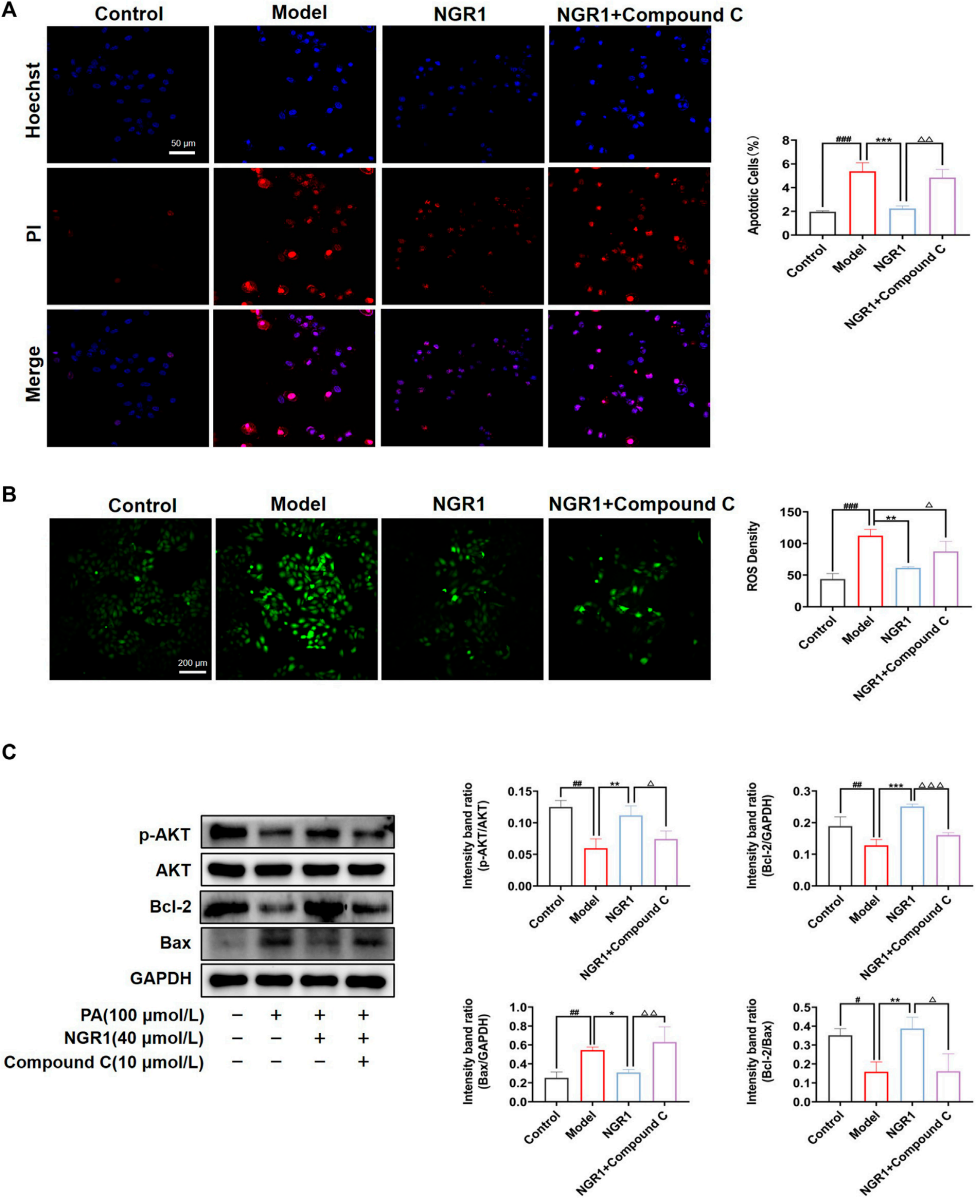
Effects of NGR1 on oxidative stress and apoptosis. **(A)** Representative images of Hoechst 33342/PI staining and quantitative analysis. Scale bar = 50 μm. **(B)** Representative images of ROS staining in PA-induced H9C2 cells with the treatment of NGR1. Scale bar = 200 μm. **(C)** Western blot analysis of p-AKT, AKT, Bcl-2 and Bax protein levels in H9C2 treated with NGR1. GAPDH served as the internal control, *p < 0.05, **p < 0.01, ***p < 0.001 vs. model group, ^#^ *p* < 0.05, ^##^ *p* < 0.01, ^###^ *p* < 0.001 vs. control group, ^△^ *p* < 0.05, ^△△^ *p* < 0.01, ^△△△^ *p* < 0.001 vs. NGR1 group. The experiments were repeated three times independently.

Imbalance of lipid metabolism induces the accumulation of ceramide and DAG, which in turn leads to oxidative stress and apoptosis. The effect of NGR1 on apoptosis was assessed by Hoechst/PI staining in a PA-induced lipotoxicity model. As shown in Figure 6A, fragmented, brighter fluorescence was observed in H9C2 cells after 24 h of PA stimulation, indicating a high level of intracellular apoptosis. NGR1 significantly attenuated the apoptotic rate (Figure 6A). The DCFH-DA probe was used to detect intracellular ROS levels, and the results showed that NGR1 significantly attenuated the increase in ROS levels induced by PA (Figure 6B). Ceramide and DAG have been reported to reduce Bcl-2 expression by inhibiting protein kinase B (AKT) phosphorylation, thereby exacerbating the apoptotic response. In this study, we found that the expression of p-AKT was significantly down-regulated in the model group compared to the normal group (*p* < 0.01). These alterations were reversed after NGR1 treatment (Figure 6C). These results indicated that lipotoxicity-induced apoptosis and oxidative stress could be inhibited by NGR1. Moreover, the effects of NGR1 on lipotoxicity-induced apoptosis and ROS production were abrogated by AMPK inhibitor, further demonstrating that the protective effects of NGR1 were mediated by AMPK pathway.

## 4 Discussion

Accumulation of toxic lipids and abnormal fatty acids metabolisms have been observed in diabetes, obesity and cardiovascular diseases. Recent studies indicate that lipotoxicity may lead to HF and could serve as potential treatment targets (Schulze et al., 2016). Some herbal components have been shown to have regulatory effects on lipid metabolisms (Zhang et al., 2018). In this study, we established an animal HF model and lipotoxicity H9C2 cell model to explore the effects and mechanisms of NGR1 on lipotoxicity. Both abnormal FAO and accumulation of toxic lipids were observed in HF mice model. NGR1 treatment could improve cardiac function, alleviate myocardial pathological changes and ameliorate cardiac lipotoxicity in the HF mice model. *In vitro* studies demonstrated that NGR1 could enhance cell viability, reduce lipid accumulation, attenuate apoptosis and ameliorate oxidative stress. Moreover, we discovered that NGR1 reduced cardiac lipotoxicity via AMPK signaling pathway.

Cardiomyocytes preferentially use FA to meet high energy needs, indicating the importance of FA metabolism in the heart (Schulze et al., 2016). Imbalance of lipid uptake and utilization in cardiomyocytes can lead to lipid accumulation in the myocardium. Ceramide is considered as one of the potential lipotoxicity molecules, which promote development of cardiovascular diseases (Park et al., 2008). Ceramide can initiate the intrinsic apoptotic pathway by permeablizing mitochondrial membrane, which can release cytochrome c and apoptosis-inducing factors to the cytosol resulting in apoptosome formation and activation of caspase-3. DAG is another potential molecule that can mediate lipotoxicity (D'Souza et al., 2016). Clinical studies found that DAG accumulation occurred in the heart of patients with severe HF (Liu L. et al., 2014). DAG could activate the pro-apoptotic protein PKCδ, inhibit phosphorylation of AKT, and induce apoptosis. In this study, we revealed that NGR1 down-regulated the expression levels of both ceramide and DAG in myocardial tissue of mice, indicating that NGR1 could attenuate HF by reducing toxic lipids. In addition, NGR1 could reduce serum level of TG in HF mice model and TG accumulation in PA-stimulated H9C2 cells, demonstrating that NGR1 could regulate oxidation of lipids in cardiomyocytes.

SPT is considered as a key enzyme regulating sphingolipid levels in cells, and regulation of SPT may prevent the harmful accumulation of ceramide (Hanada 2003). GPAT play an important role in the *de novo* synthesis of DAG (Kojta et al., 2020). CPT-1A is responsible for the transfer of FA into mitochondria and is the key enzyme in the oxidation of FA (Lundsgaard et al., 2020). In this study, we found that expressions of SPT and GPAT were up-regulated in HF mice model and H9C2 cells. In addition, expression of CPT-1A was down-regulated. Treatment with NGR1 could down-regulate expressions of SPT and GPAT, which in turn reduced production of ceramide and DAG. NGR1 also up-regulated expression of CPT-1A, which promoted FAO and reduced accumulation of lipid droplets. Some other studies also showed that regulating key lipid-metabolizing enzymes could efficiently reduce accumulation of toxic lipids and attenuate lipotoxicity (Ji et al., 2017). We further explored the effects of NGR1 on the upstream regulator AMPK. The role of AMPK in cardiovascular diseases have been extensively investigated. More and more studies have confirmed that AMPK has beneficial effects in regulating lipotoxicity in a variety of diseases (Li et al., 2020; Zuo et al., 2020). The latest research showed that activation of AMPK can effectively reduce apoptosis and oxidative stress induced by lipotoxicity (Zuo et al., 2020). AMPK may serve as a potential target for the treatment of cardiac lipotoxicity. In this study, we found that the expression of p-AMPK was significantly decreased in the model group in both *in vivo* and *in vitro* experiments. When the AMPK inhibitor Compound C was co-incubated with NGR1, the effect of NGR1 on lipotoxicity was abolished. These results suggested that NGR1 exerted its lipid-regulative effect by activating AMPK pathway. Excessive accumulation of toxic lipids and other lipid intermediates can cause oxidative stress, apoptosis and mitochondrial disorders. We observed an increase in apoptosis and ROS production levels in the PA-stimulated cell model. NGR1 treatment could effectively attenuate ROS production and cellular apoptosis when cells were co-treated with AMPK inhibitor, the protective effects of NGR1 were abrogated, further demonstrating that NGR1 exerted anti-lipotoxic effects by activating AMPK pathway.

NGR1 is one of the main active components in *P. notoginseng* and previous studies have shown that NGR1 could attenuate myocardial injury and reduce myocardial infarct size in an ischemia/reperfusion injury model (Tong et al., 2019), which may be through Nrf2-HO-1 or mTOR induced inflammation and apoptosis signaling pathway (Du et al., 2021; Liu et al., 2021). However, its mechanism on AMPK mediated lipotoxicity in AMI-post heart failure has not been described before. In our study, we demonstrated that NGR1 could enhance p-AMPK expression in MI mice and cells model, and for the first time, we investigated the protective effect of NGR1 on myocardial lipotoxicity via AMPK signaling pathway from *in vitro* and *in vivo* studies. Meanwhile, the crucial role of AMPK in the protective role of NGR1 was further confirmed by the use of AMPK inhibitors. In addition, we also found that the activation of AMPK could effectively reduce the apoptosis and oxidative stress caused by lipotoxicity mechanismally.

**FIGURE 7:**
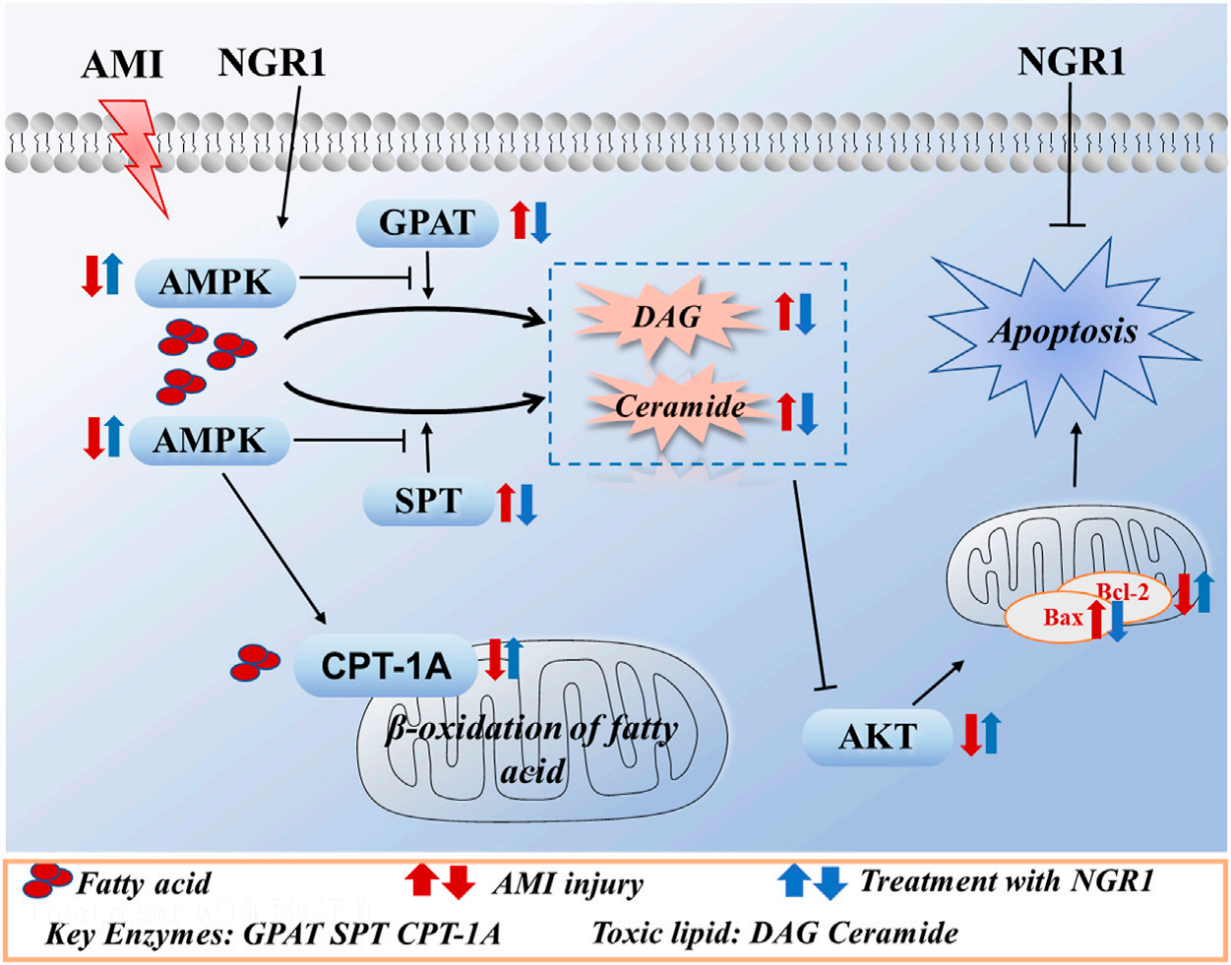
The potential mechanism of NGR1 in attenuating lipotoxicity through AMPK signal pathway.

In summary, our study demonstrated that NGR1 could exert cardioprotective effects against lipotoxicity in HF mice model. The protective effects were mediated by upregulation of AMPK pathway. NGR1 could improve oxidation of fatty acids and reduce toxic lipids levels, thereby inhibiting lipotoxicity-induced apoptosis (Figure 7). AMPK is a key player in attenuating cardiac lipotoxicity by NRG1 and may serve as a target for the management of HF.

## Data Availability

The original contributions presented in the study are included in the article/Supplementary Material, further inquiries can be directed to the corresponding authors.

## Abbreviations

AKT, protein kinase B; AMI, acute myocardial infarction; AMPK, adenosine 5′ -monophosphate-activated protein kinase; ANOVA, one-way analysis of variance; Bcl-2 Associated X Protein, Bax; B cell lymphoma-2, Bcl-2; BSA, bovine serum albumin; CCK-8, cell counting kit-8; CK-MB, creatine kinase MB; CHOL, cholesterol; cTnI, cardiac troponin I; CPT-1A, carnitinepalmitoyltransferase 1A; DAG, diacylglycerol; DAPI, 4′,6′- Diamidino-2-phenylindole; DMEM, Dulbecco’s modified Eagle’s medium; EF, ejection fraction; FAO, fatty acid oxidation; FBS, fetal bovine serum; FS, fractional shortening; GPAT, glycerol-3-phosphate acyltransferases; HDL-C, high density lipoprotein cholesterol; H&E, hematoxylin-eosin; HF, heart failure; LAD, left anterior descending artery; LDL-C, low density lipoprotein cholesterol; LVIDd, left ventricular internal diameter at end-diastole; LVIDs, left ventricular internal diameter at end-systole; MF, myocardial fibrosis; MI, myocardial infarction; NEFA, non-esterified fatty acids; NGR1, Notoginsenoside R1; PA, palmitic acid; PBS, phosphate buffer saline; SPF, specific pathogen-free environment; SPT, serine palmitoyltransferase; SPTLC1, serine-palmitoyl transferase long chain 1; SPTLC2, serine-palmitoyl transferase long chain 2; TG, triglyceride.
