# Supplementary figures and images for: Notoginsenoside R1 Ameliorates Cardiac Lipotoxicity Through AMPK Signaling Pathway
Source: Front Pharmacol. 2022 Mar 17;13:864326. doi: 10.3389/fphar.2022.864326 (PMC8968201; doi:10.3389/fphar.2022.864326)

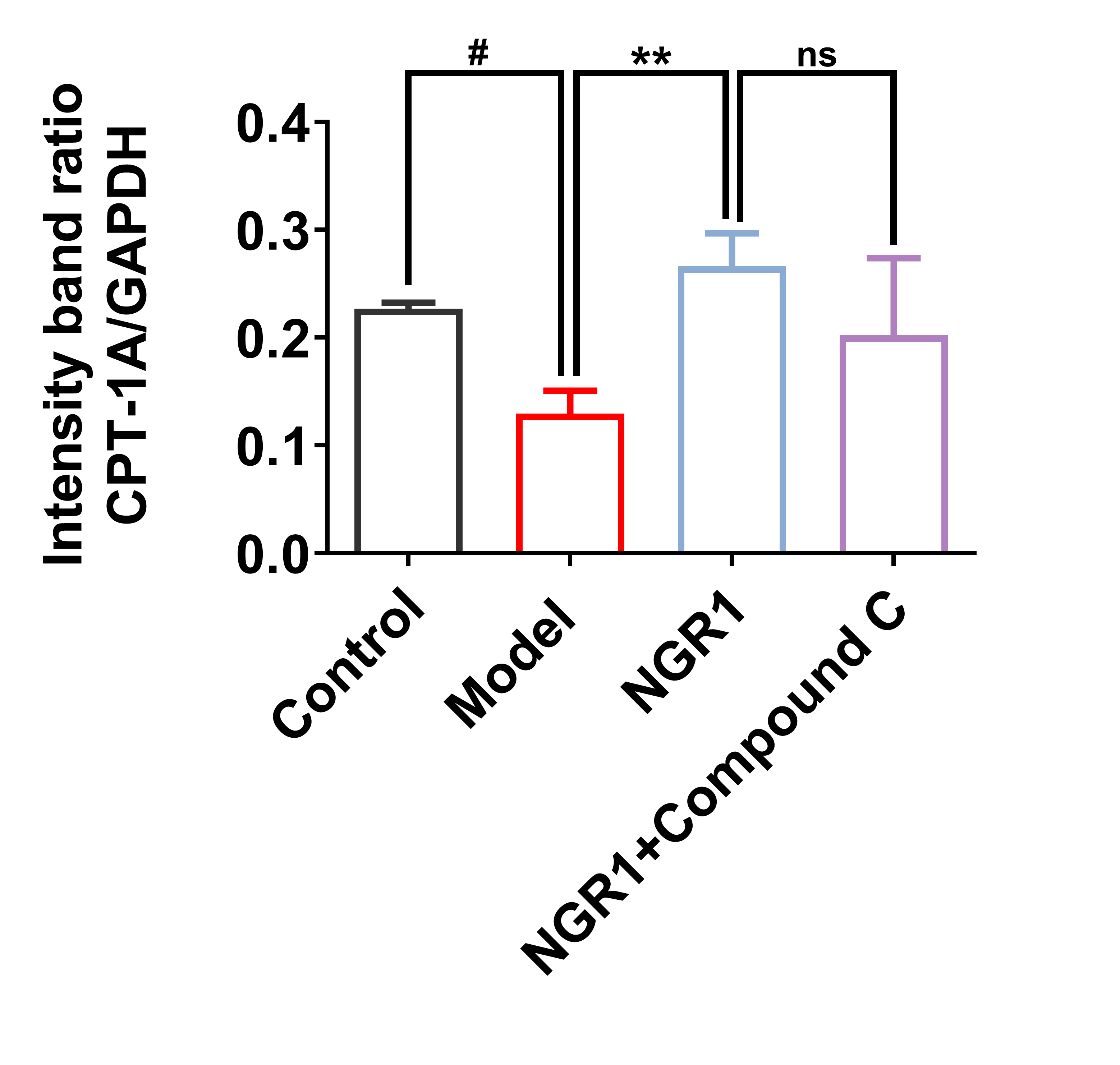

Supplement: Supplementary file 1 [file DataSheet3.ZIP › CPT-1A.tif]

## Slide 1
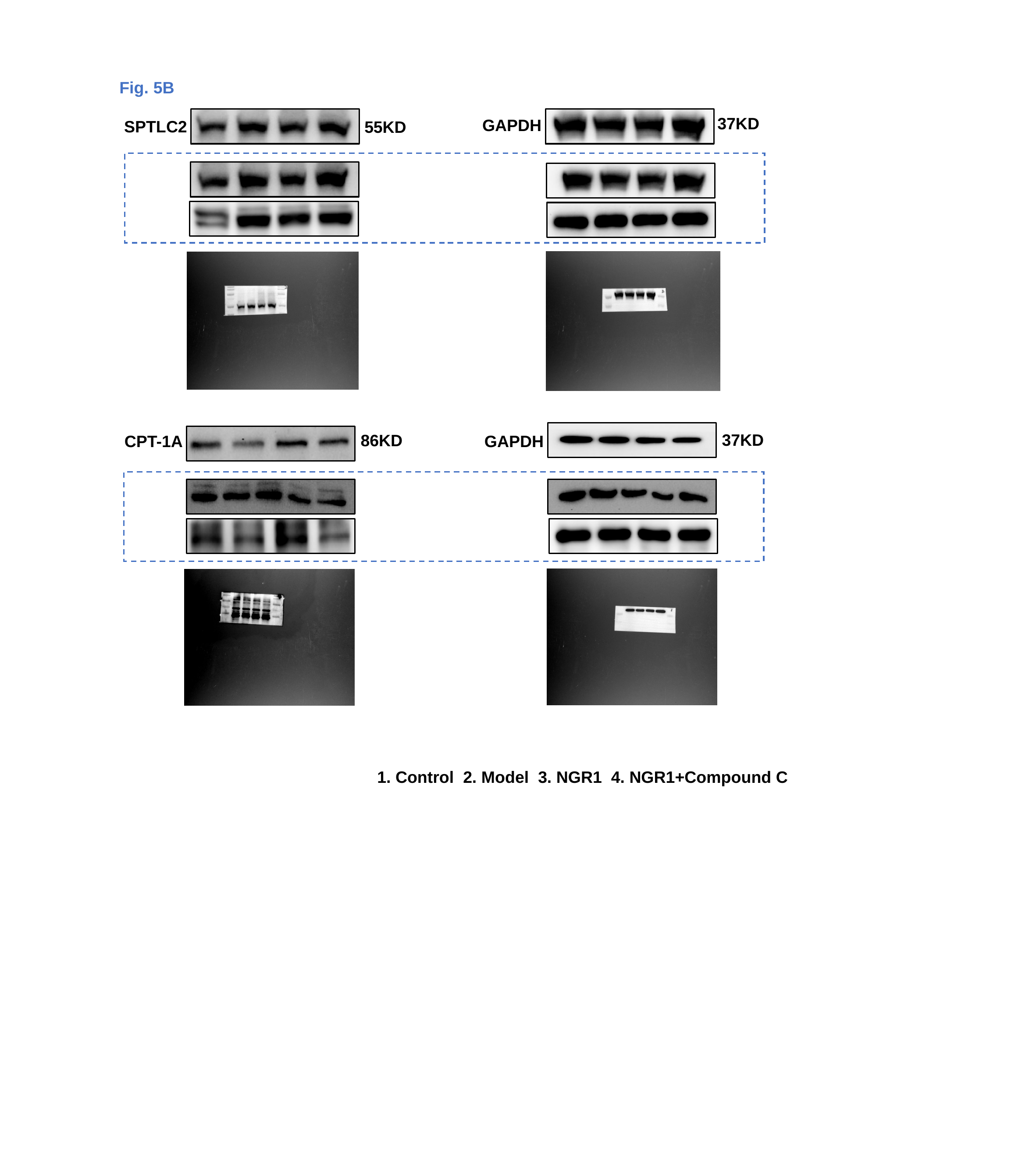

Fig. 5B
SPTLC2
55KD
37KD
GAPDH
37KD
GAPDH
86KD
CPT-1A
1. Control 2. Model 3. NGR1 4. NGR1+Compound C

Supplement: Supplementary file 1 [file DataSheet3.ZIP › Figure 5B-2.pptx]

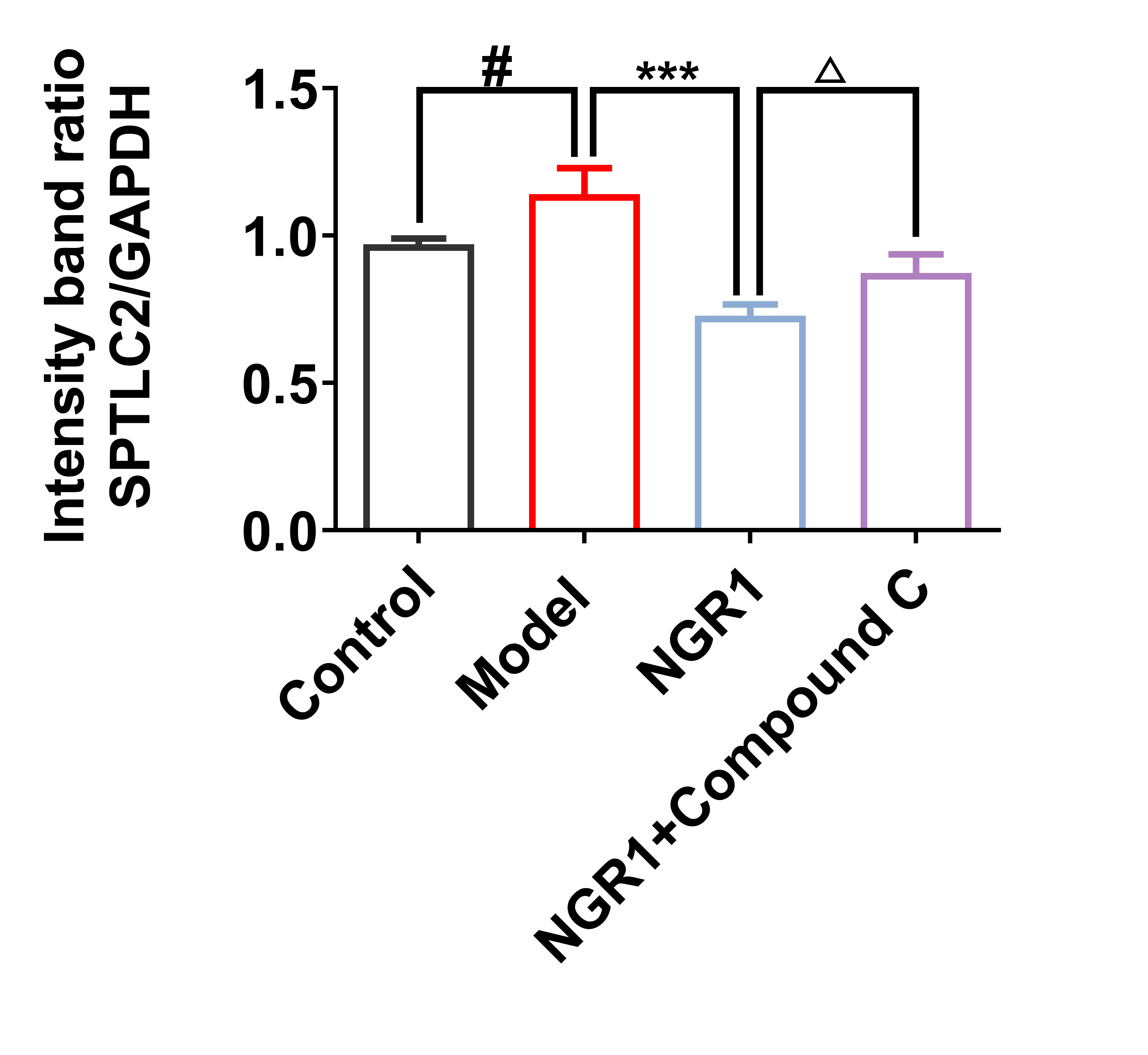

Supplement: Supplementary file 1 [file DataSheet3.ZIP › SPTLC2.tif]

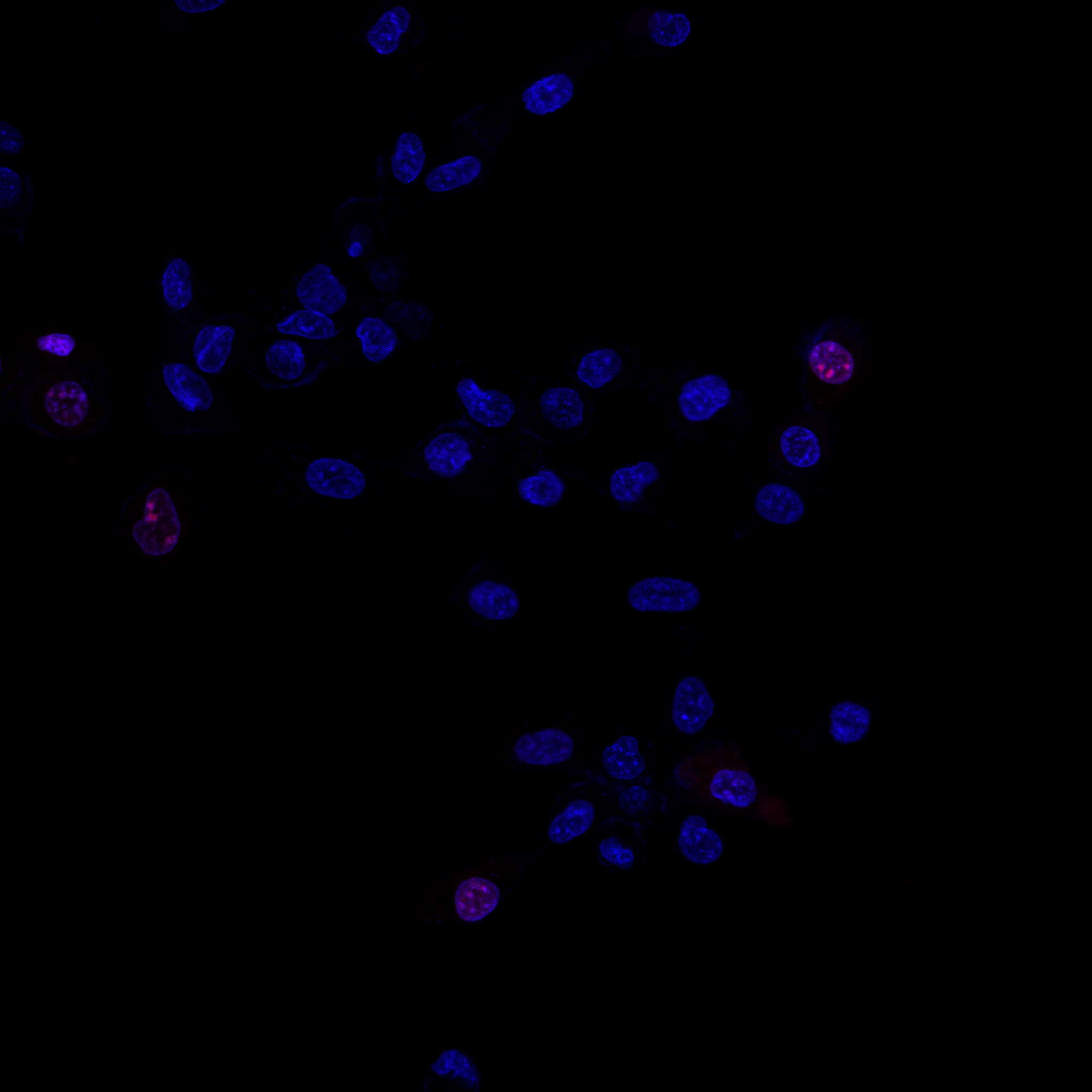

Supplement: Supplementary file 2 [file DataSheet11.ZIP › Fig 6A/Control/c_0018_T001.tif]

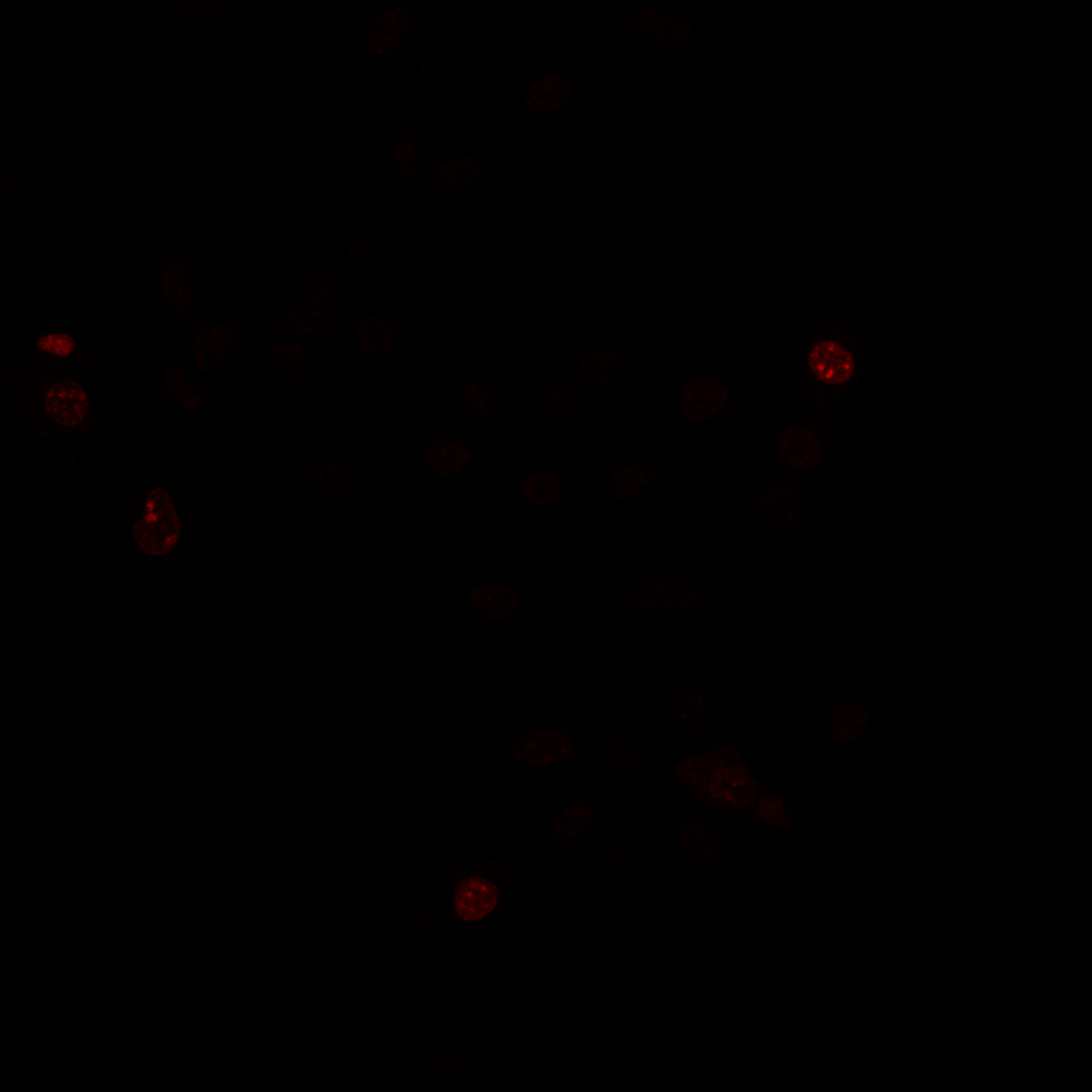

Supplement: Supplementary file 2 [file DataSheet11.ZIP › Fig 6A/Control/Control-PI.tif]

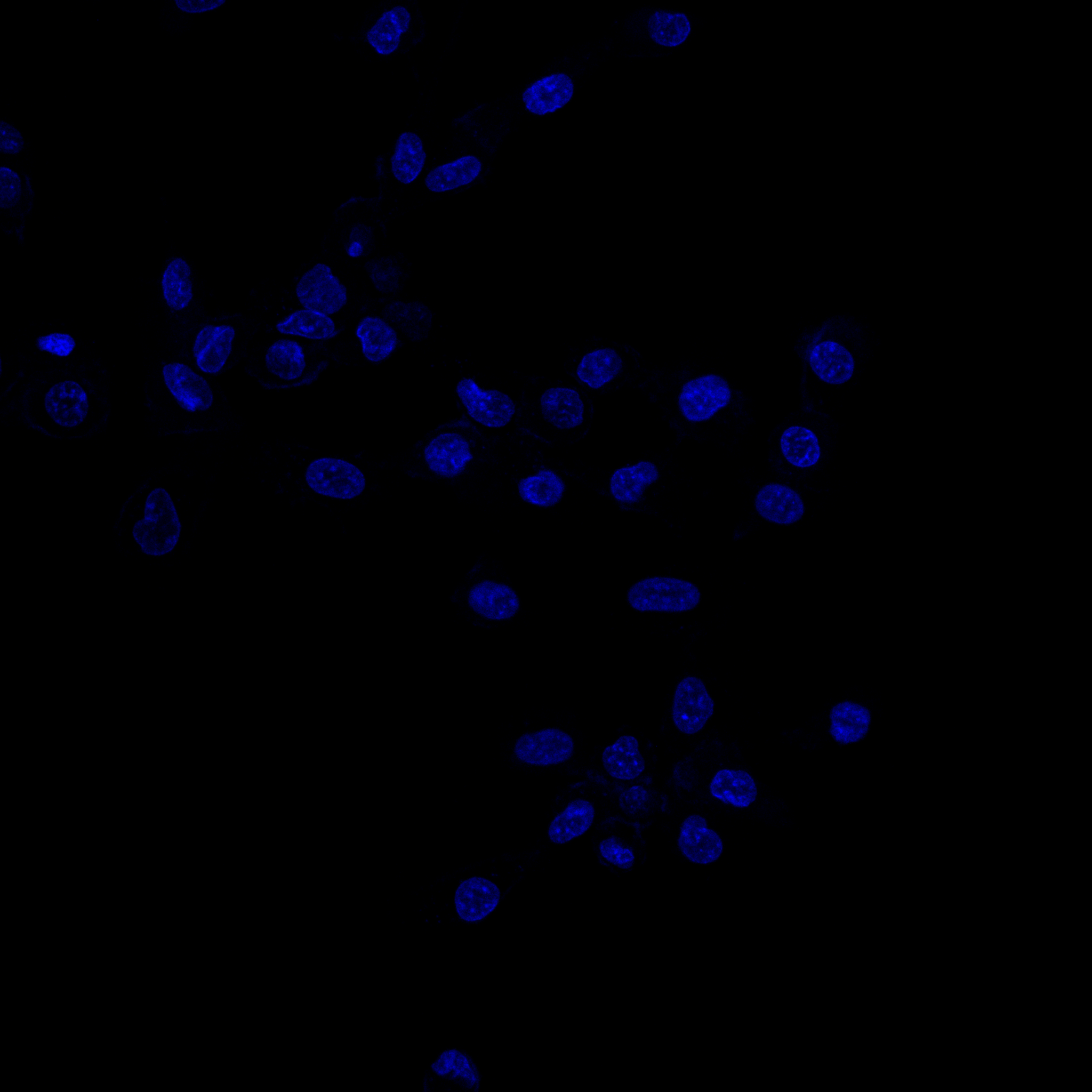

Supplement: Supplementary file 2 [file DataSheet11.ZIP › Fig 6A/Control/Merge.tif]

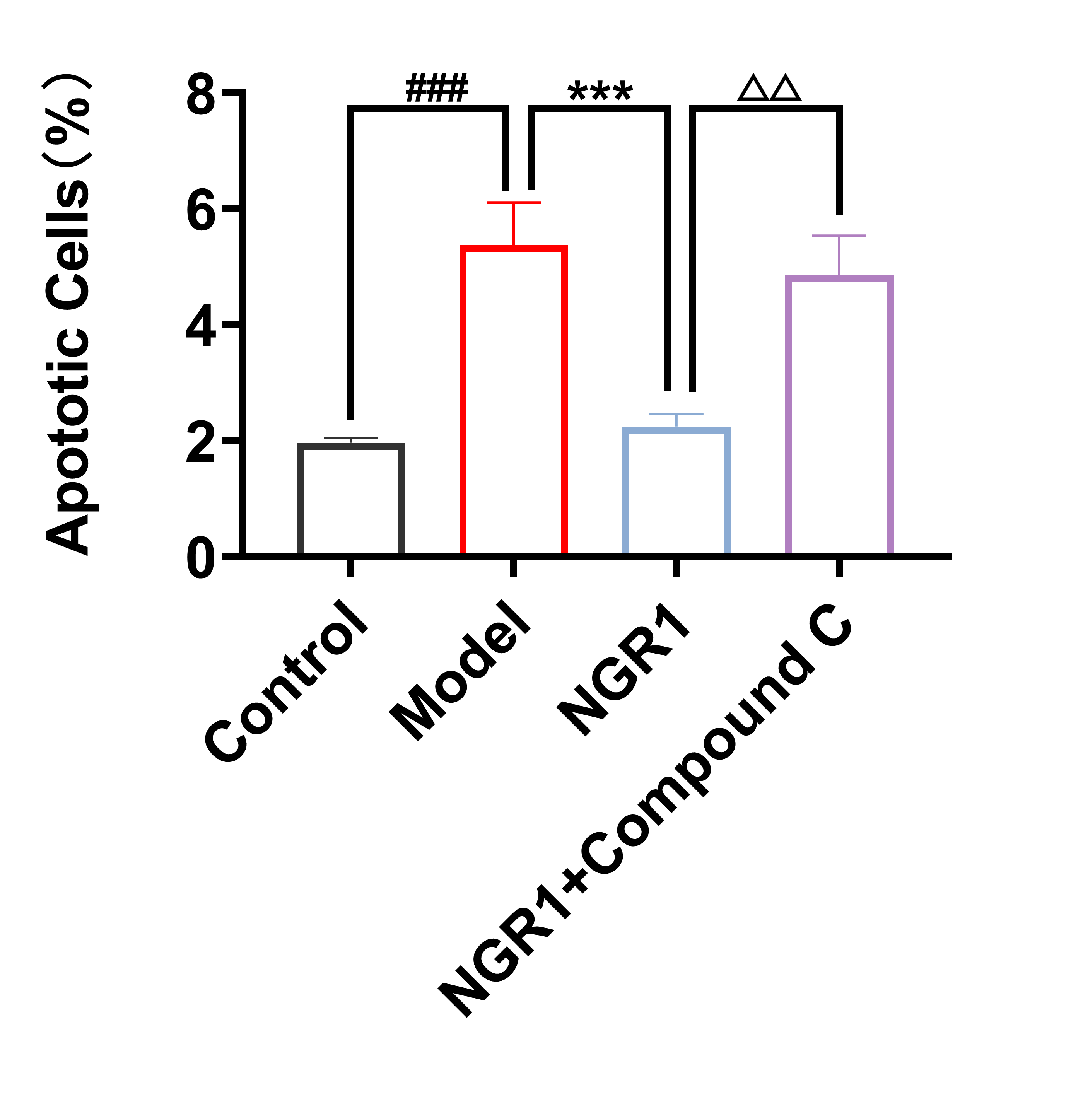

Supplement: Supplementary file 2 [file DataSheet11.ZIP › Fig 6A/Fig 6A.tif]

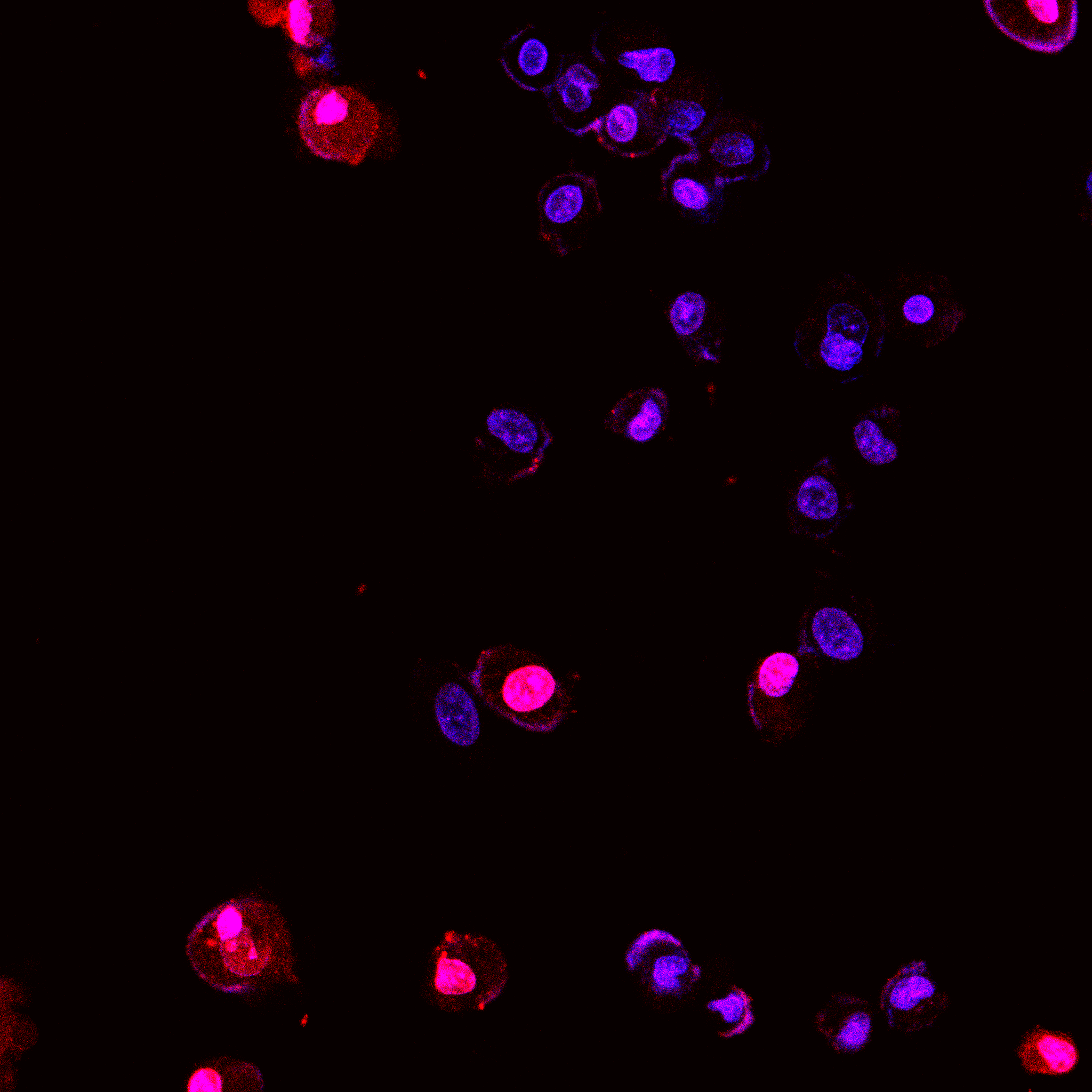

Supplement: Supplementary file 2 [file DataSheet11.ZIP › Fig 6A/Model/Merge.tif]

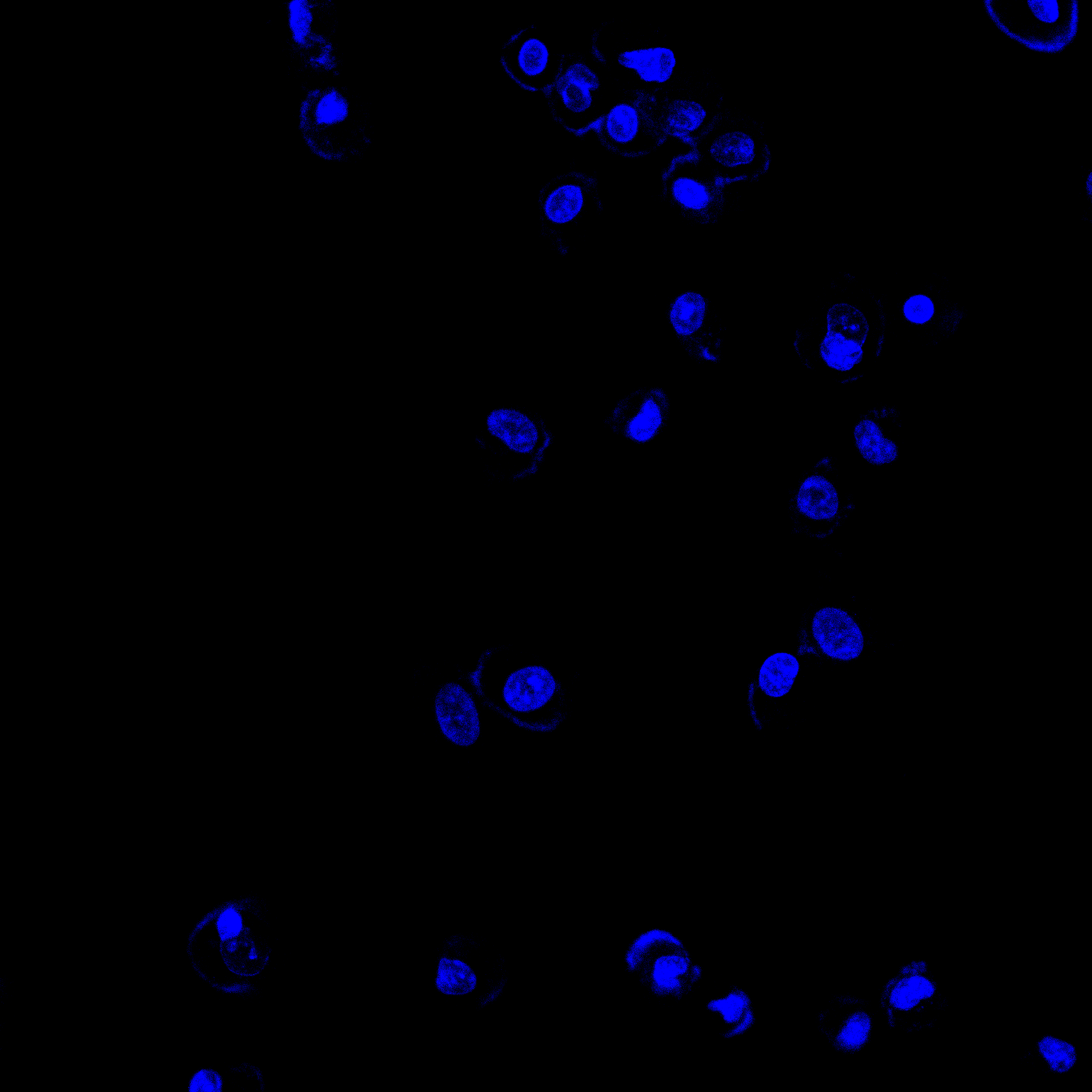

Supplement: Supplementary file 2 [file DataSheet11.ZIP › Fig 6A/Model/Model-Hoechst.tif]

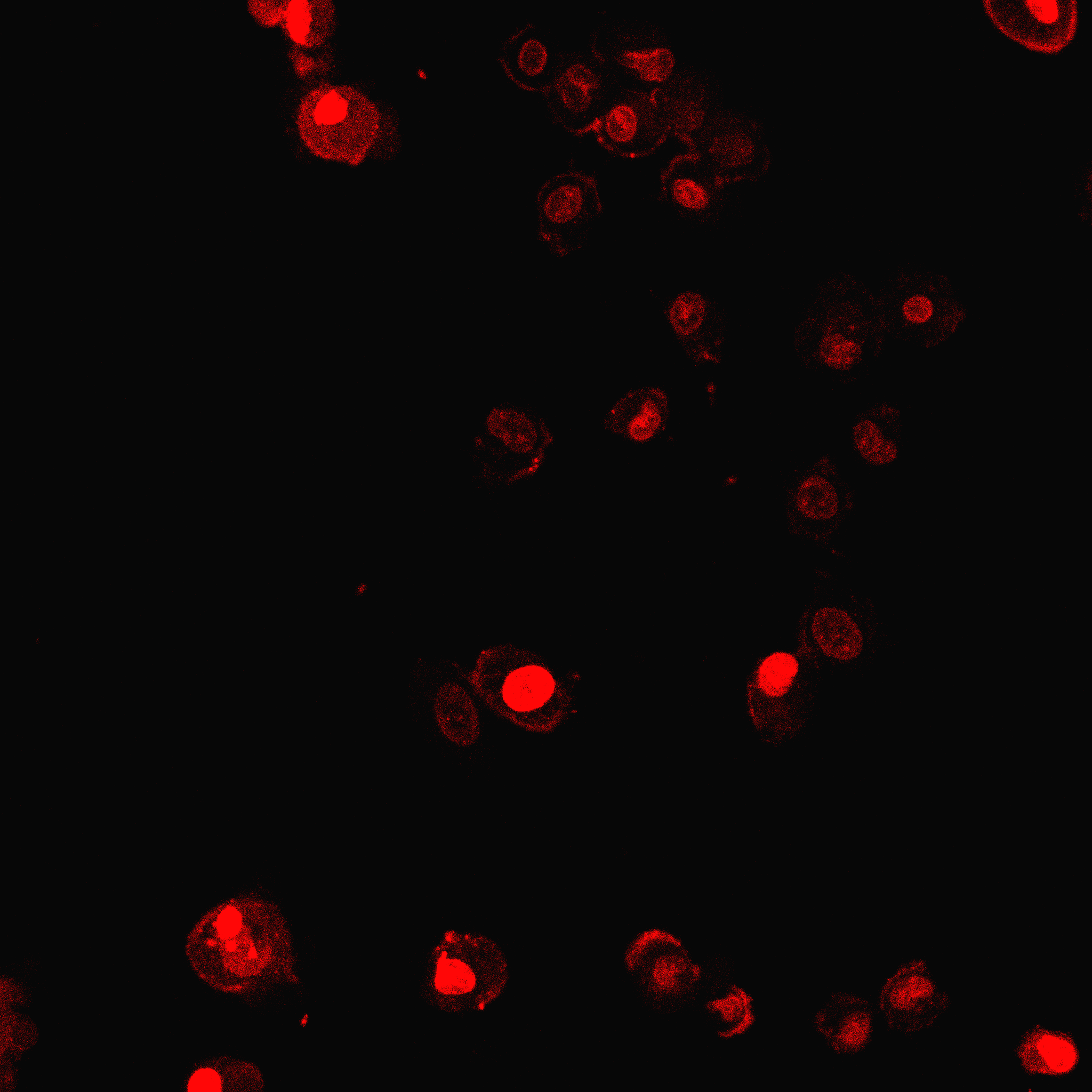

Supplement: Supplementary file 2 [file DataSheet11.ZIP › Fig 6A/Model/Model-PI.tif]

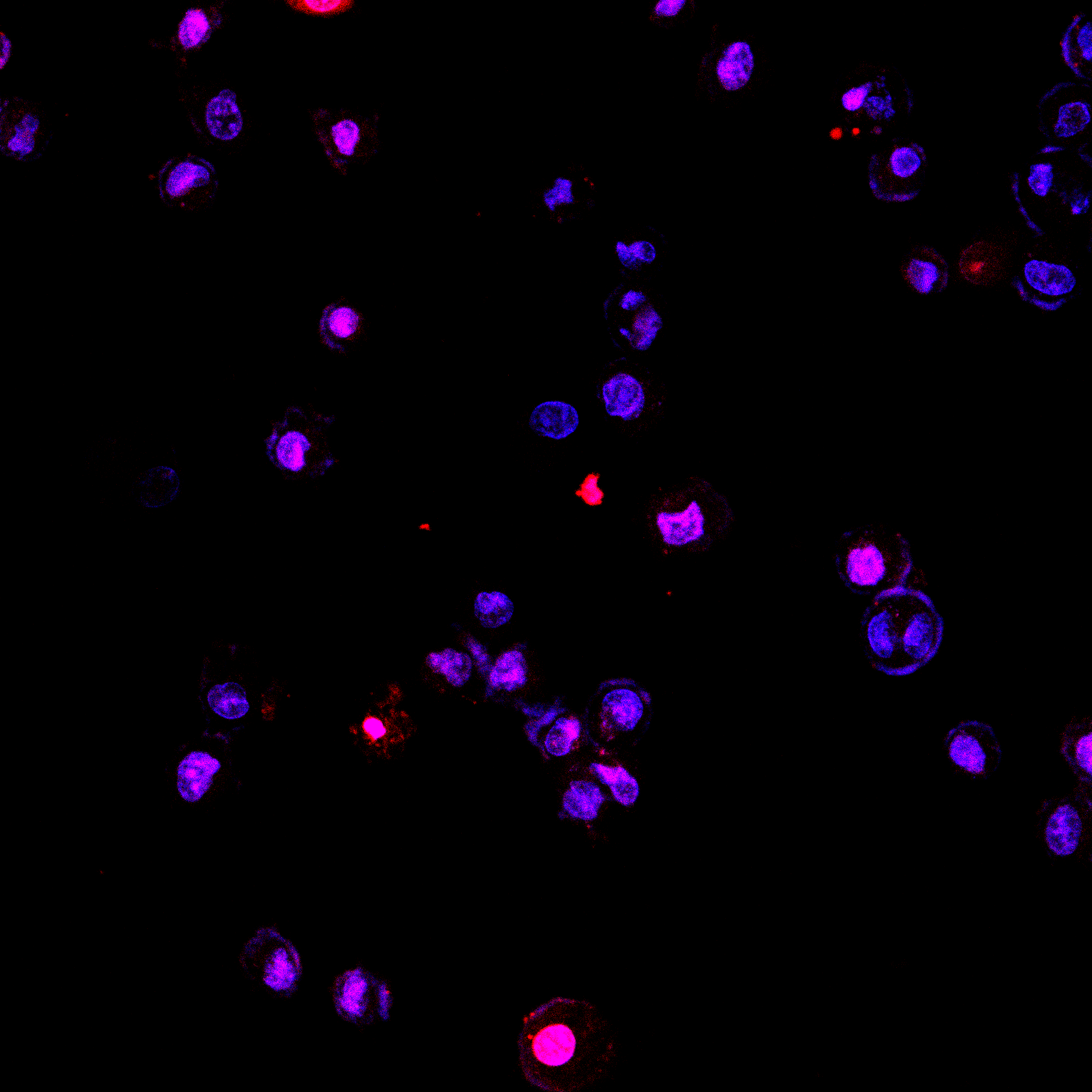

Supplement: Supplementary file 2 [file DataSheet11.ZIP › Fig 6A/NGR1+Compound C/Merge.tif]

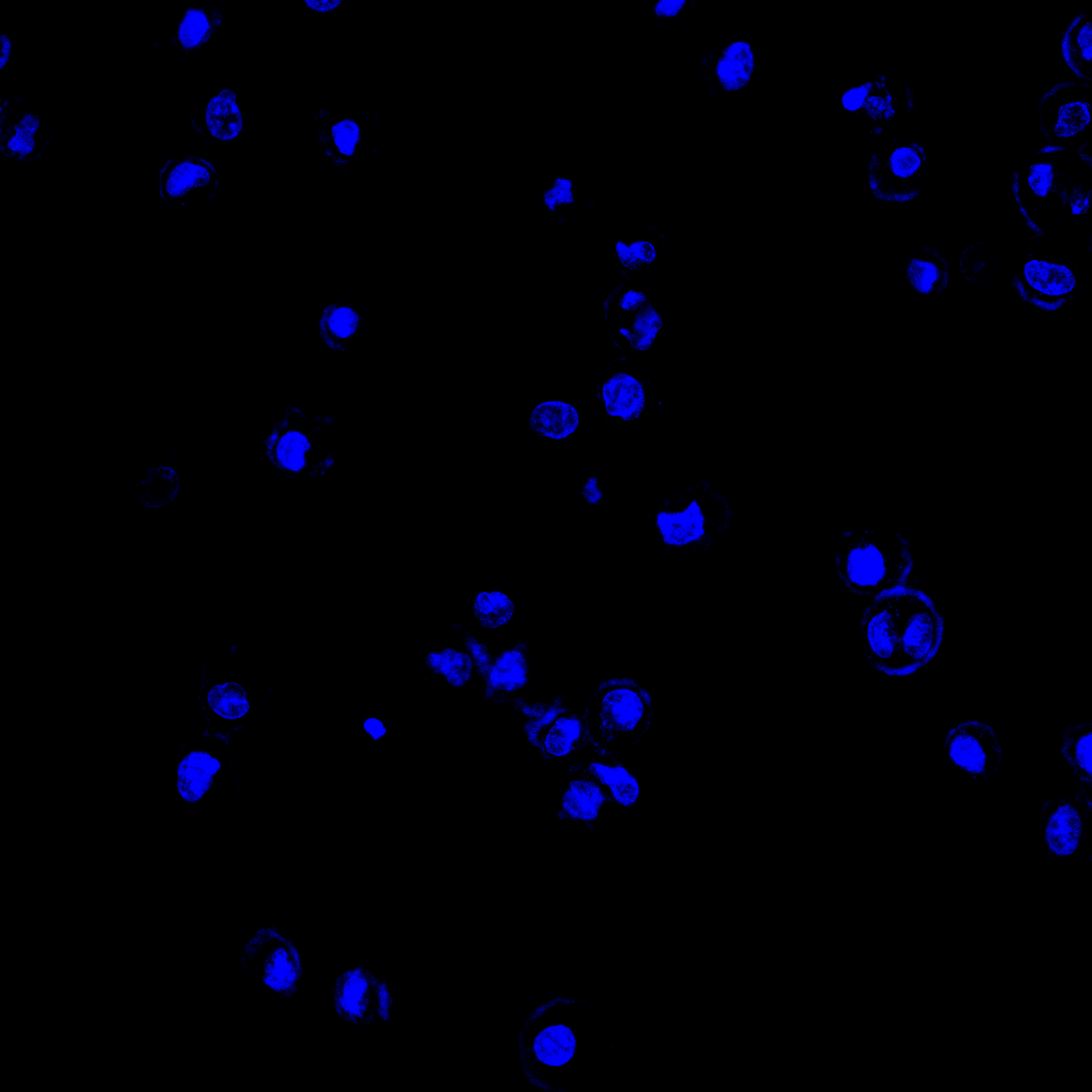

Supplement: Supplementary file 2 [file DataSheet11.ZIP › Fig 6A/NGR1+Compound C/NGR1+Compound C-Hoechst.tif]

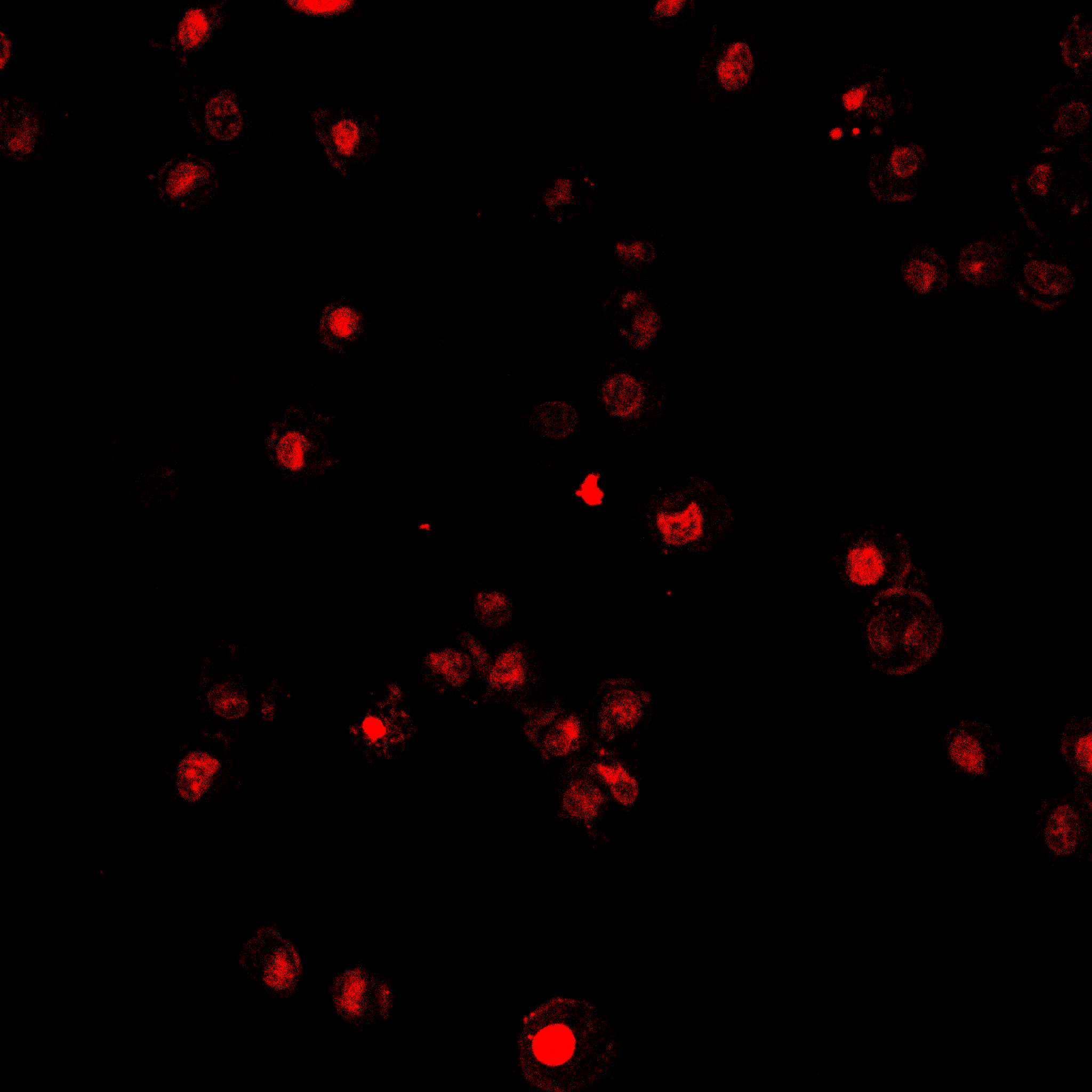

Supplement: Supplementary file 2 [file DataSheet11.ZIP › Fig 6A/NGR1+Compound C/NGR1+Compound C-PI.tif]

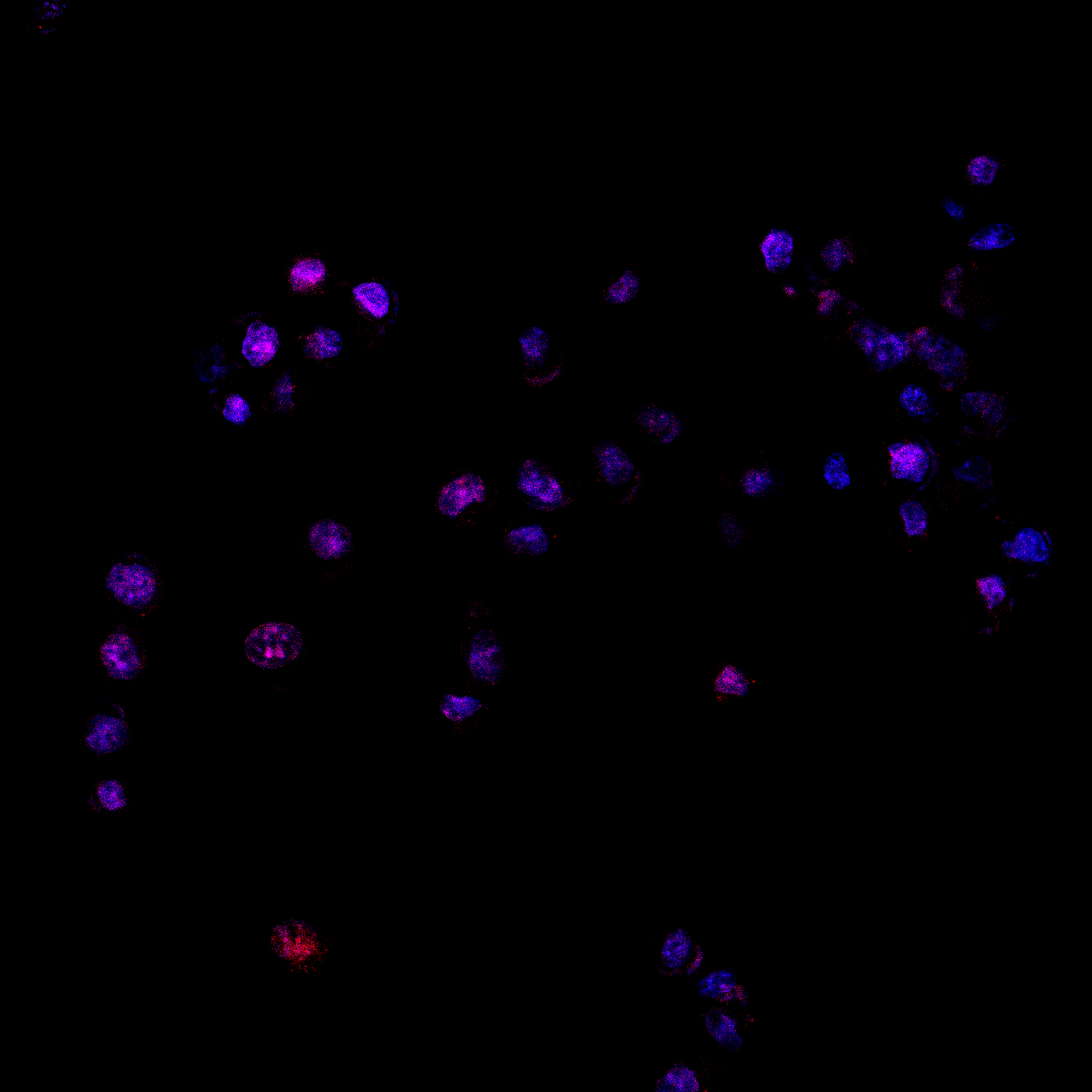

Supplement: Supplementary file 2 [file DataSheet11.ZIP › Fig 6A/NGR1/Merge.tif]

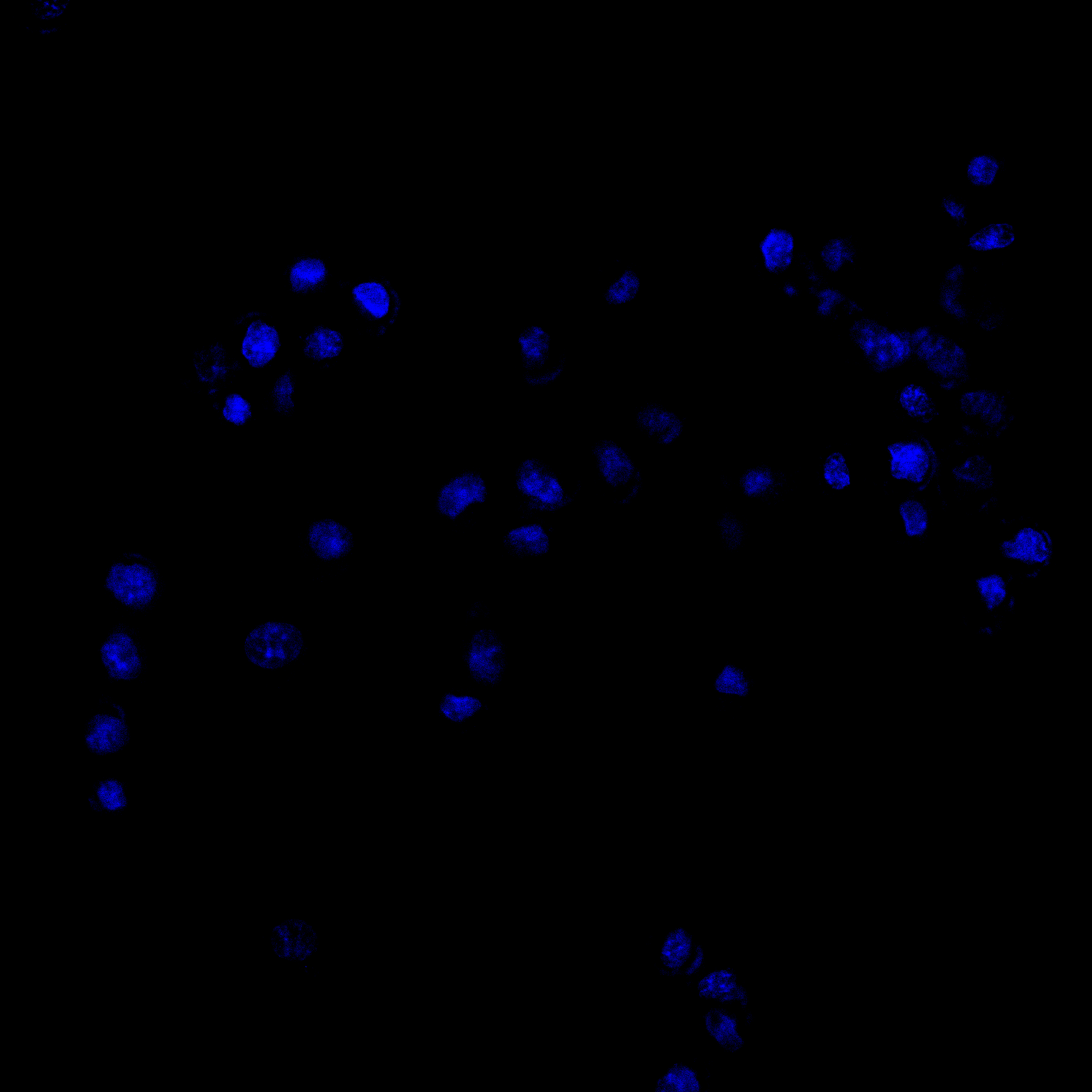

Supplement: Supplementary file 2 [file DataSheet11.ZIP › Fig 6A/NGR1/NGR1-Hoechst.tif]

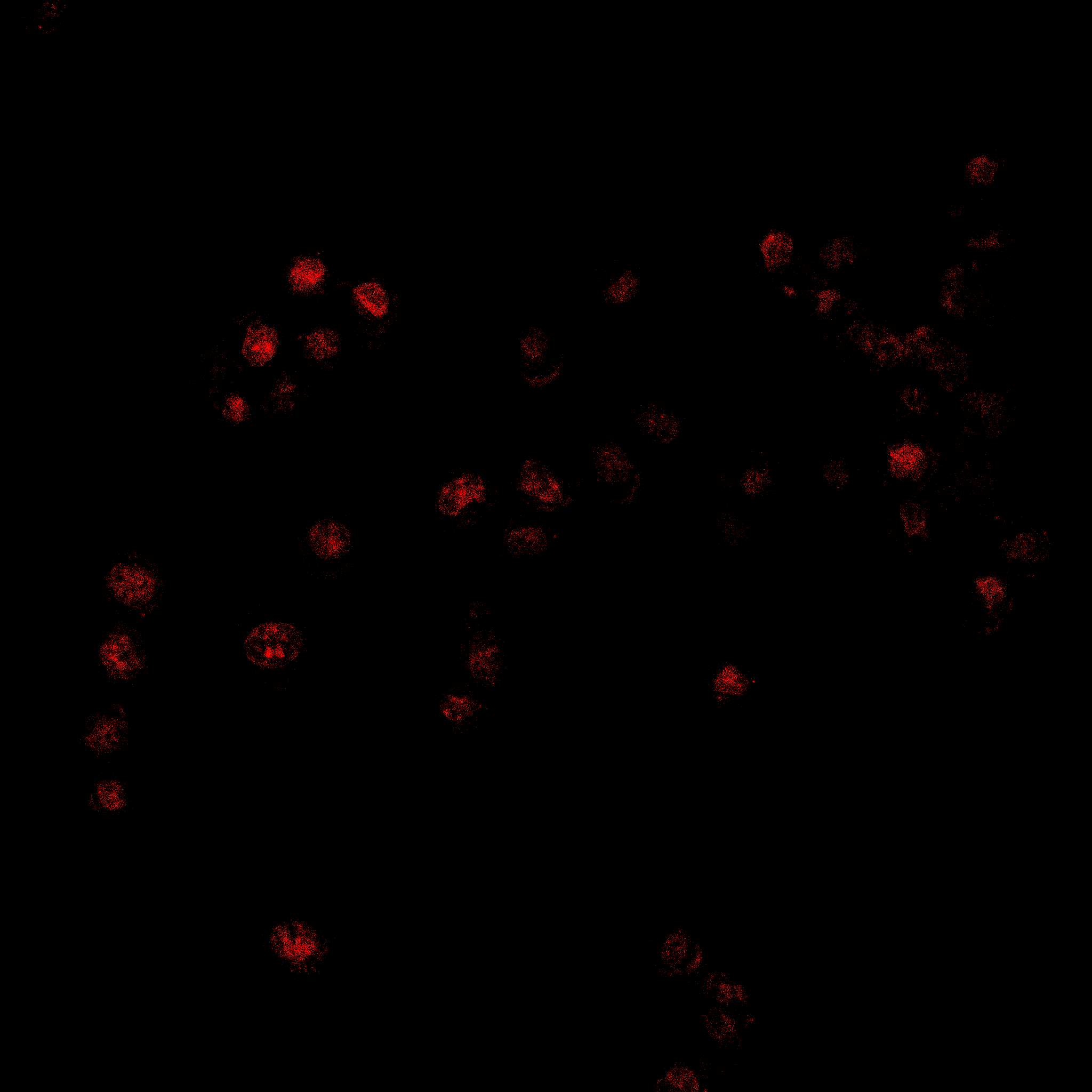

Supplement: Supplementary file 2 [file DataSheet11.ZIP › Fig 6A/NGR1/NGR1-PI.tif]

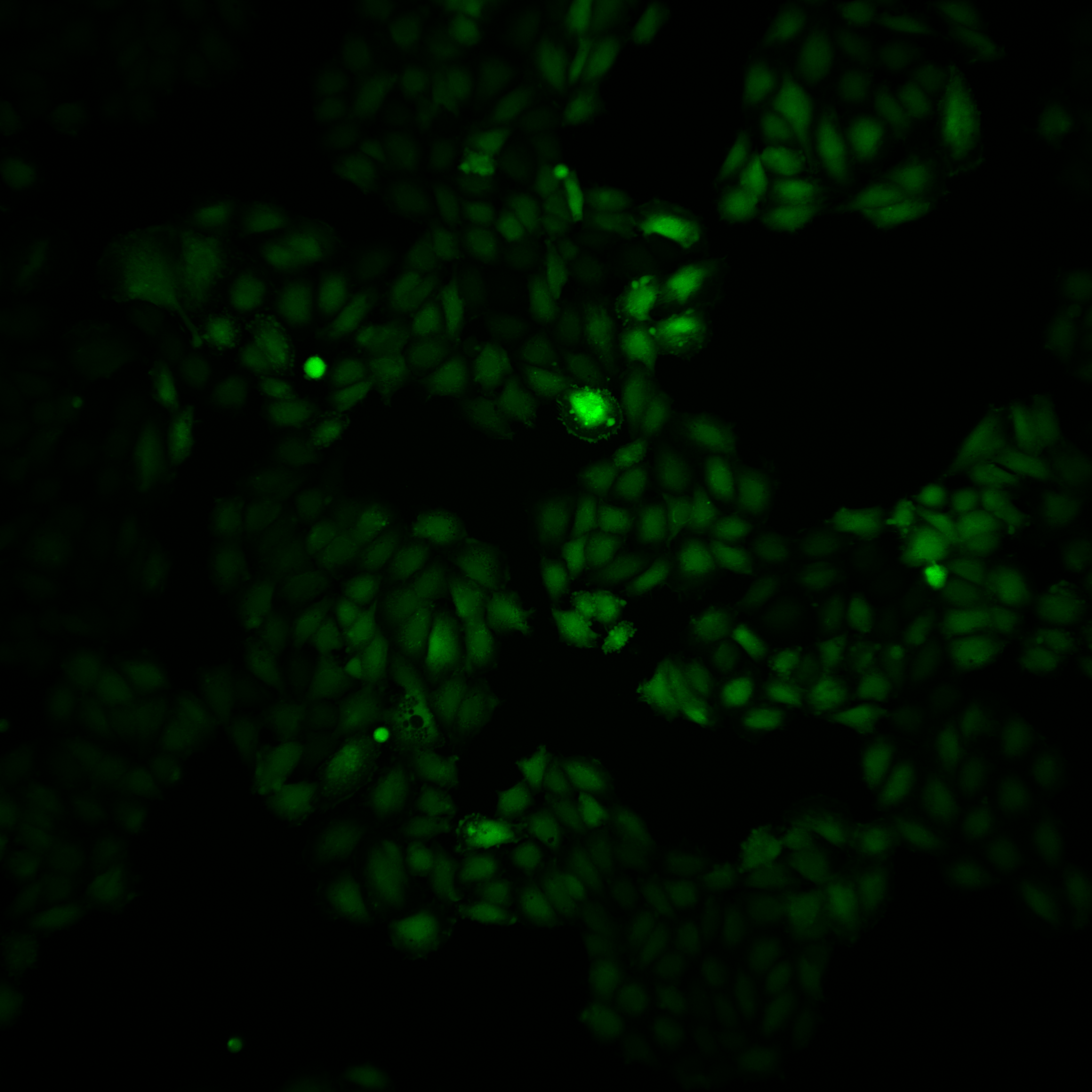

Supplement: Supplementary file 2 [file DataSheet11.ZIP › Fig 6B/Control.tif]

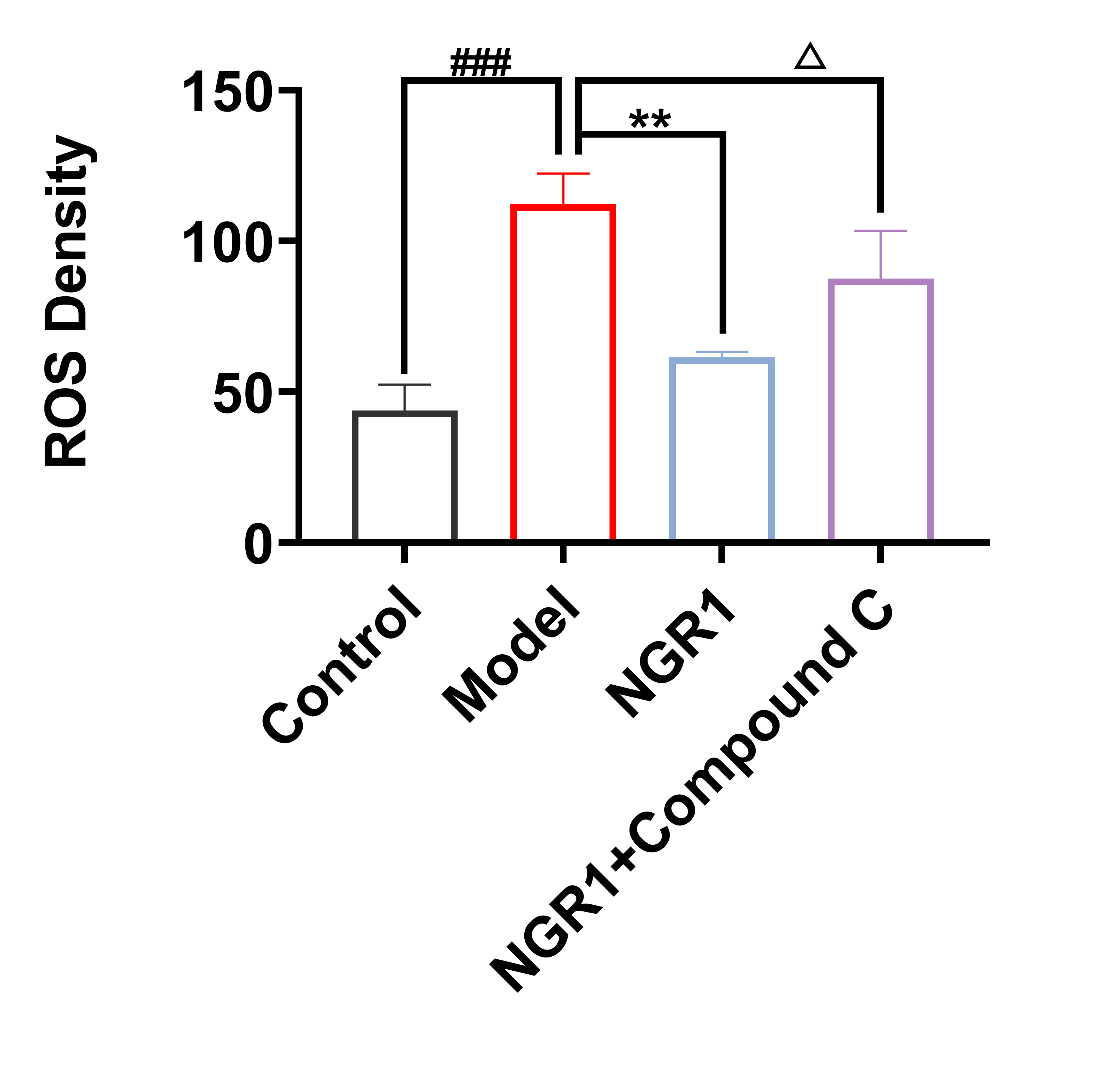

Supplement: Supplementary file 2 [file DataSheet11.ZIP › Fig 6B/Fig 6B.tif]

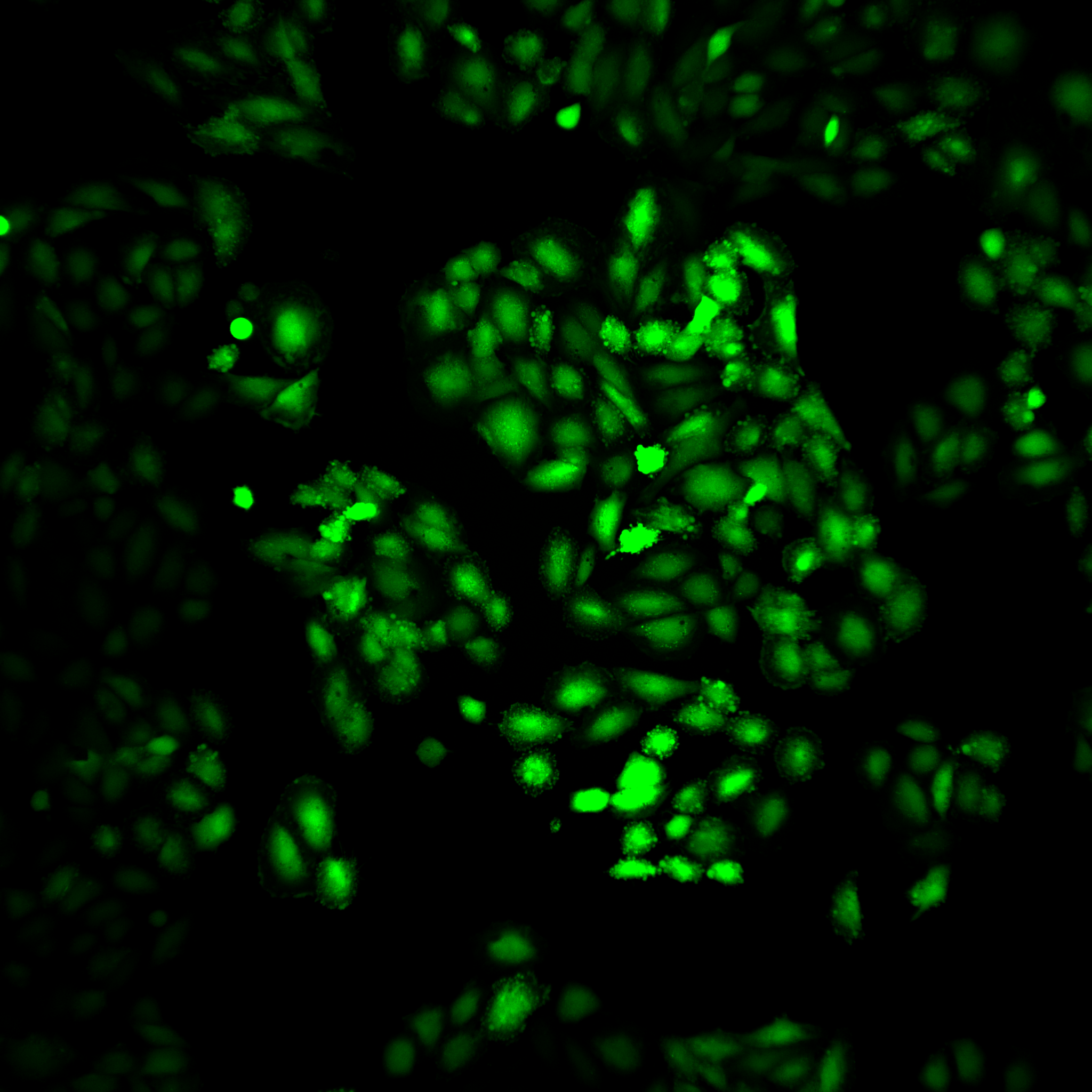

Supplement: Supplementary file 2 [file DataSheet11.ZIP › Fig 6B/Model.tif]

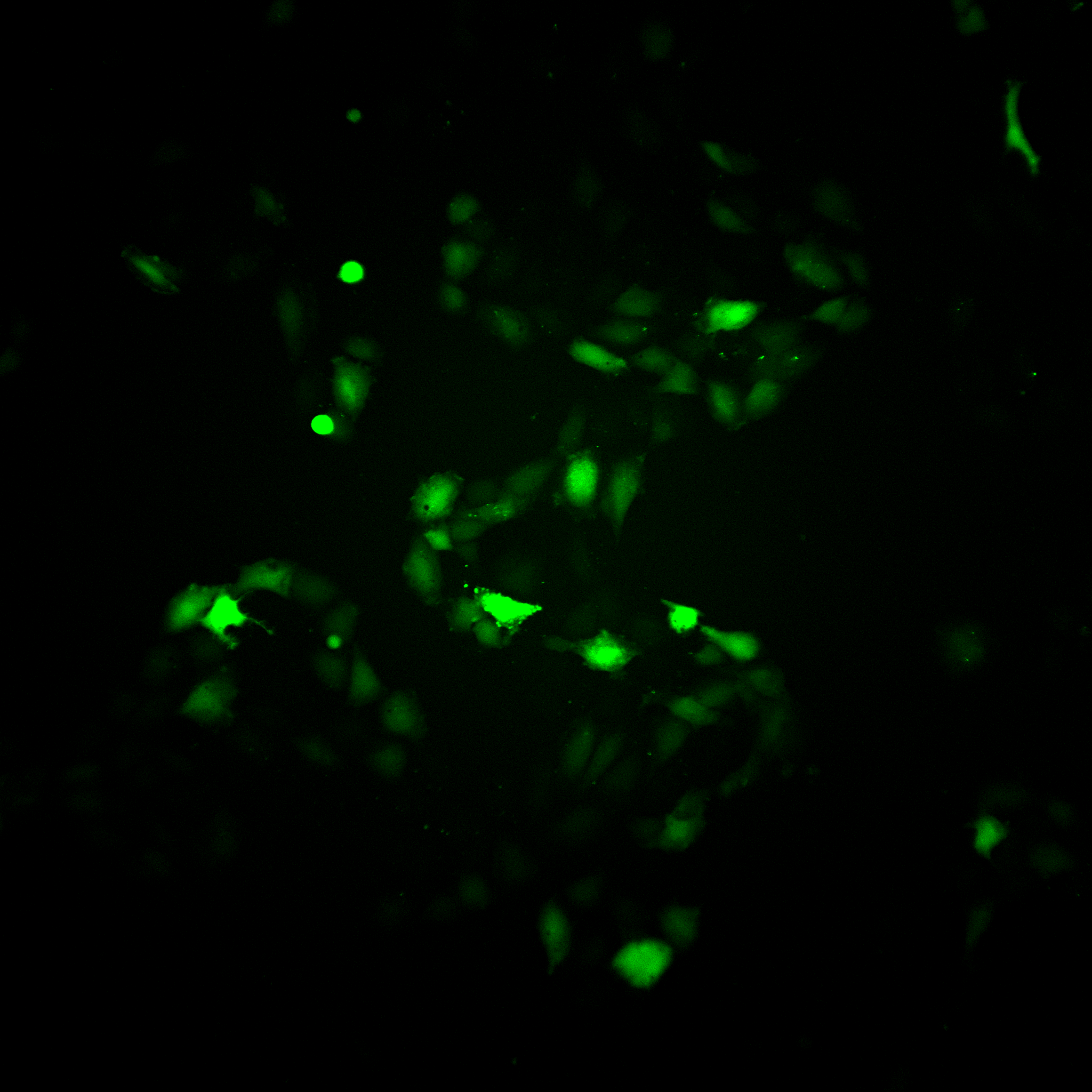

Supplement: Supplementary file 2 [file DataSheet11.ZIP › Fig 6B/NGR1+Compound C.tif]

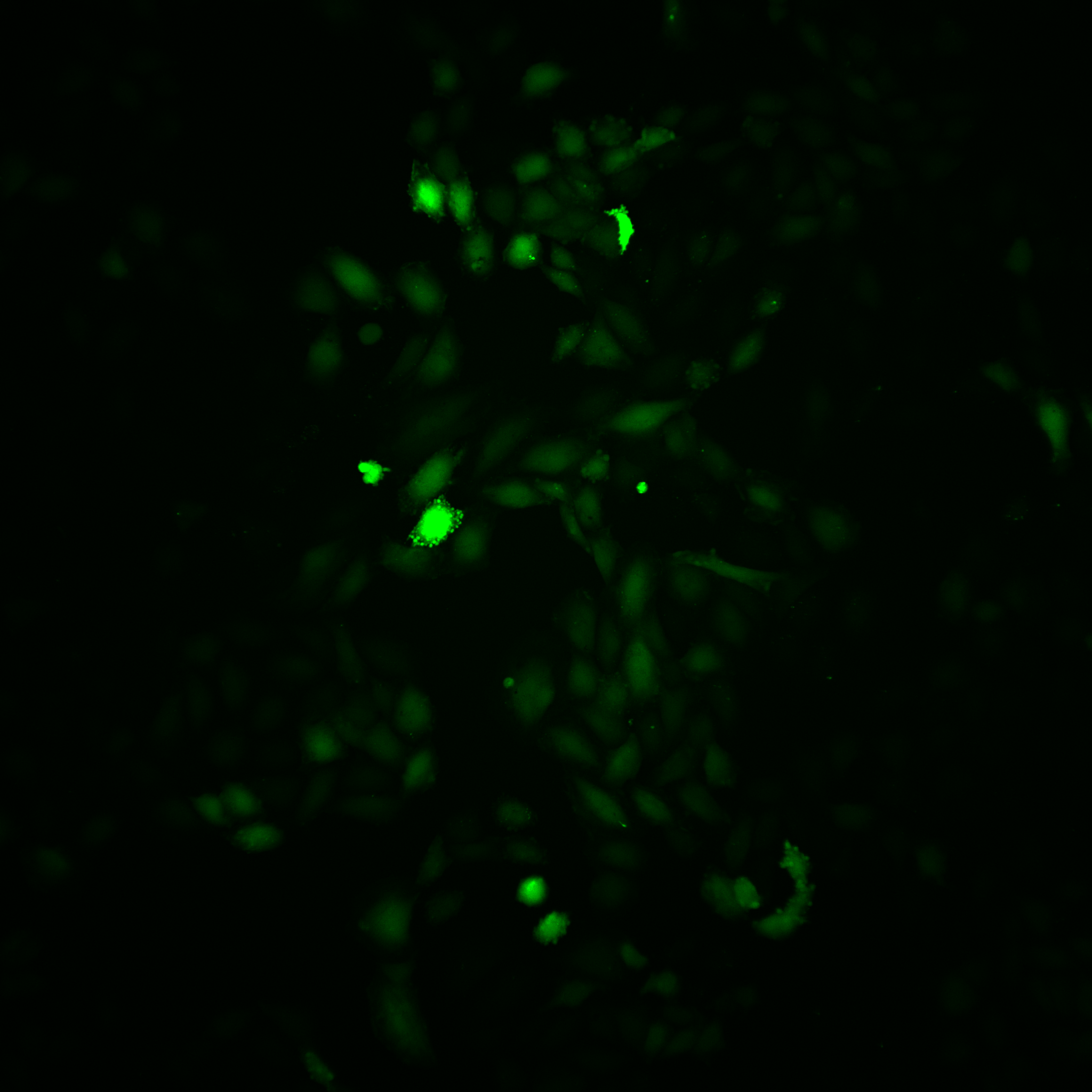

Supplement: Supplementary file 2 [file DataSheet11.ZIP › Fig 6B/NGR1.tif]

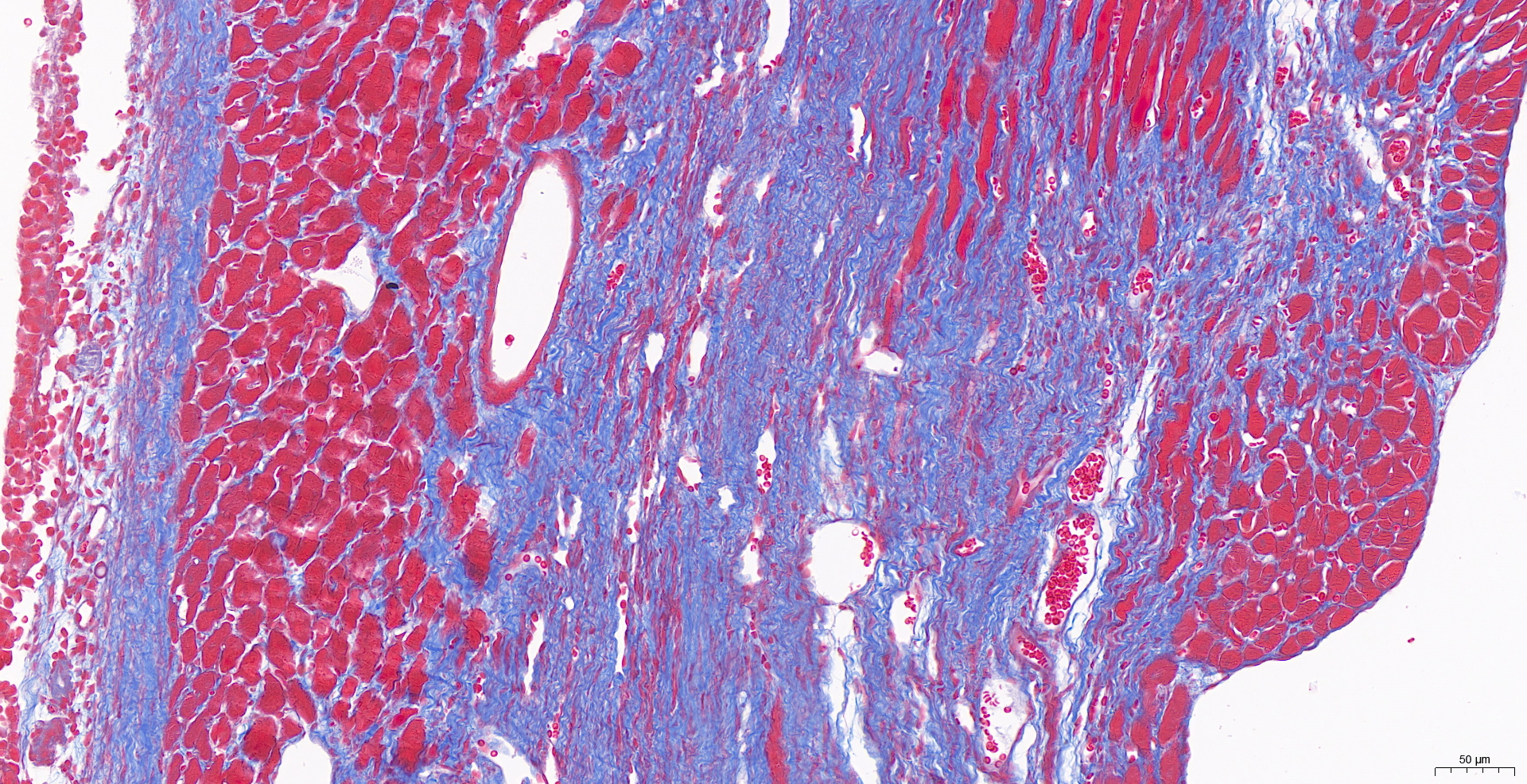

Supplement: Supplementary file 3 [file DataSheet8.ZIP › Fig 1D/20x/Fig 1D Model masson_20.0x.jpg]

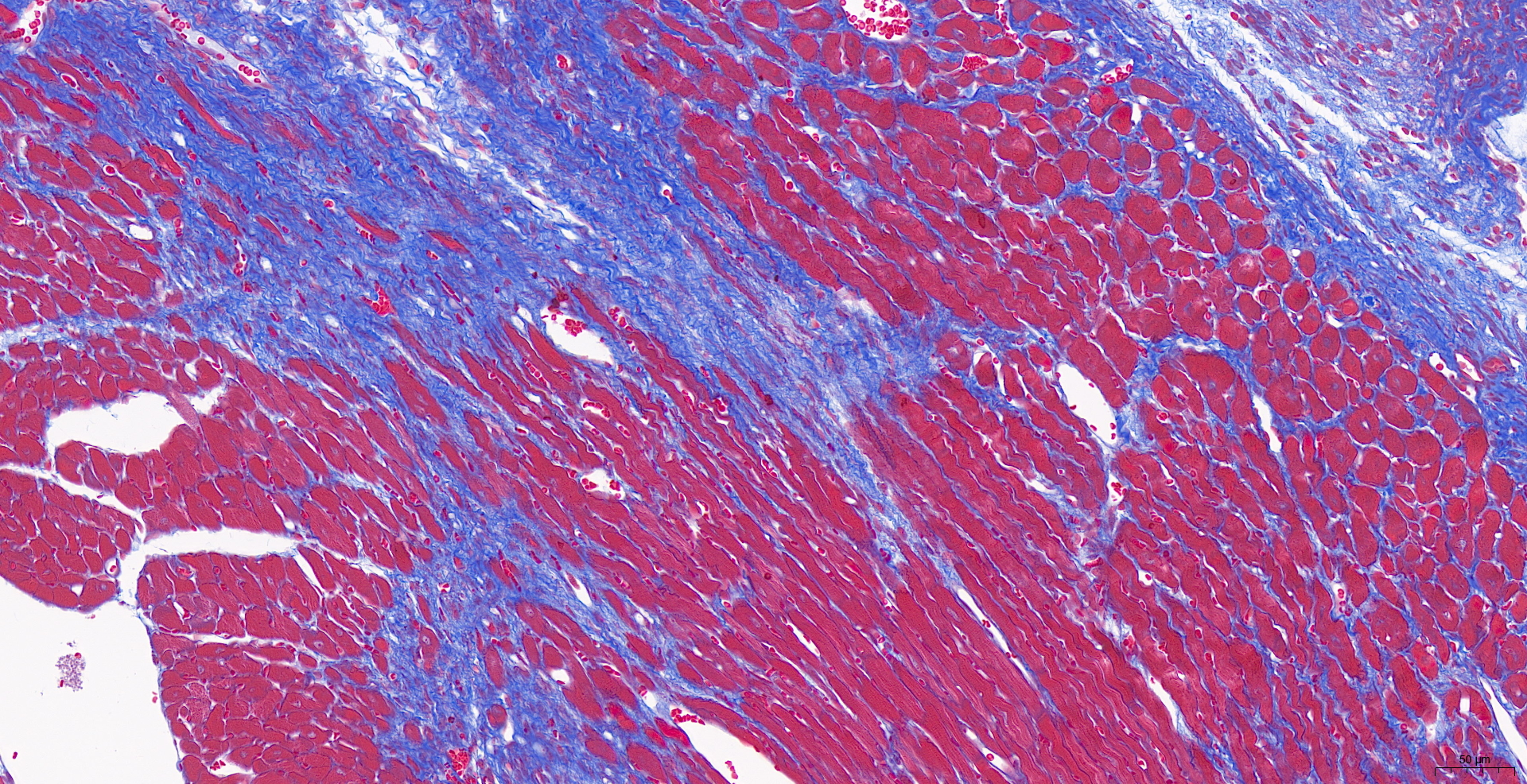

Supplement: Supplementary file 3 [file DataSheet8.ZIP › Fig 1D/20x/Fig 1D NGR1 masson_20.0x.bmp]

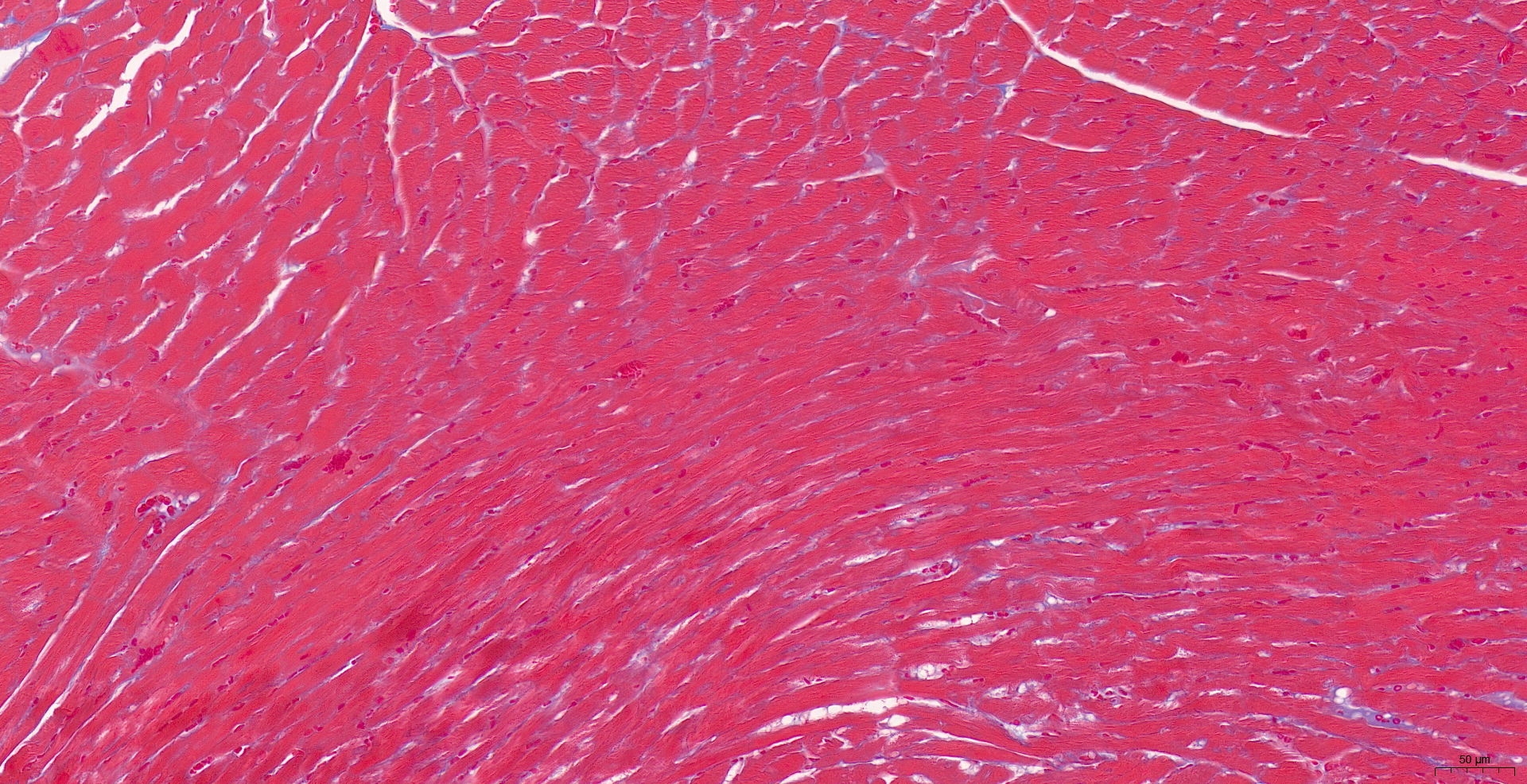

Supplement: Supplementary file 3 [file DataSheet8.ZIP › Fig 1D/20x/Fig 1D Sham masson_20.0x.jpg]

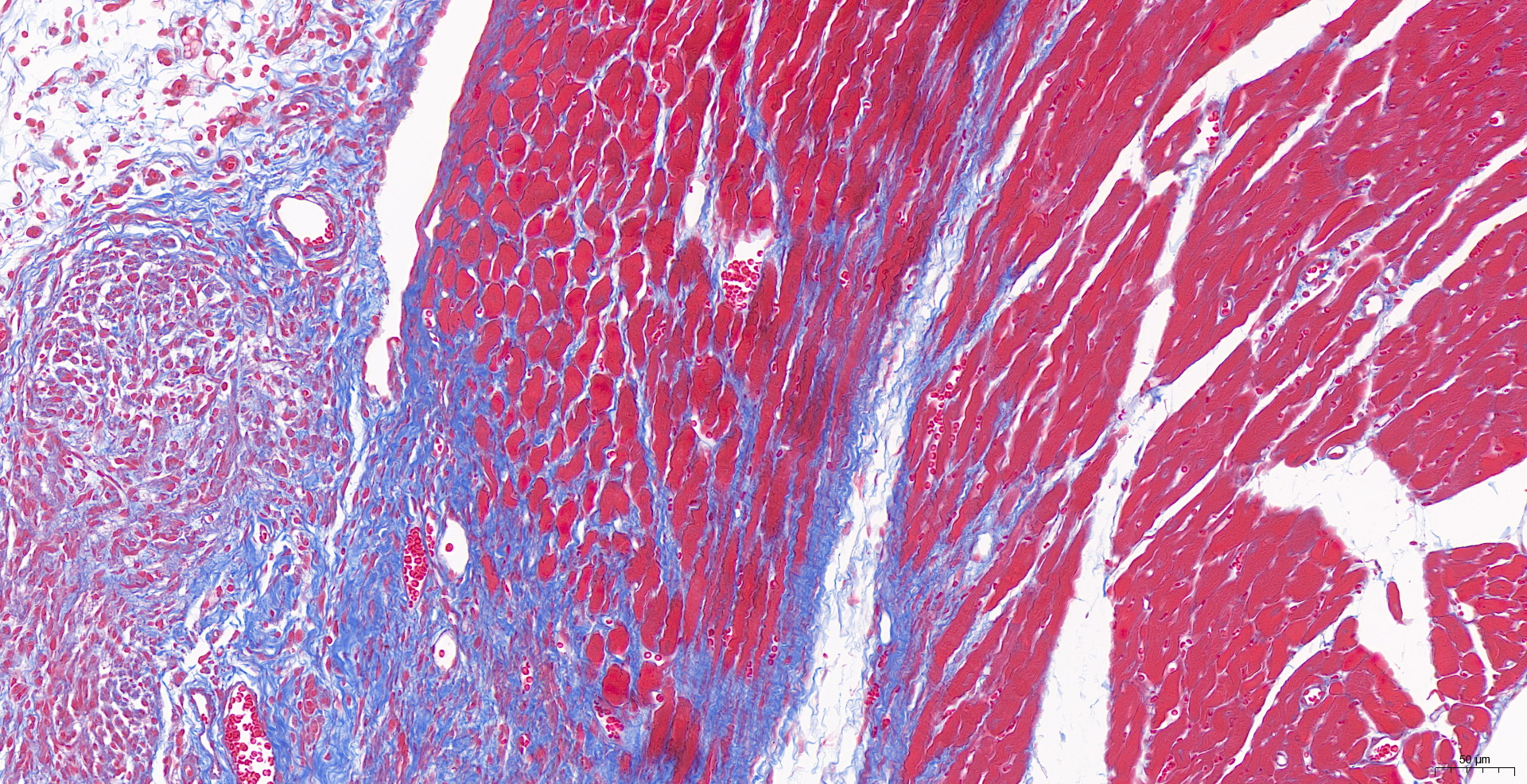

Supplement: Supplementary file 3 [file DataSheet8.ZIP › Fig 1D/20x/Fig 1D Simvastatin masson_20.0x.jpg]

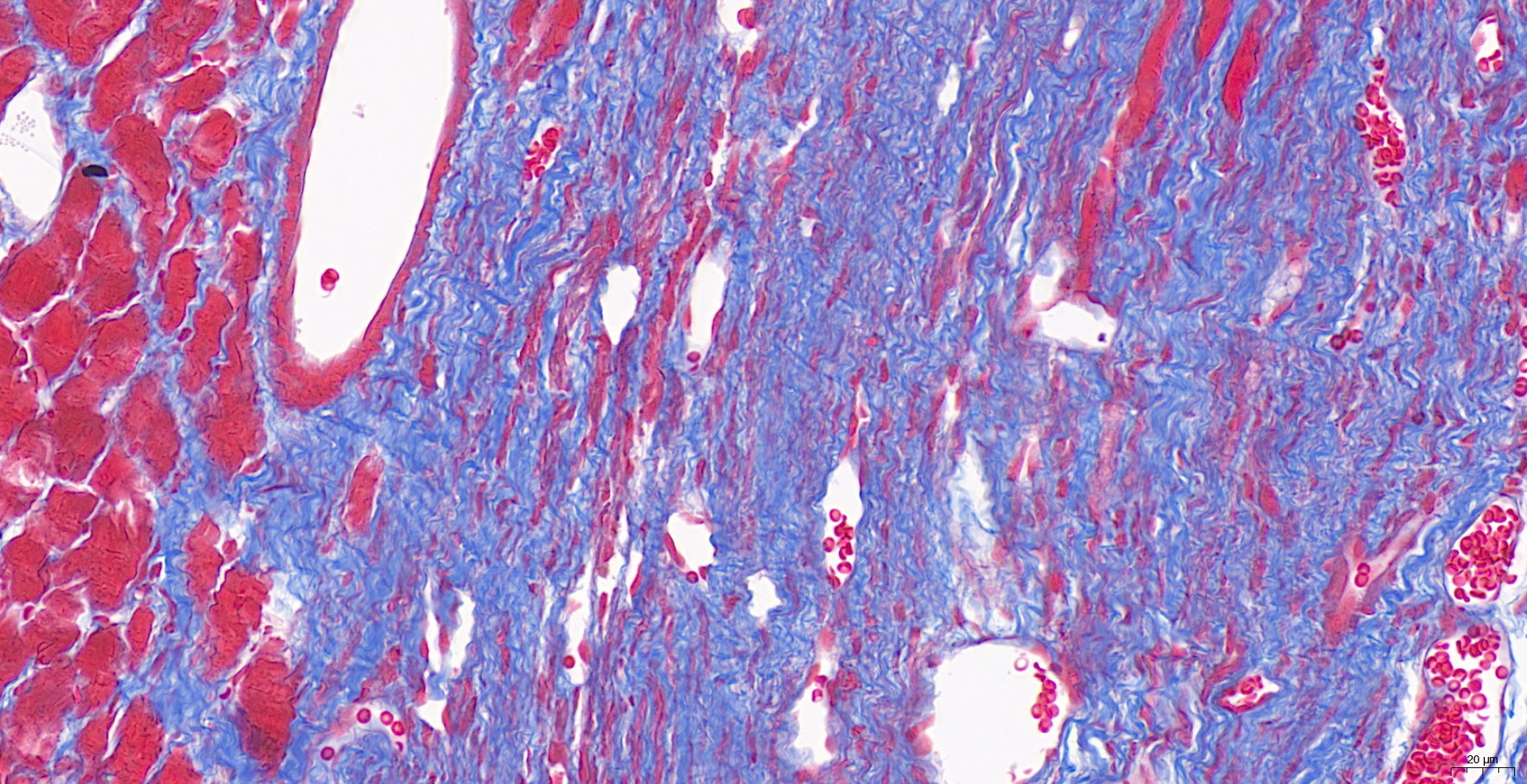

Supplement: Supplementary file 3 [file DataSheet8.ZIP › Fig 1D/40x/Fig 1D Model masson_40.0x.jpg]

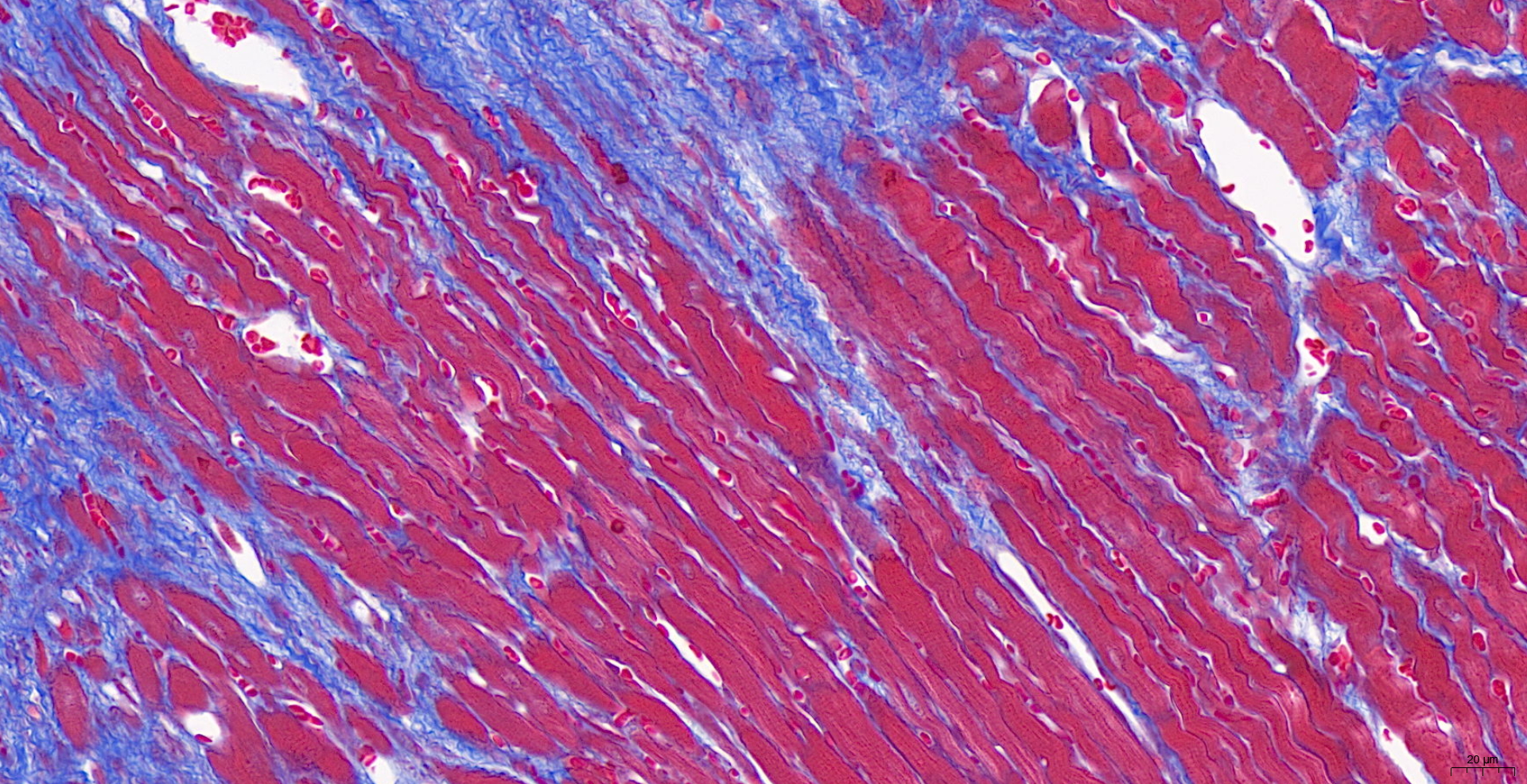

Supplement: Supplementary file 3 [file DataSheet8.ZIP › Fig 1D/40x/Fig 1D NGR1 masson_40.0x.jpg]

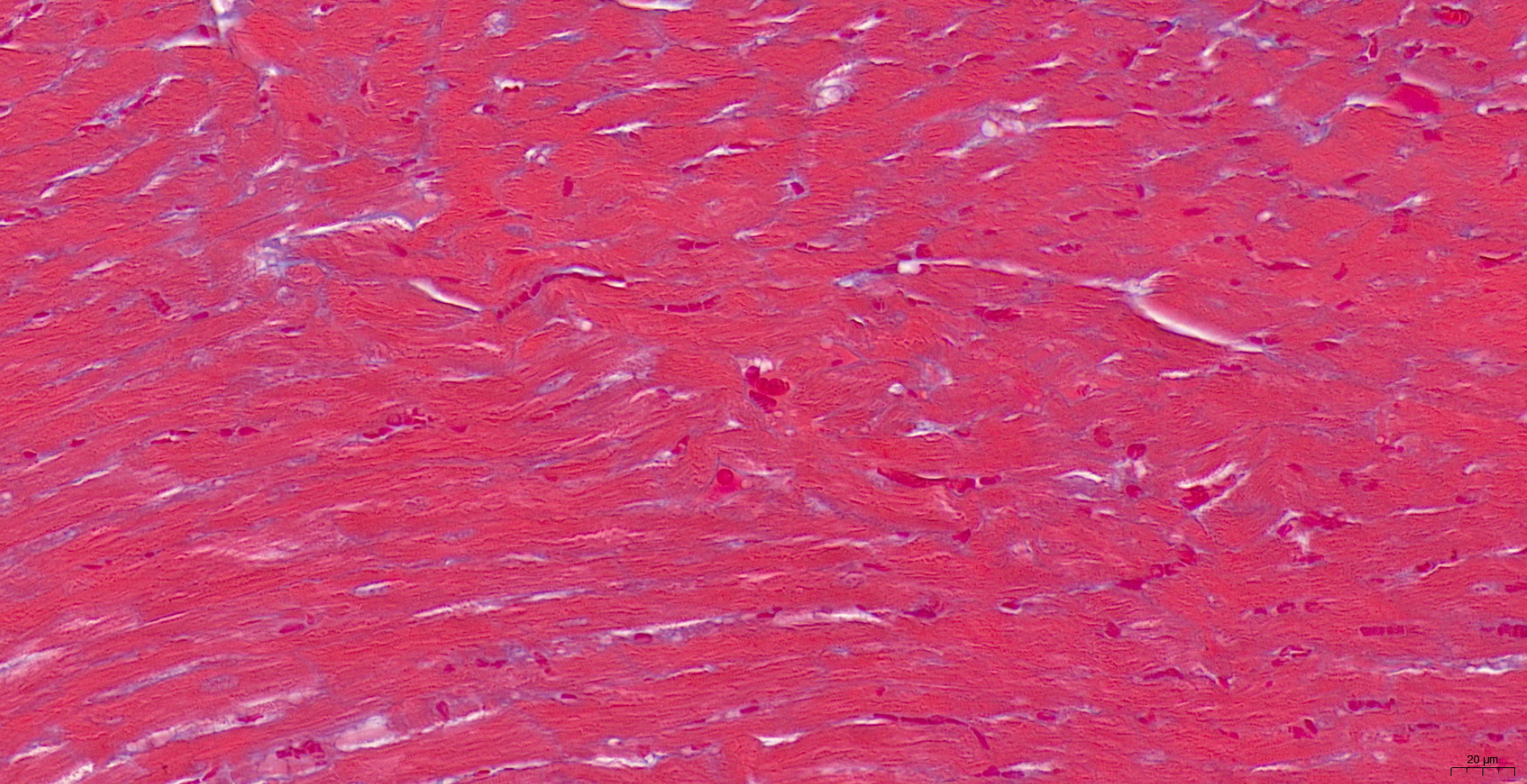

Supplement: Supplementary file 3 [file DataSheet8.ZIP › Fig 1D/40x/Fig 1D Sham masson_40.0x.jpg]

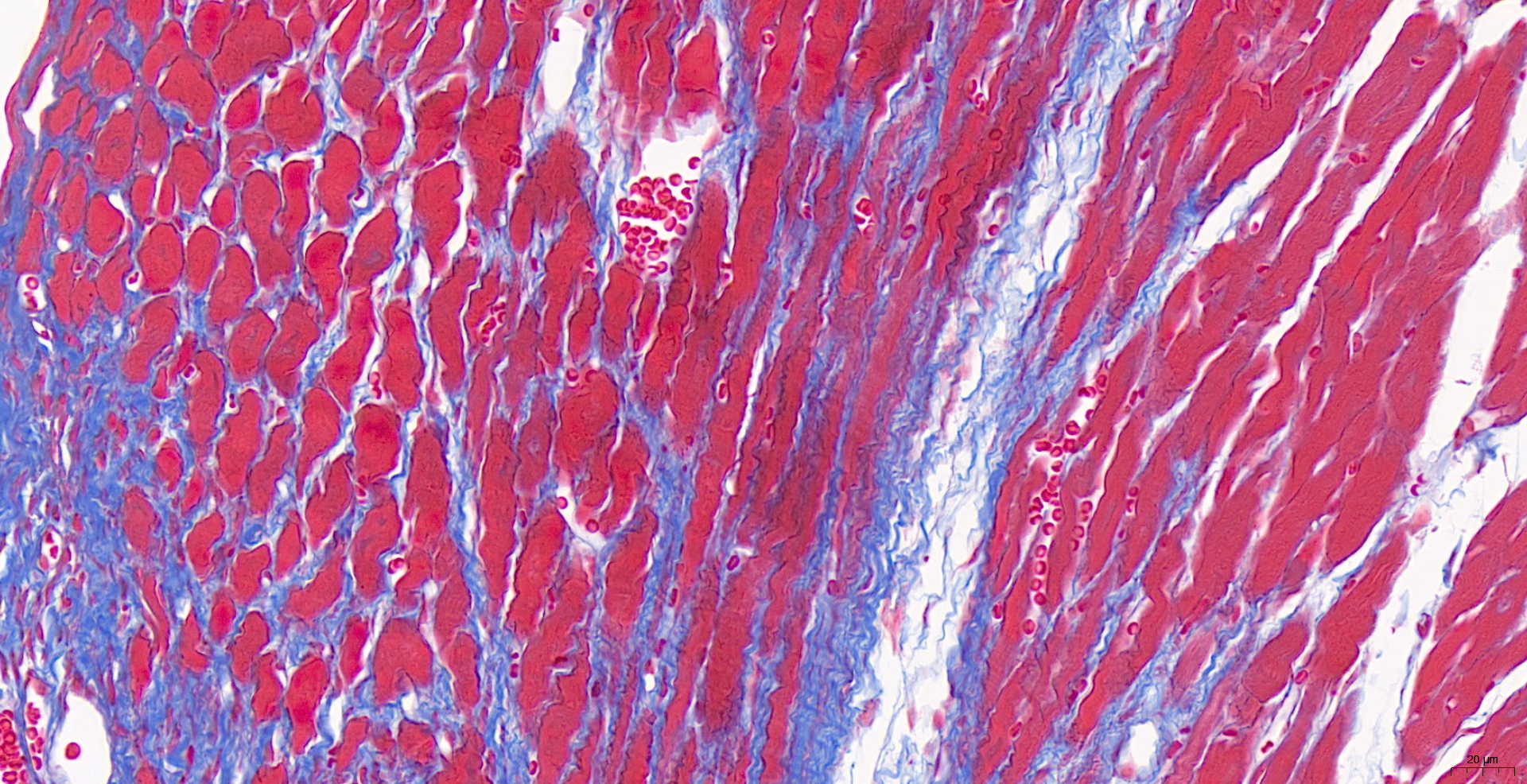

Supplement: Supplementary file 3 [file DataSheet8.ZIP › Fig 1D/40x/Fig 1D Simvastatin masson_40.0x.jpg]

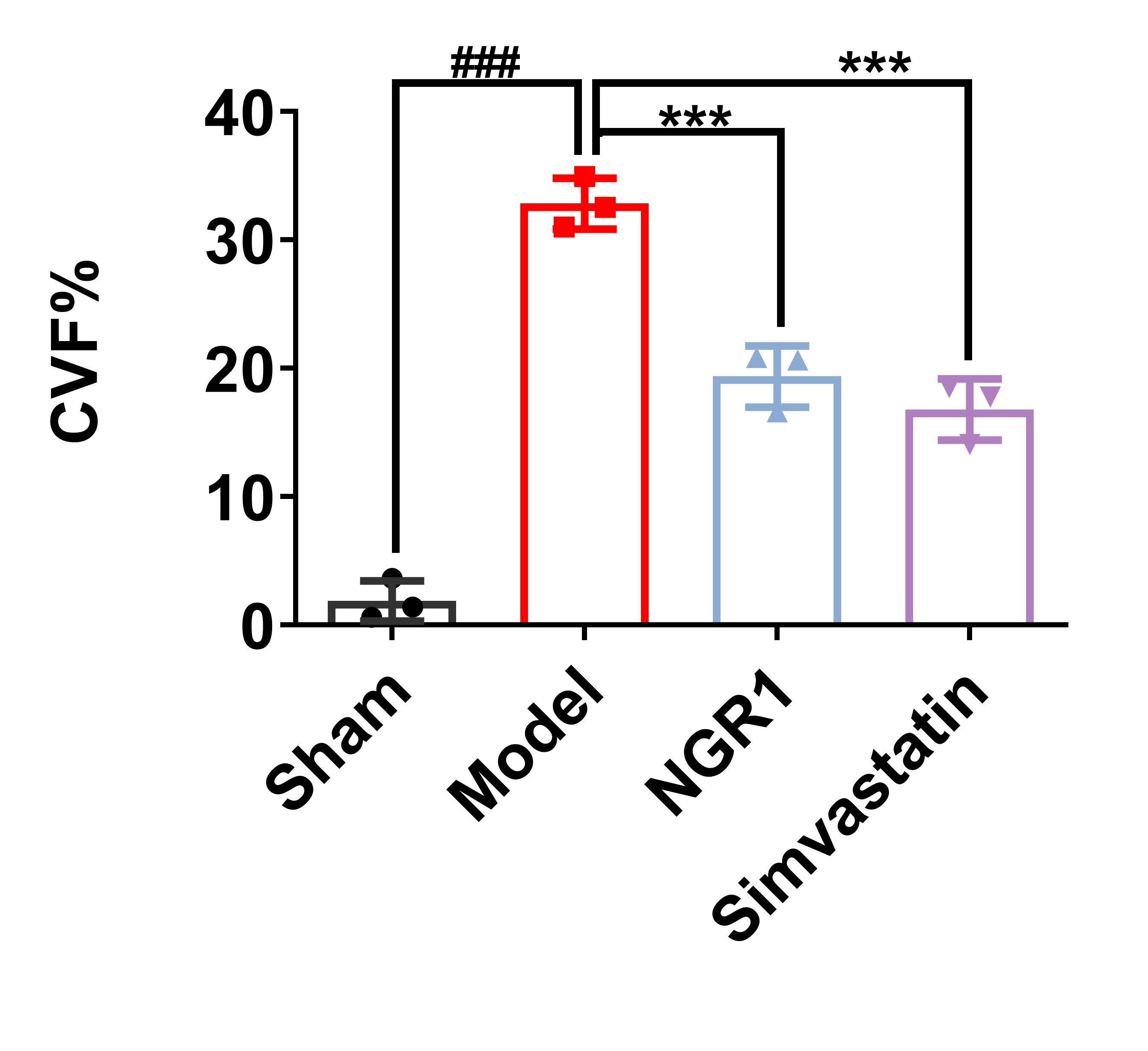

Supplement: Supplementary file 3 [file DataSheet8.ZIP › Fig 1D/CVF.tif]

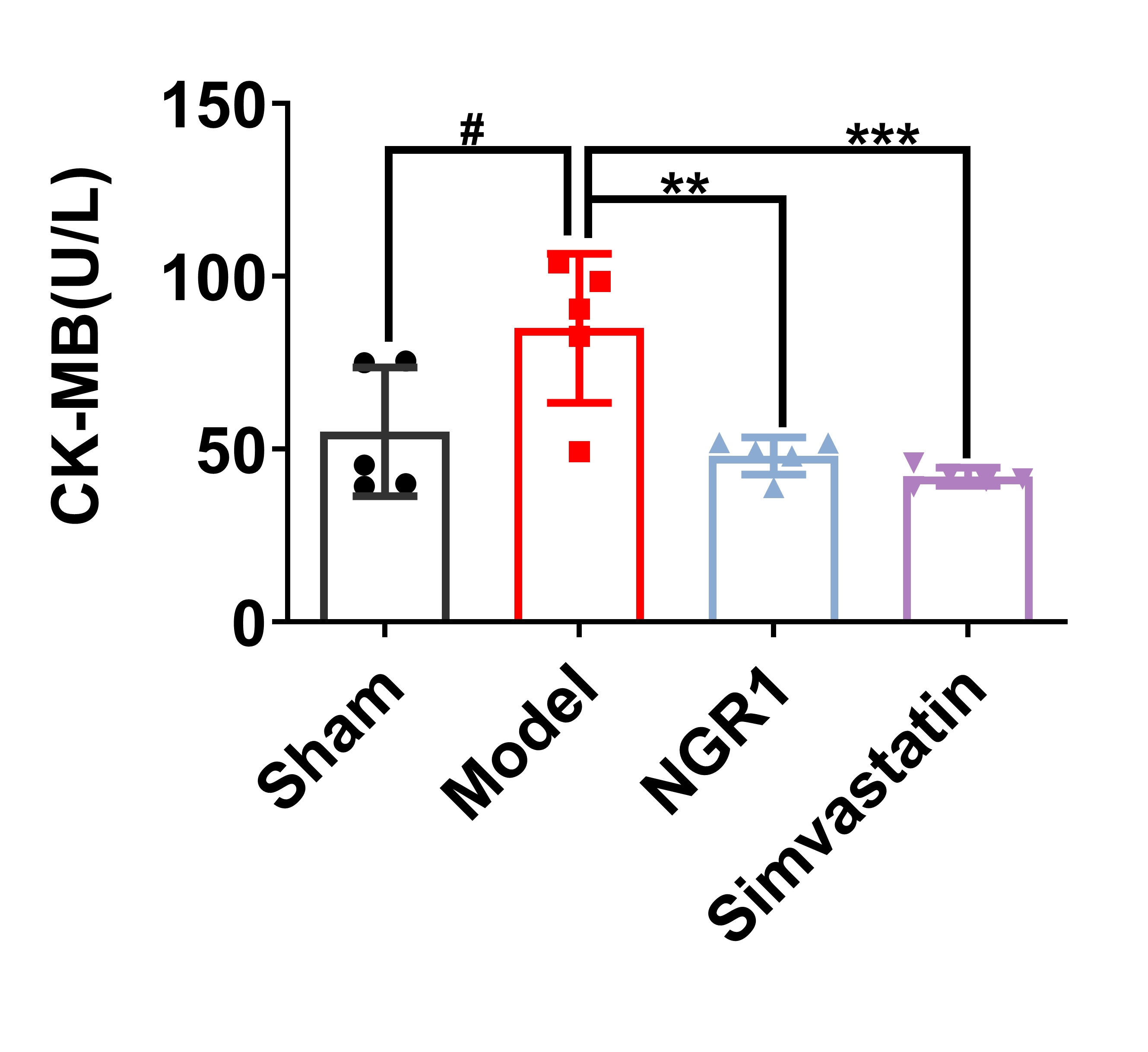

Supplement: Supplementary file 3 [file DataSheet8.ZIP › Fig 1E-F/CK-MB.tif]

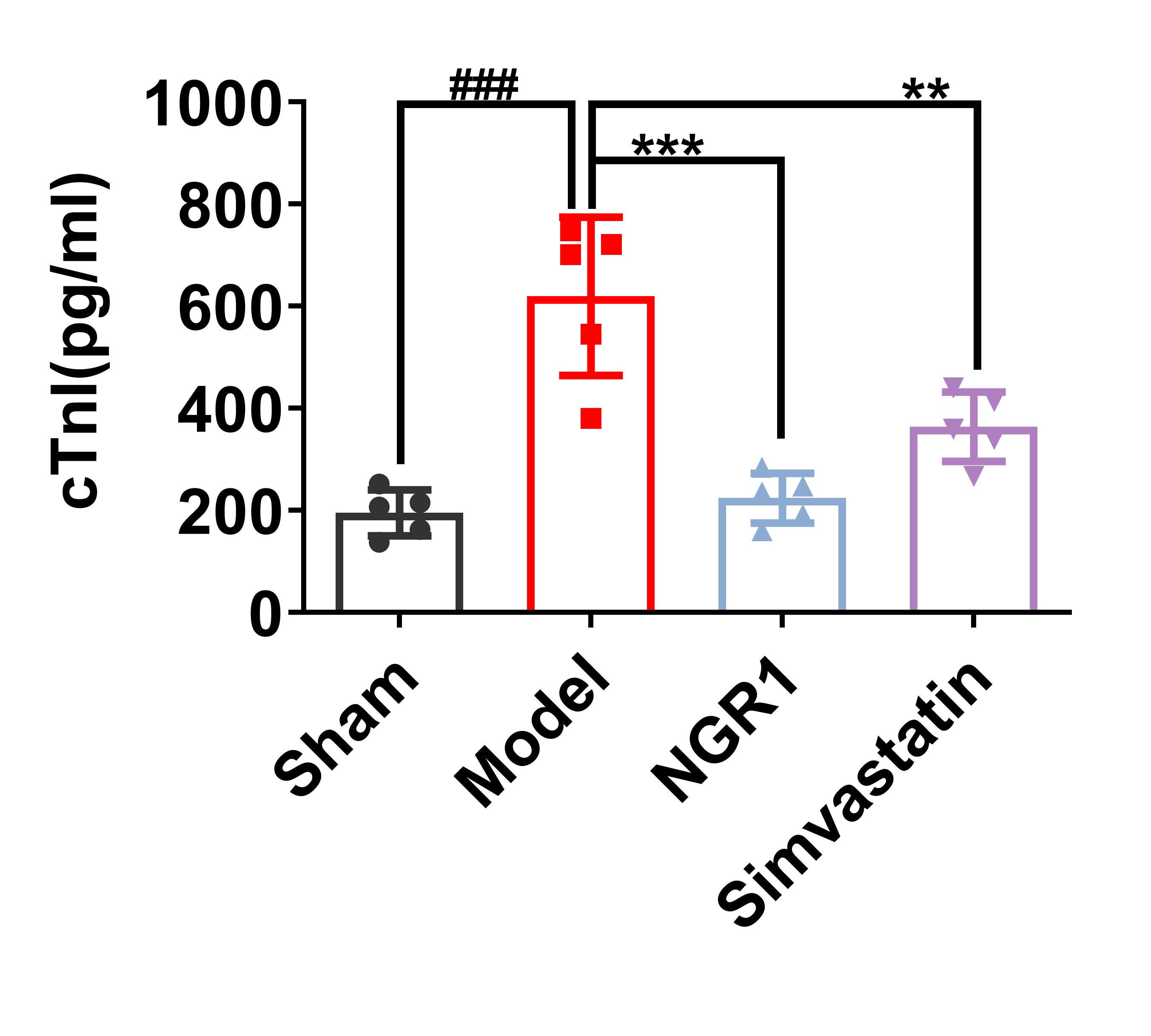

Supplement: Supplementary file 3 [file DataSheet8.ZIP › Fig 1E-F/cTnI.tif]

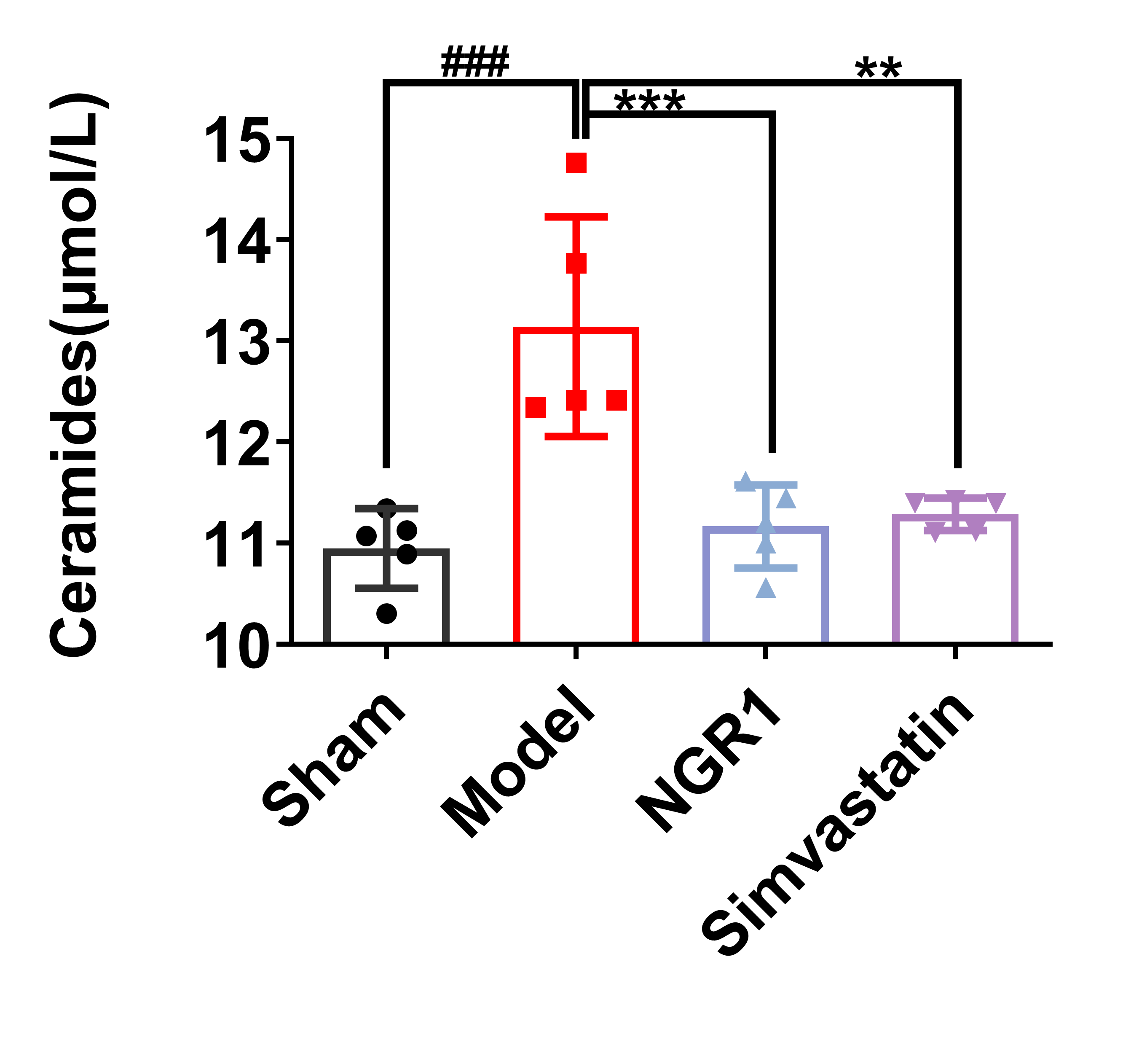

Supplement: Supplementary file 4 [file DataSheet9.ZIP › Figure 2/Ceramide.tif]

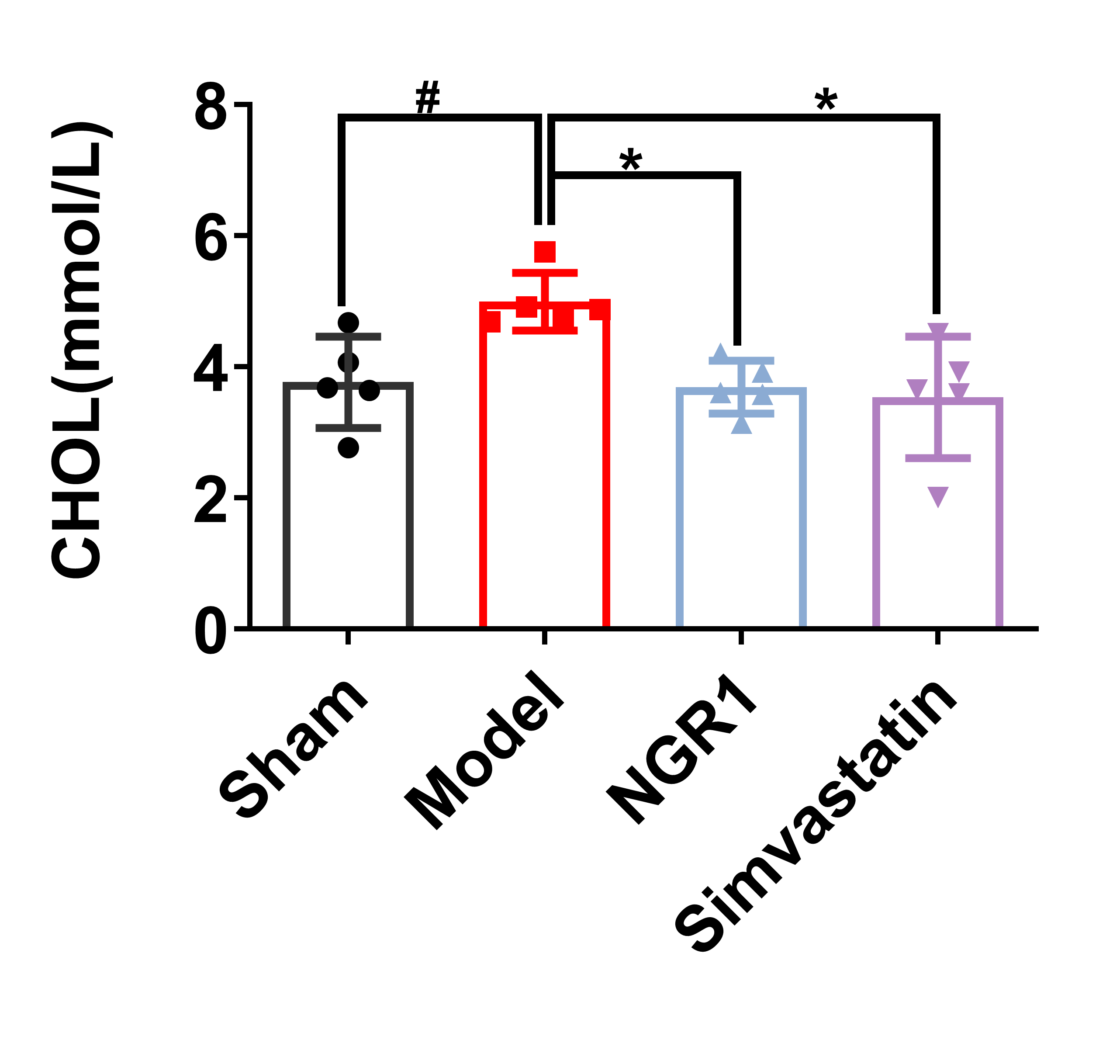

Supplement: Supplementary file 4 [file DataSheet9.ZIP › Figure 2/CHOL.tif]

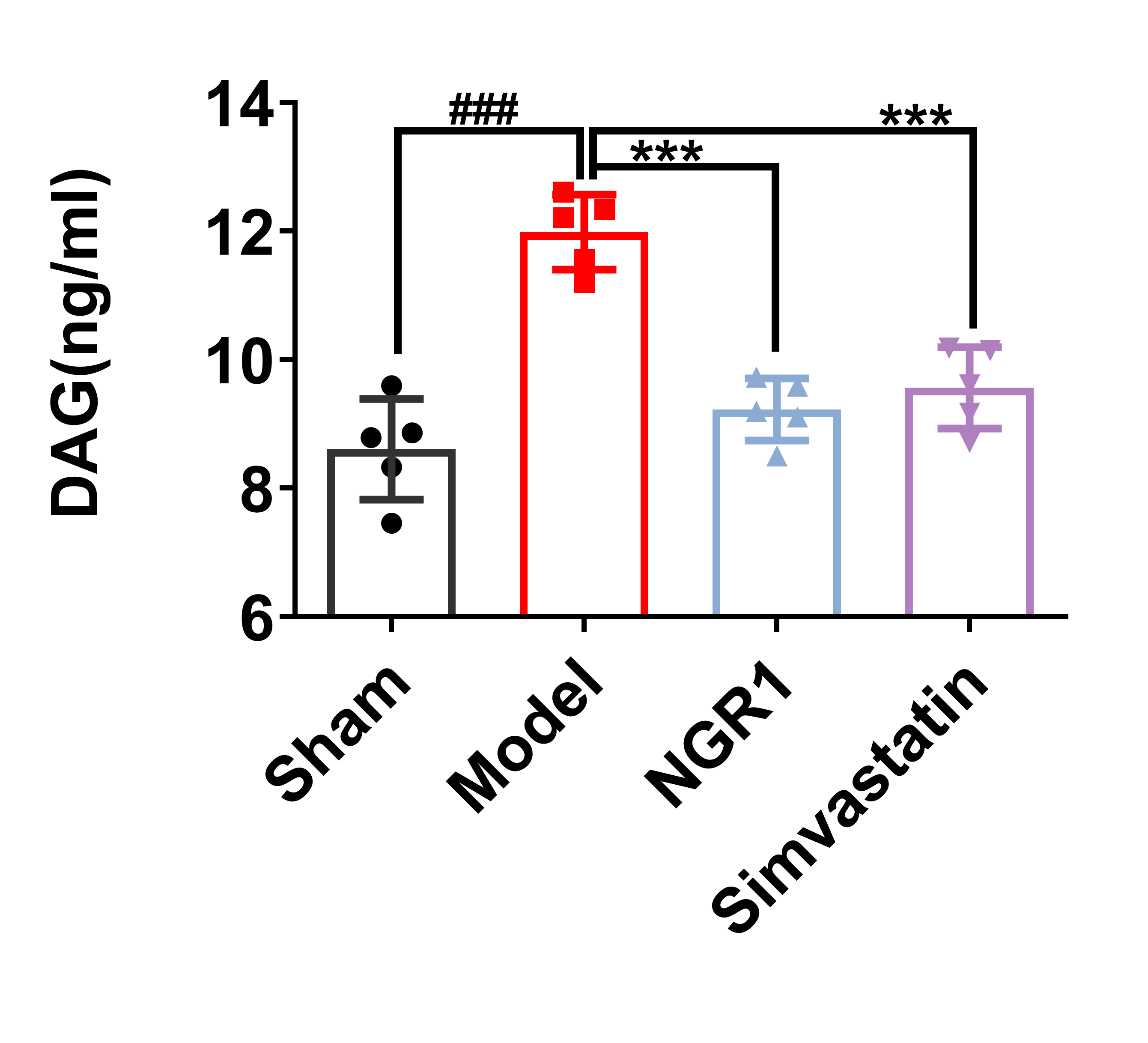

Supplement: Supplementary file 4 [file DataSheet9.ZIP › Figure 2/DAG.tif]

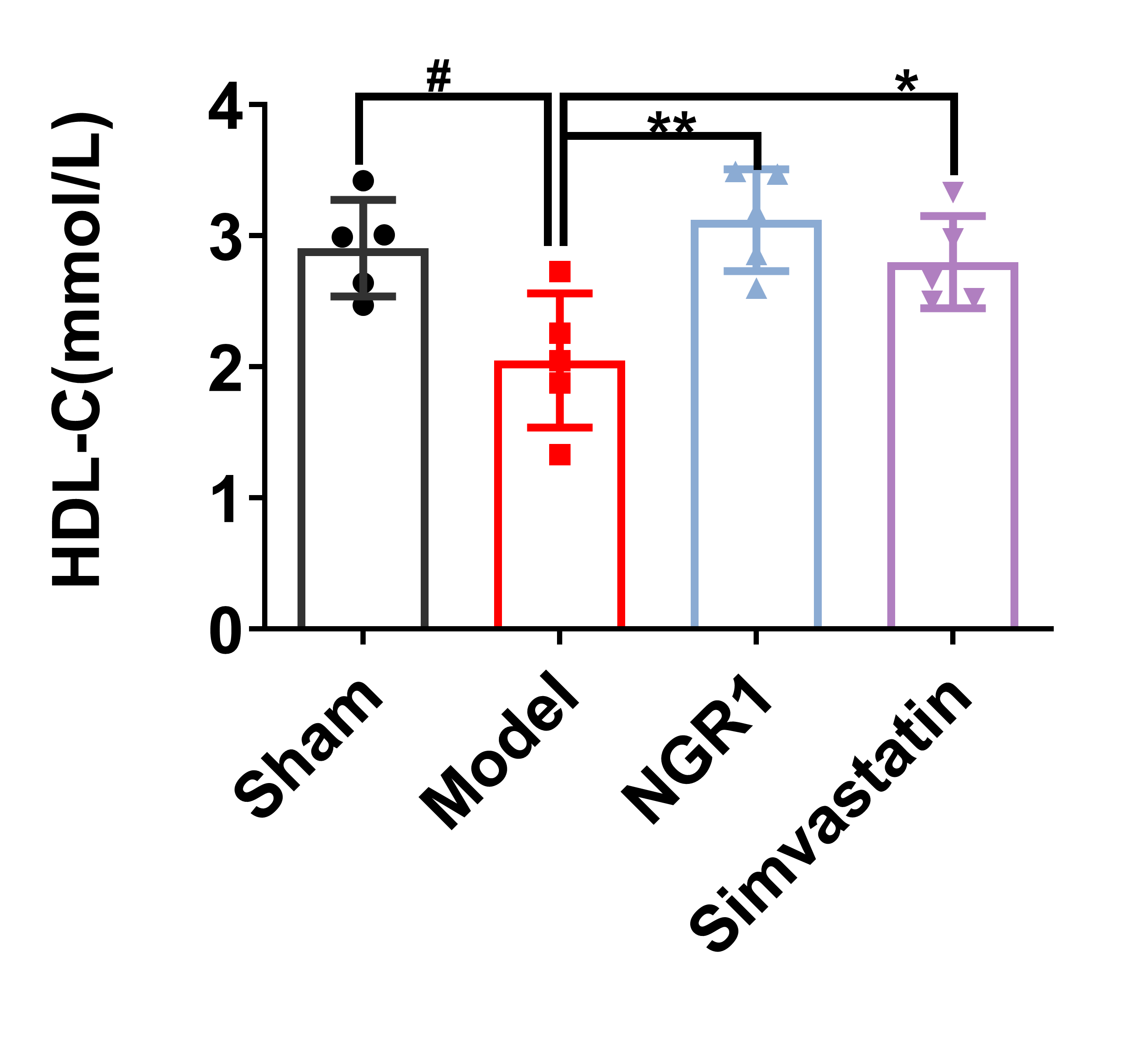

Supplement: Supplementary file 4 [file DataSheet9.ZIP › Figure 2/HDL-C.tif]

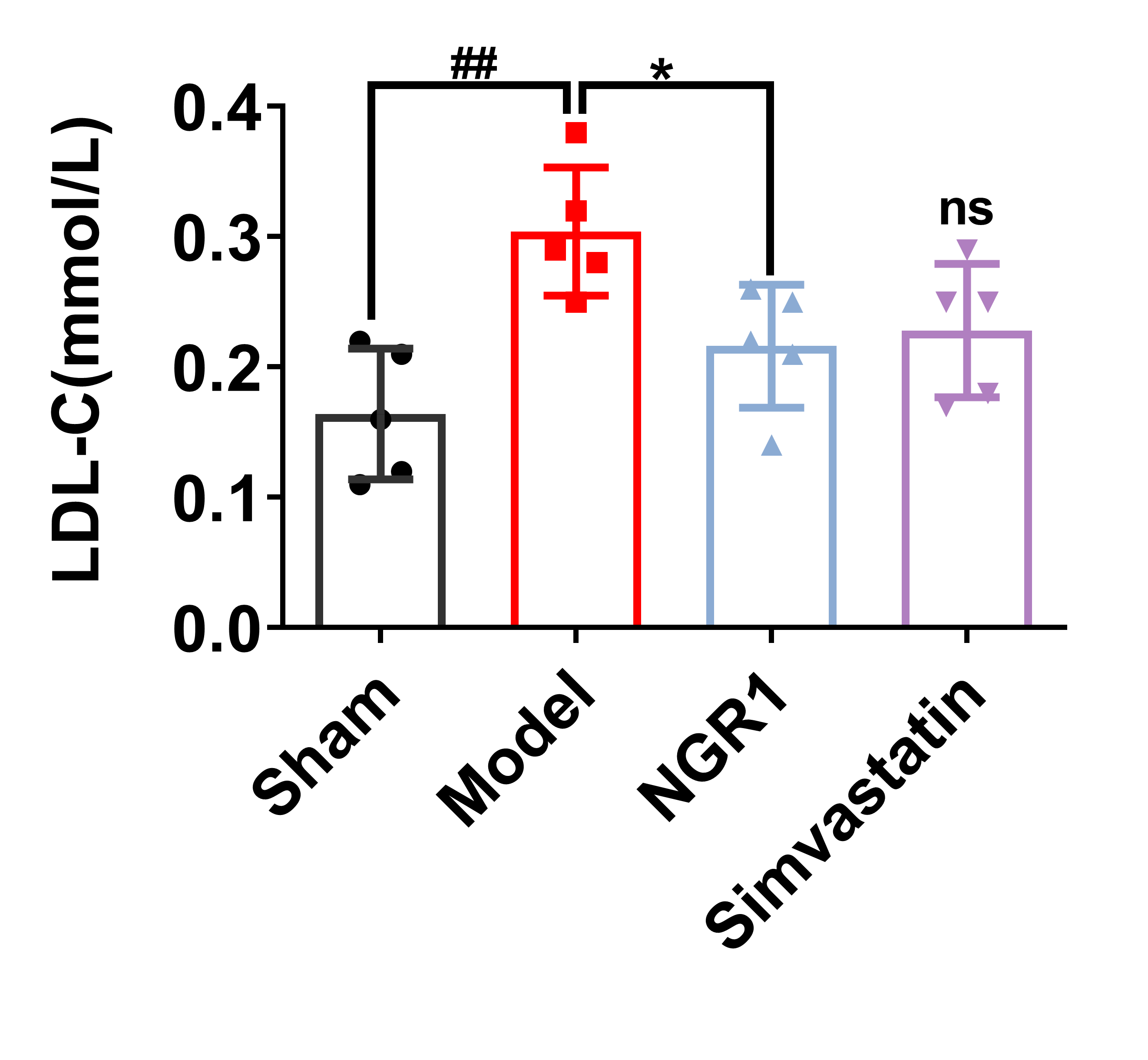

Supplement: Supplementary file 4 [file DataSheet9.ZIP › Figure 2/LDL-C.tif]

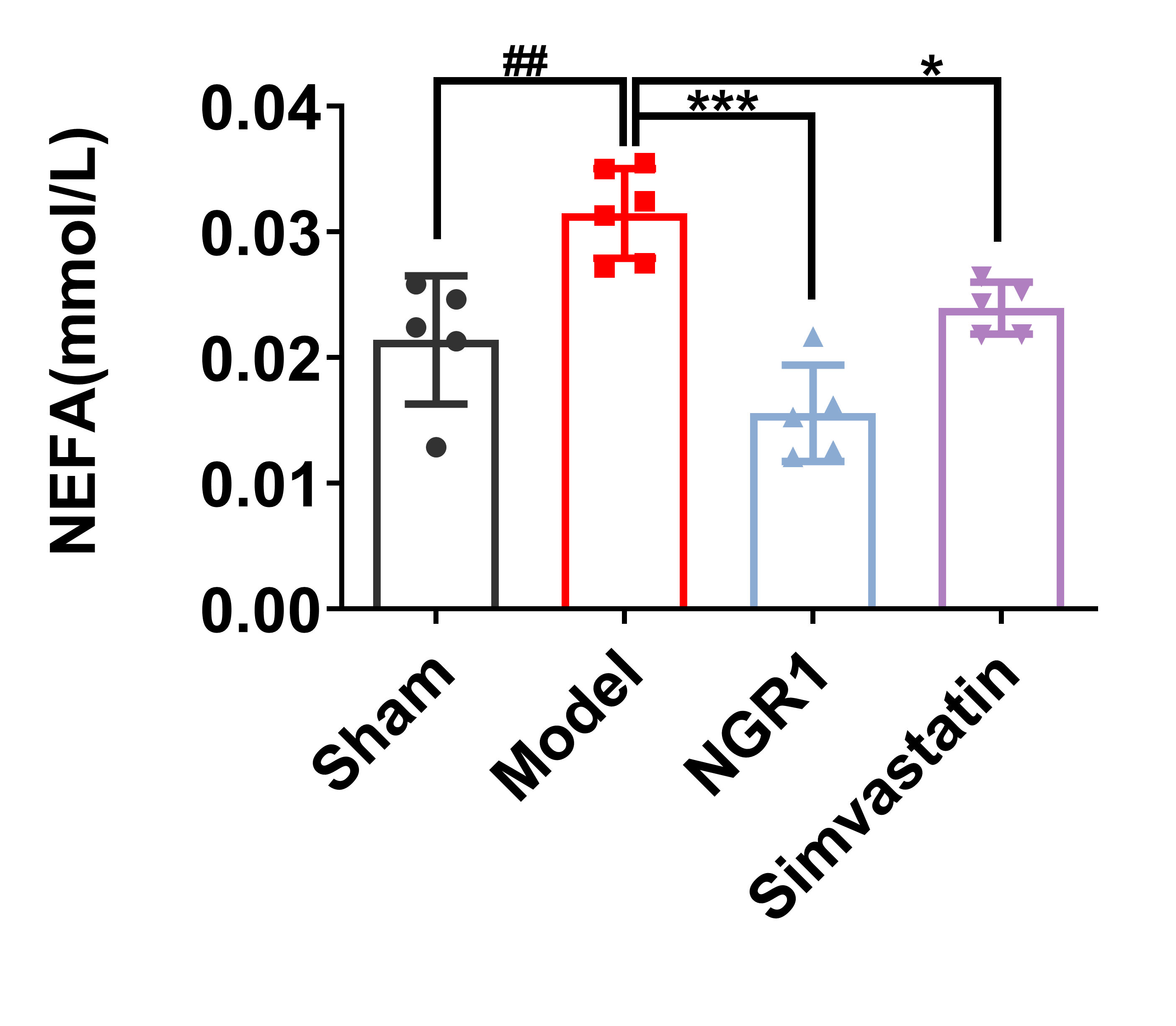

Supplement: Supplementary file 4 [file DataSheet9.ZIP › Figure 2/NEFA-C.tif]

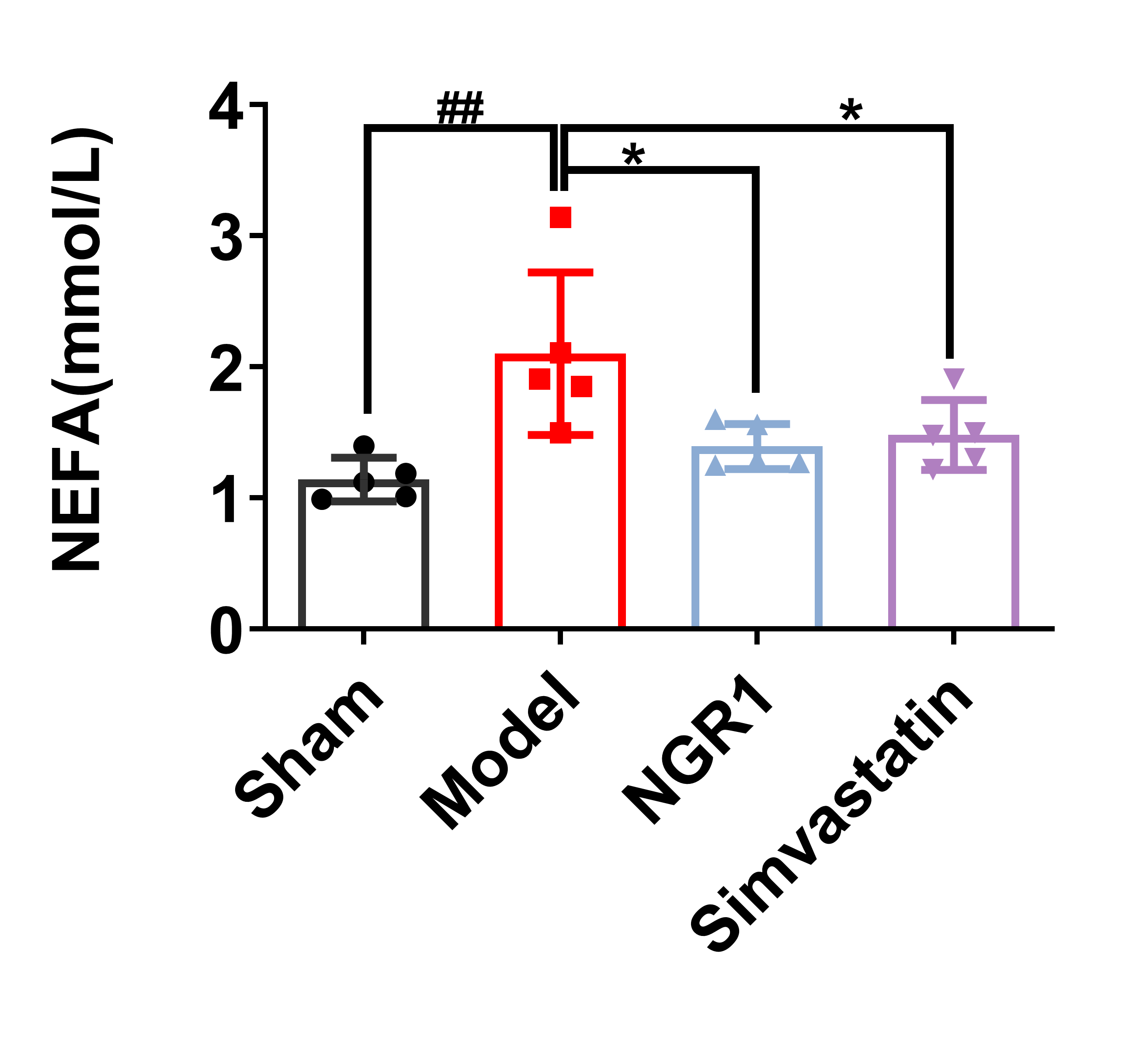

Supplement: Supplementary file 4 [file DataSheet9.ZIP › Figure 2/NEFA-H.tif]

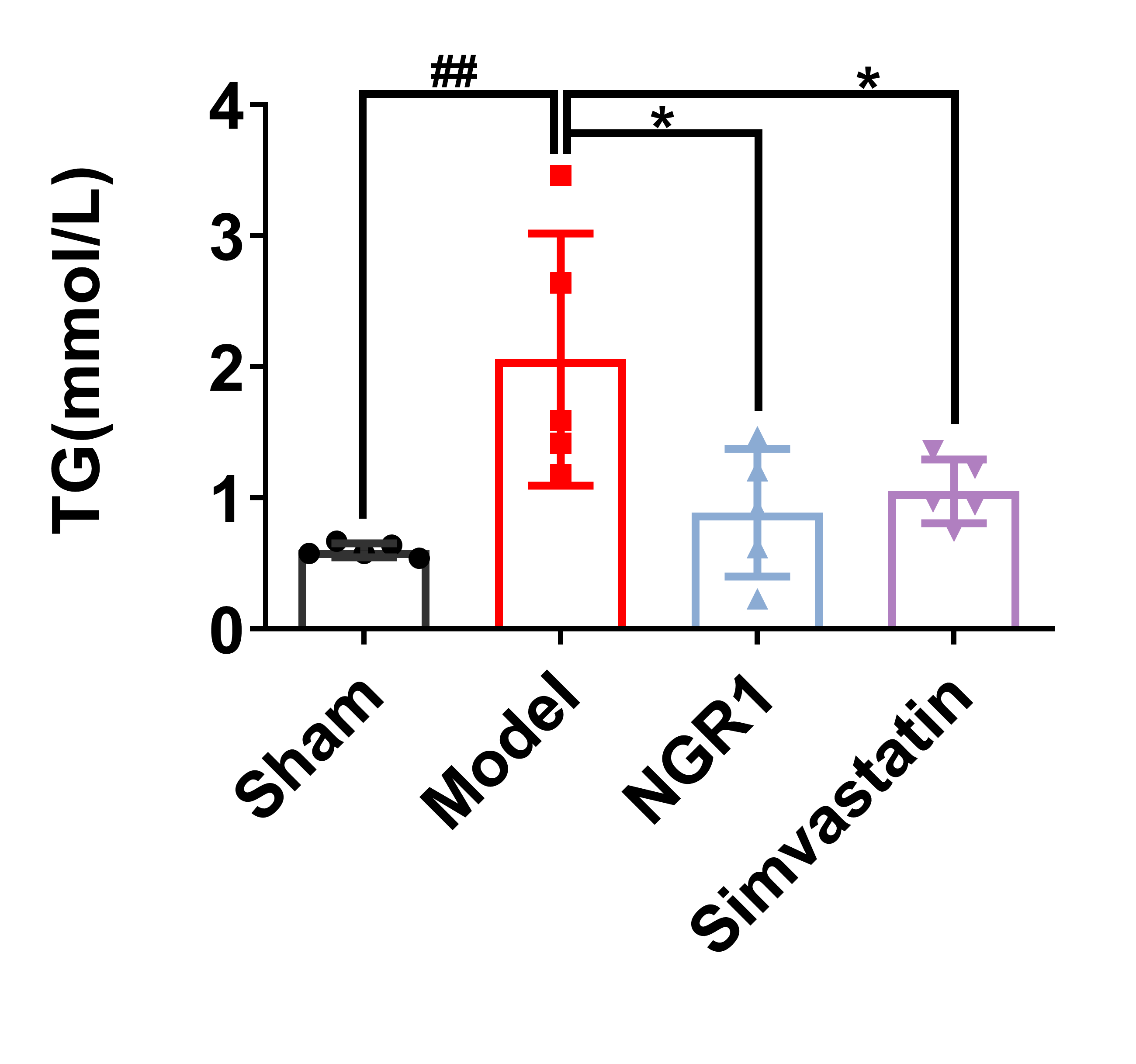

Supplement: Supplementary file 4 [file DataSheet9.ZIP › Figure 2/TG.tif]

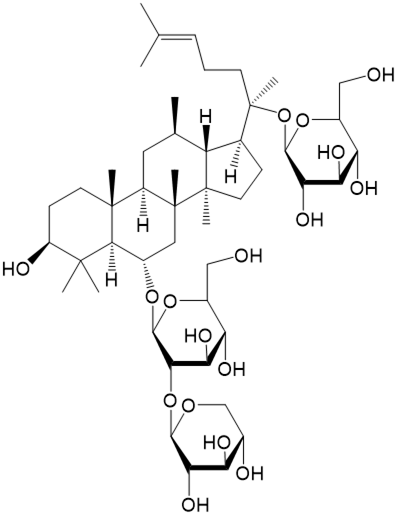

Supplement: Supplementary file 4 [file DataSheet9.ZIP › Figure 4/Fig 4A/Fig 4A.tif]

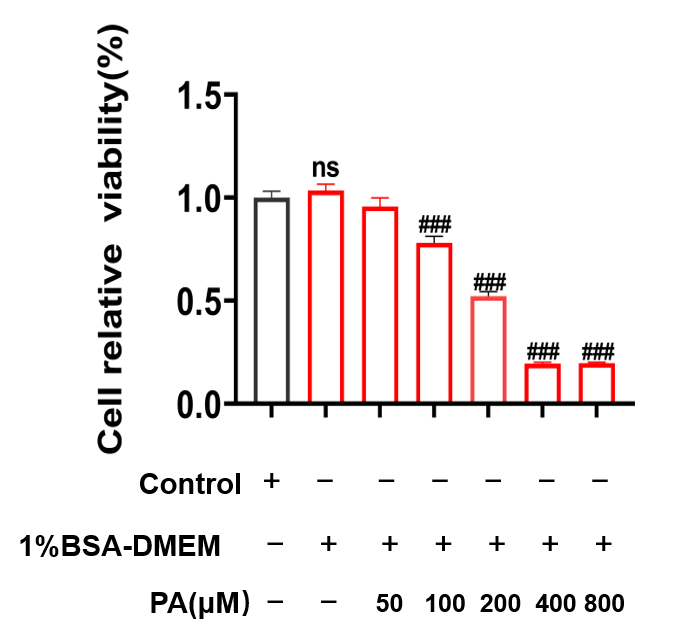

Supplement: Supplementary file 4 [file DataSheet9.ZIP › Figure 4/Fig 4B/Fig 4B.tif]

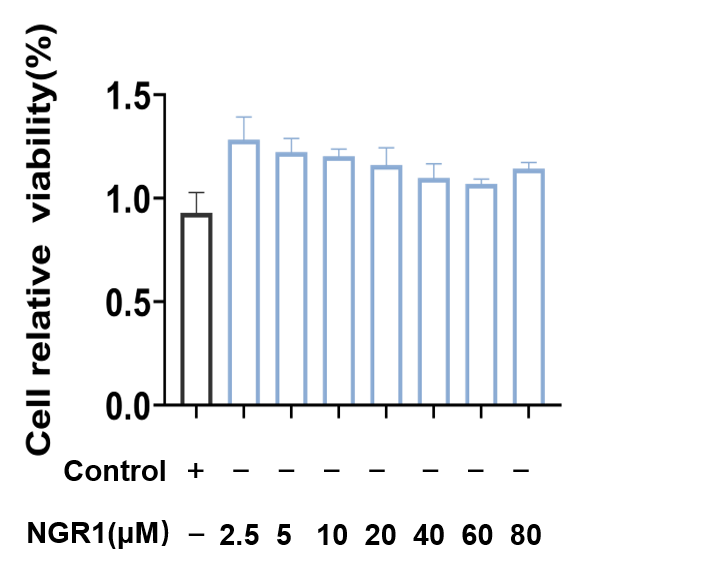

Supplement: Supplementary file 4 [file DataSheet9.ZIP › Figure 4/Fig 4C/Fig 4C.tif]

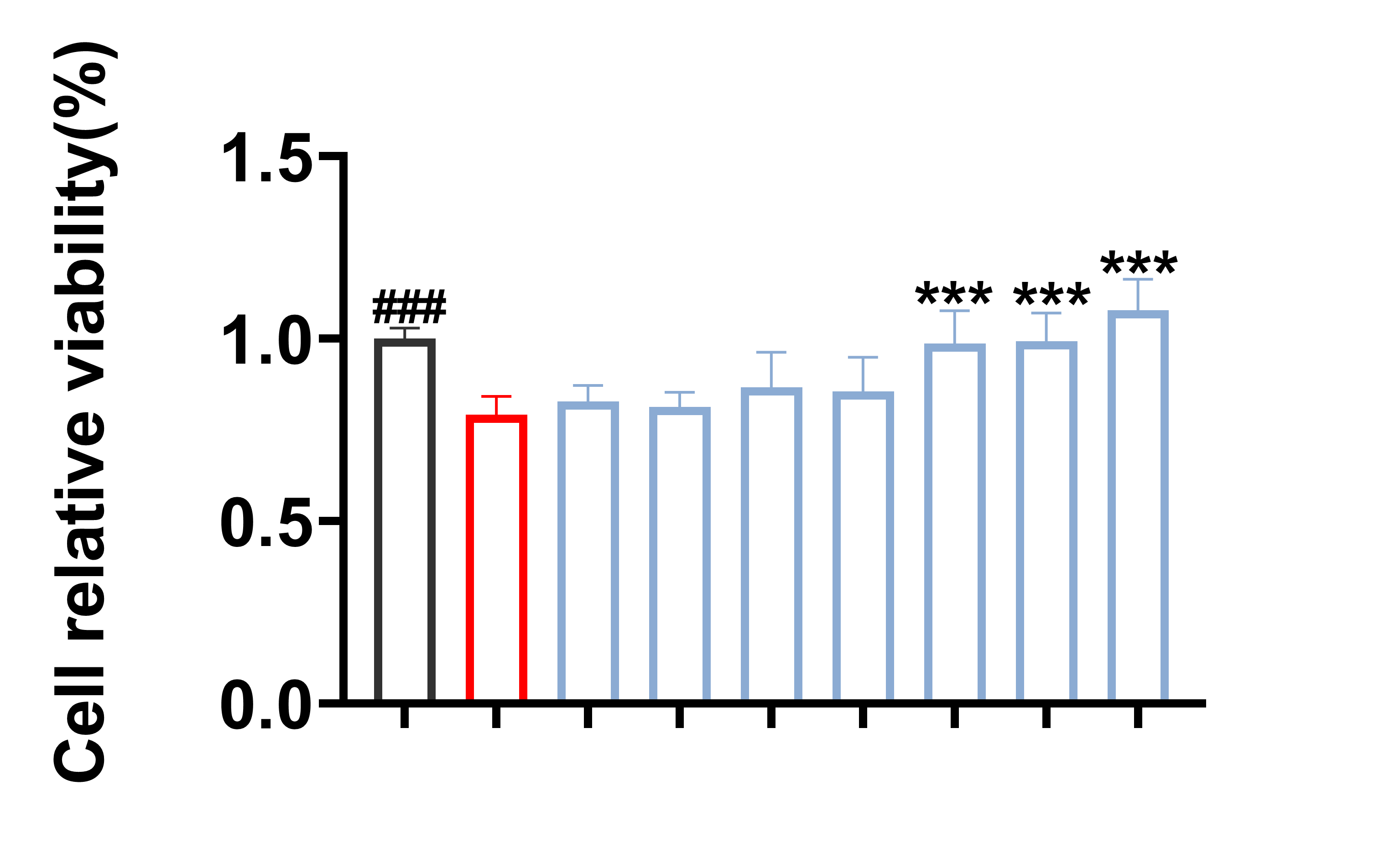

Supplement: Supplementary file 4 [file DataSheet9.ZIP › Figure 4/Fig 4D/Fig 4D.tif]

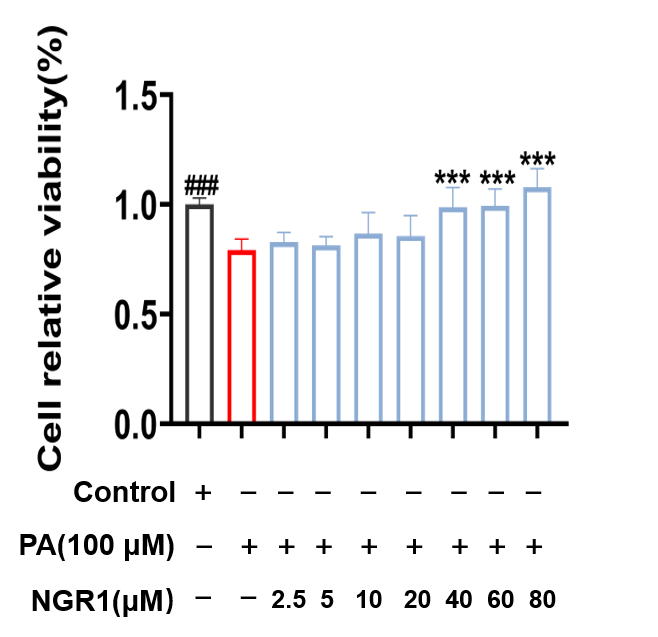

Supplement: Supplementary file 4 [file DataSheet9.ZIP › Figure 4/Fig 4D/Figure 4D.tif]

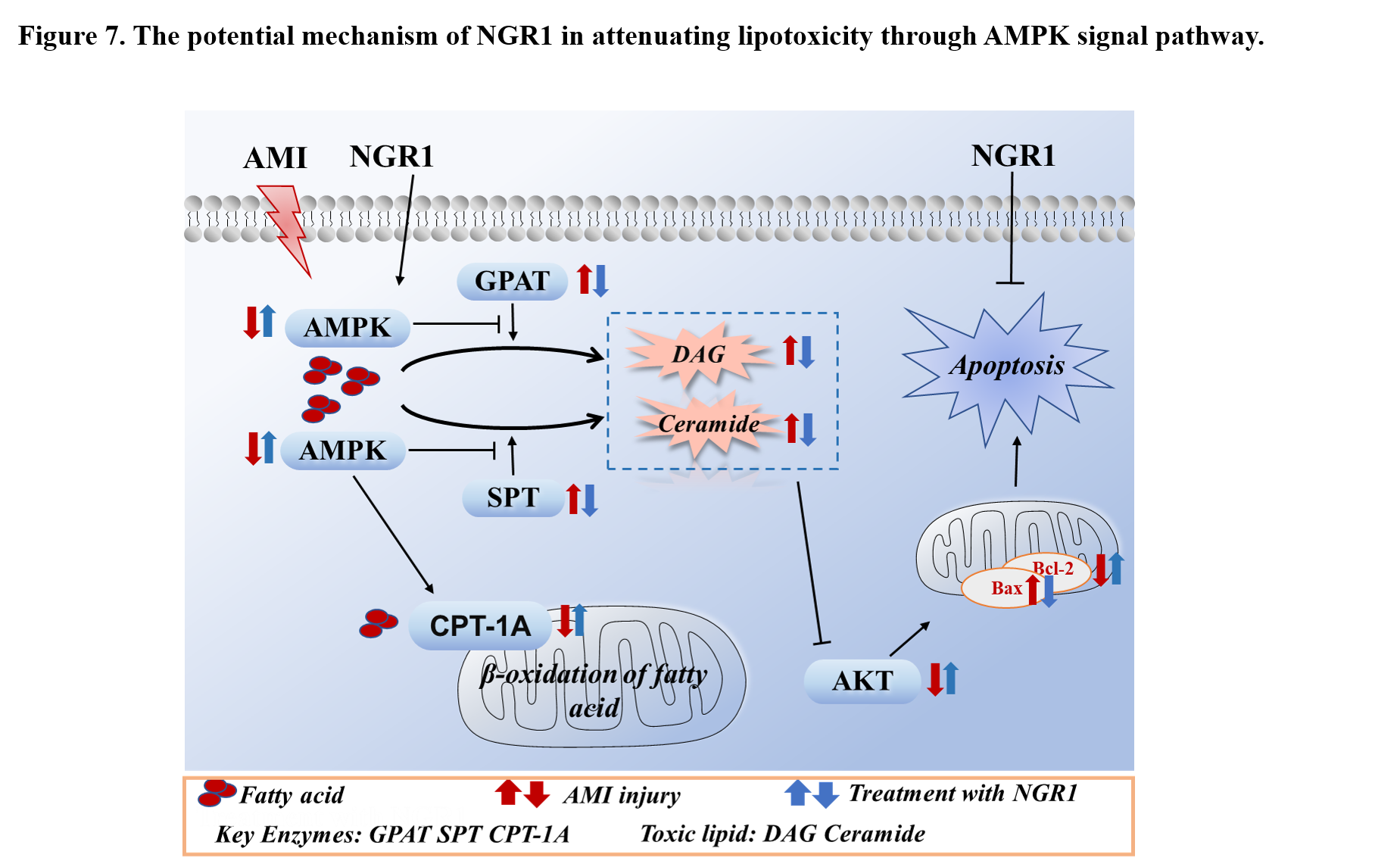

Supplement: Supplementary file 4 [file DataSheet9.ZIP › Figure 7/Figure 7.tif]

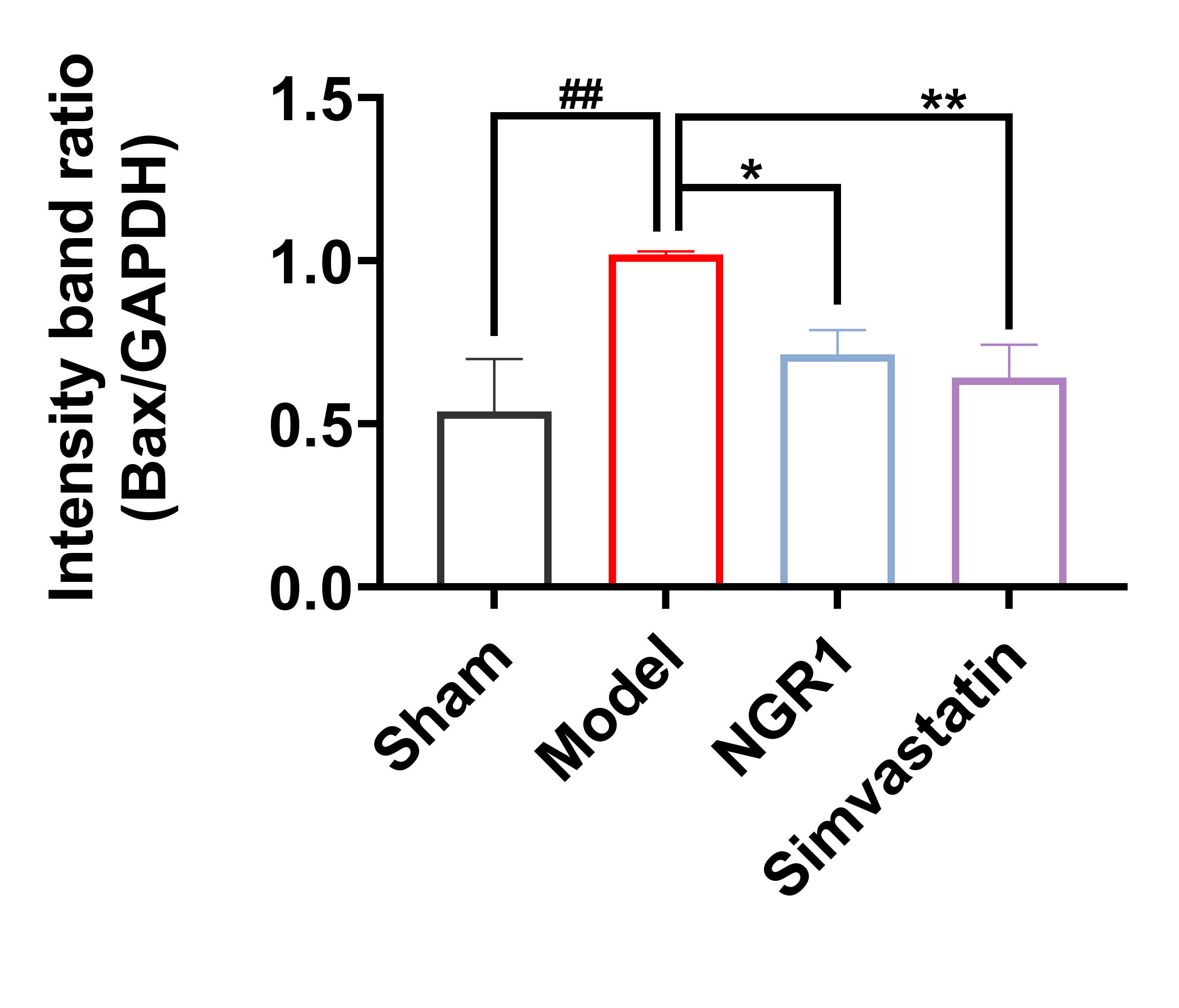

Supplement: Supplementary file 5 [file DataSheet4.ZIP › Figure 3(4)/Bax.tif]

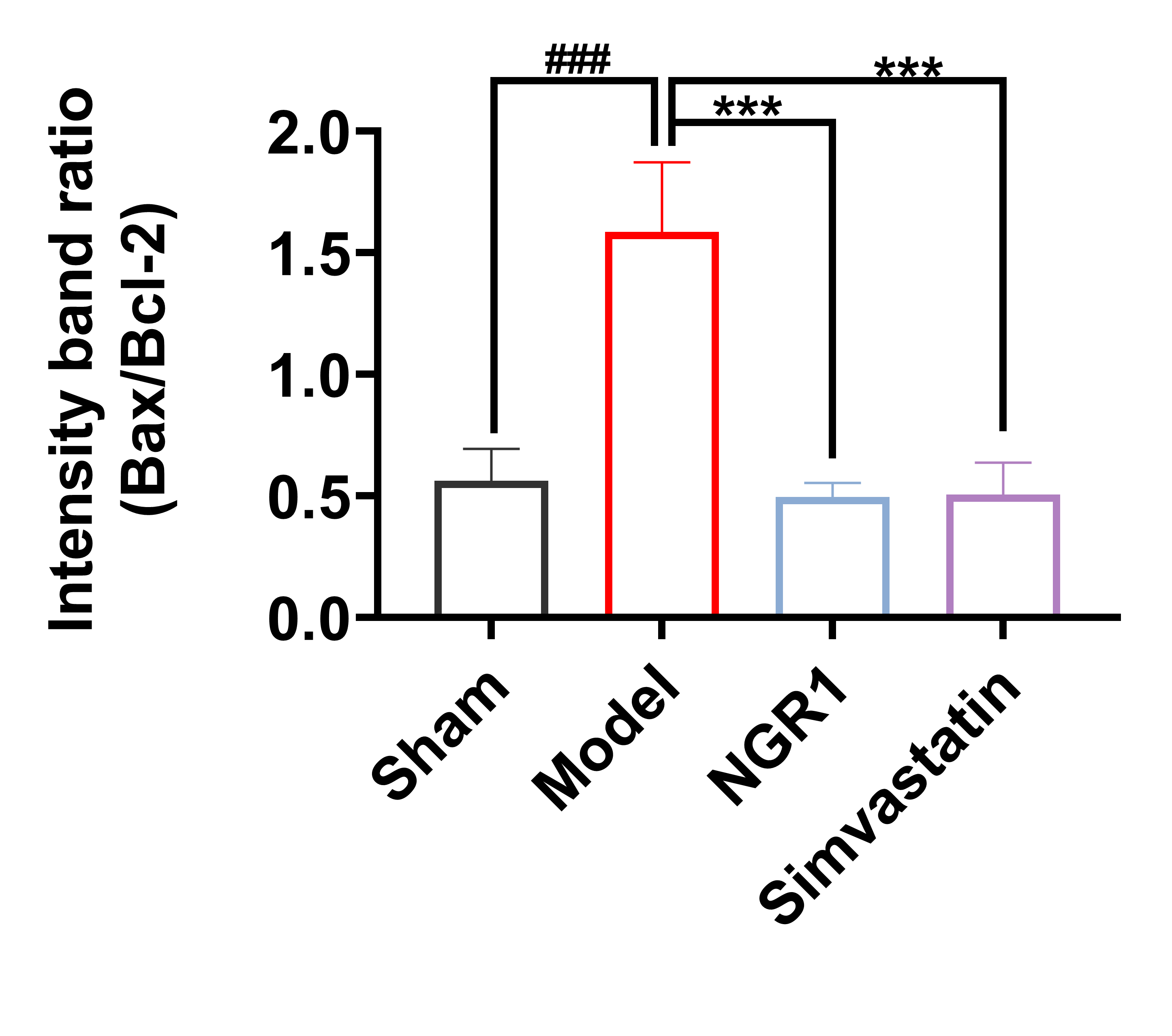

Supplement: Supplementary file 5 [file DataSheet4.ZIP › Figure 3(4)/Bax_Bcl-2.tif]

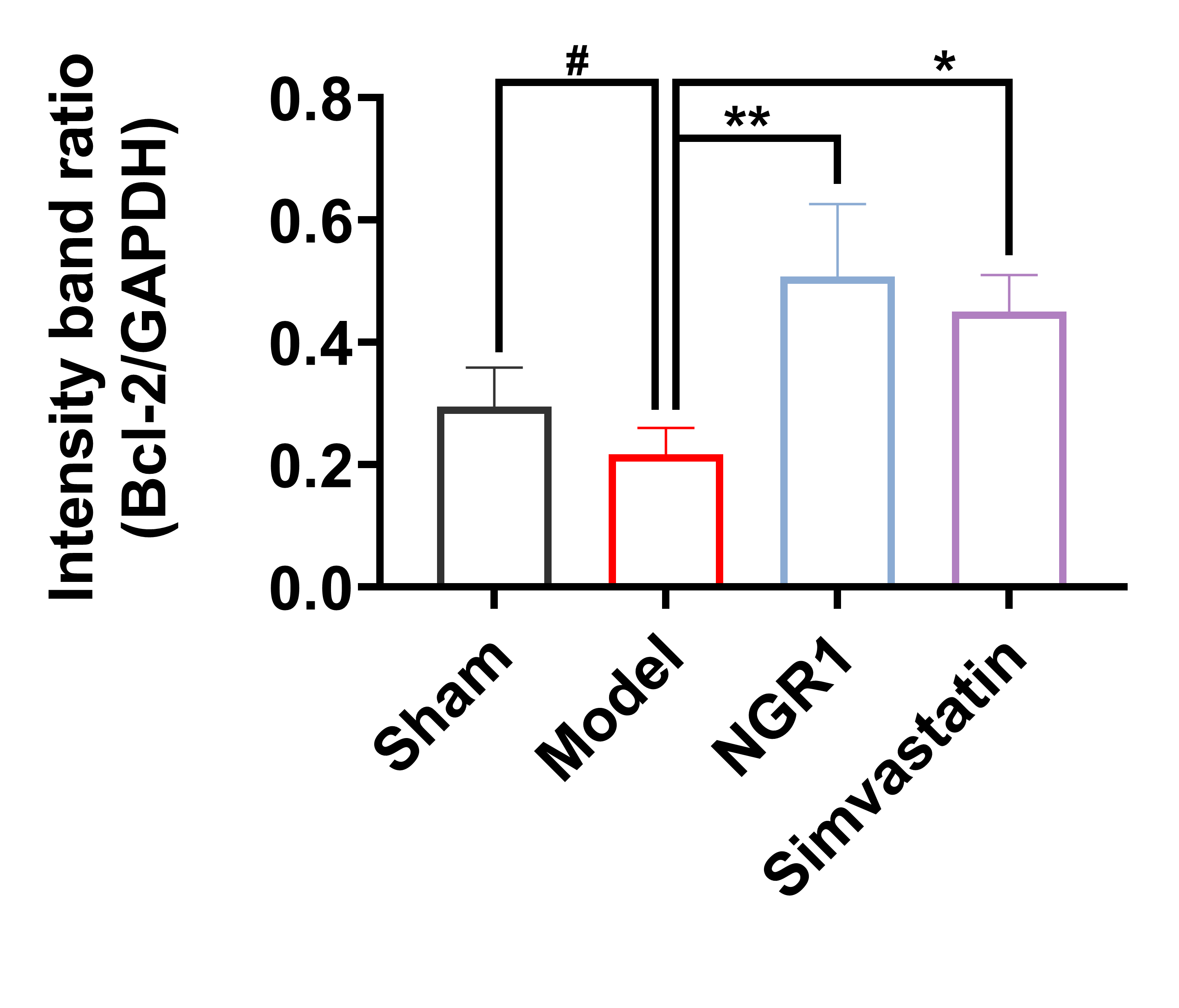

Supplement: Supplementary file 5 [file DataSheet4.ZIP › Figure 3(4)/Bcl-2.tif]

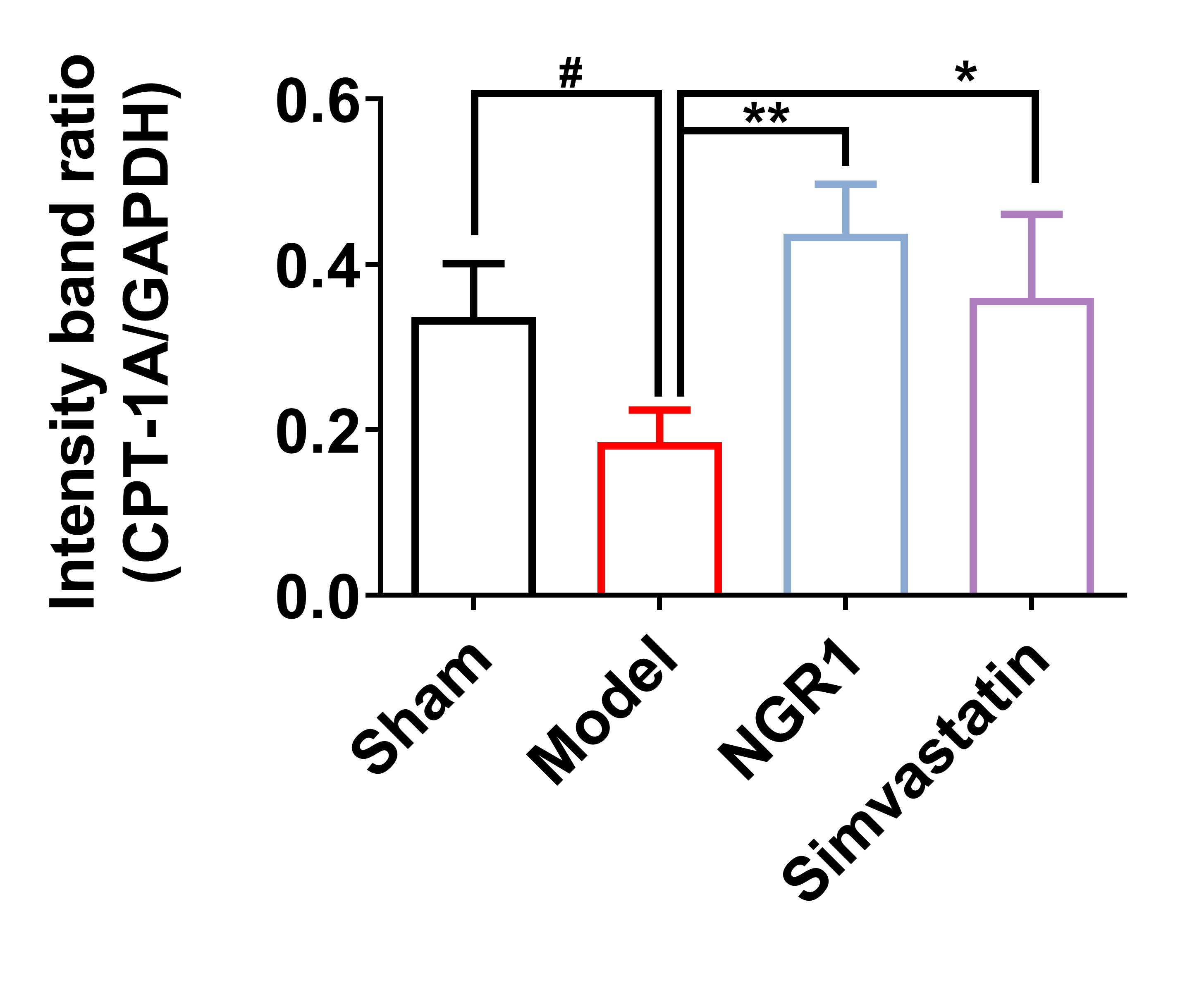

Supplement: Supplementary file 5 [file DataSheet4.ZIP › Figure 3(4)/CPT-1A.tif]

## Slide 1
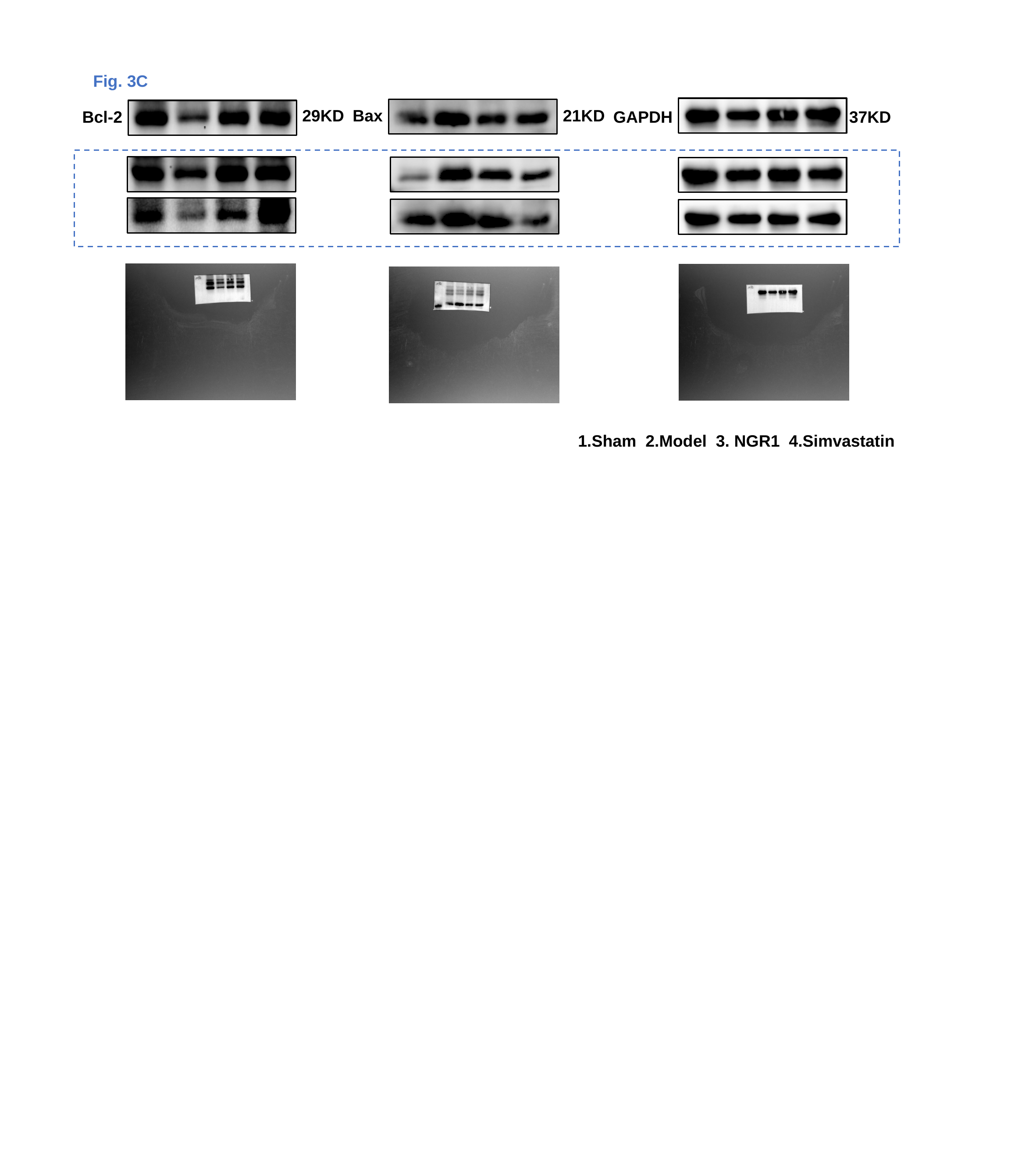

Fig. 3C
GAPDH
37KD
21KD
Bax
29KD
Bcl-2
1.Sham 2.Model 3. NGR1 4.Simvastatin

Supplement: Supplementary file 5 [file DataSheet4.ZIP › Figure 3(4)/Figure 3-4.pptx]

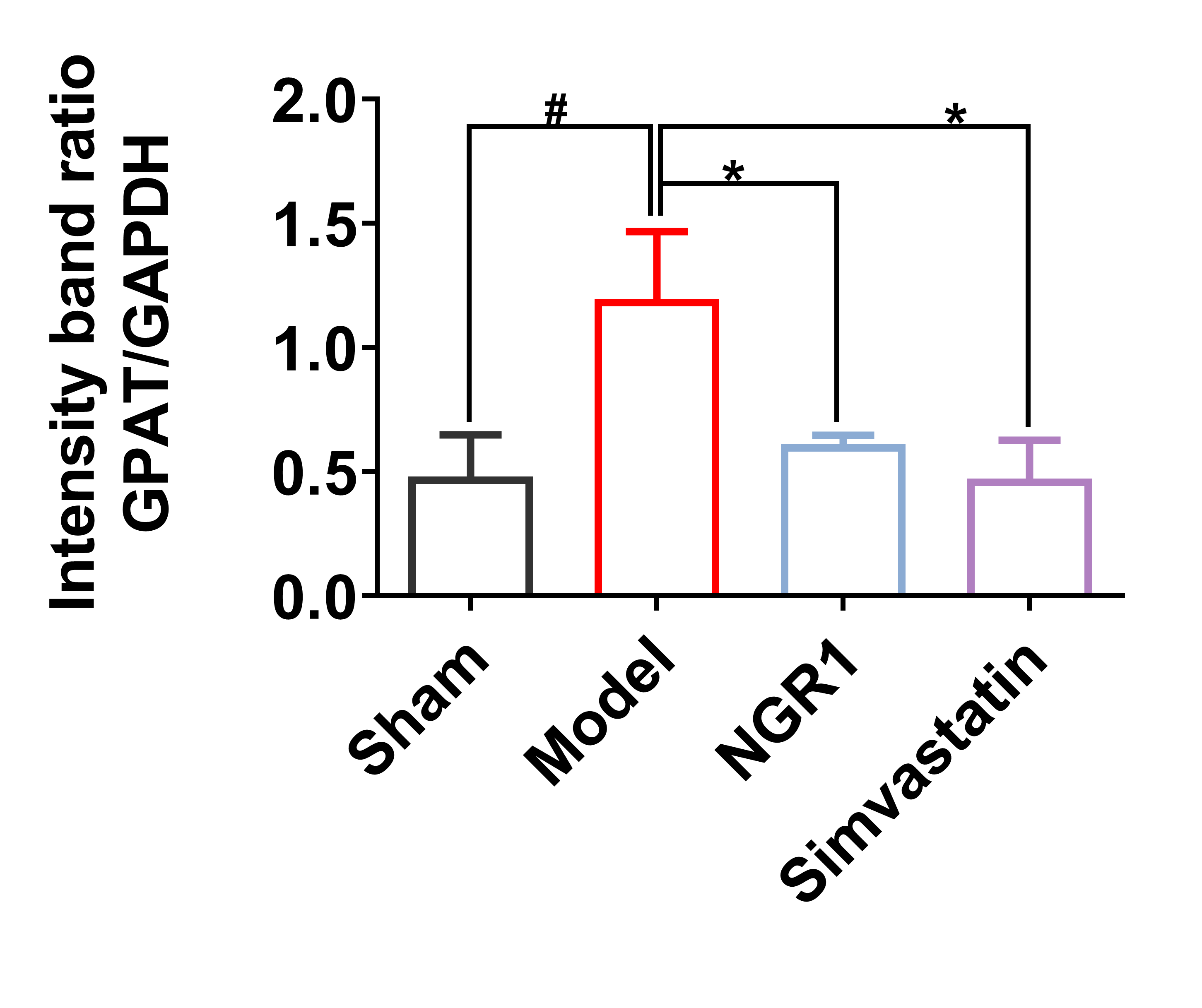

Supplement: Supplementary file 5 [file DataSheet4.ZIP › Figure 3(4)/GPAT.tif]

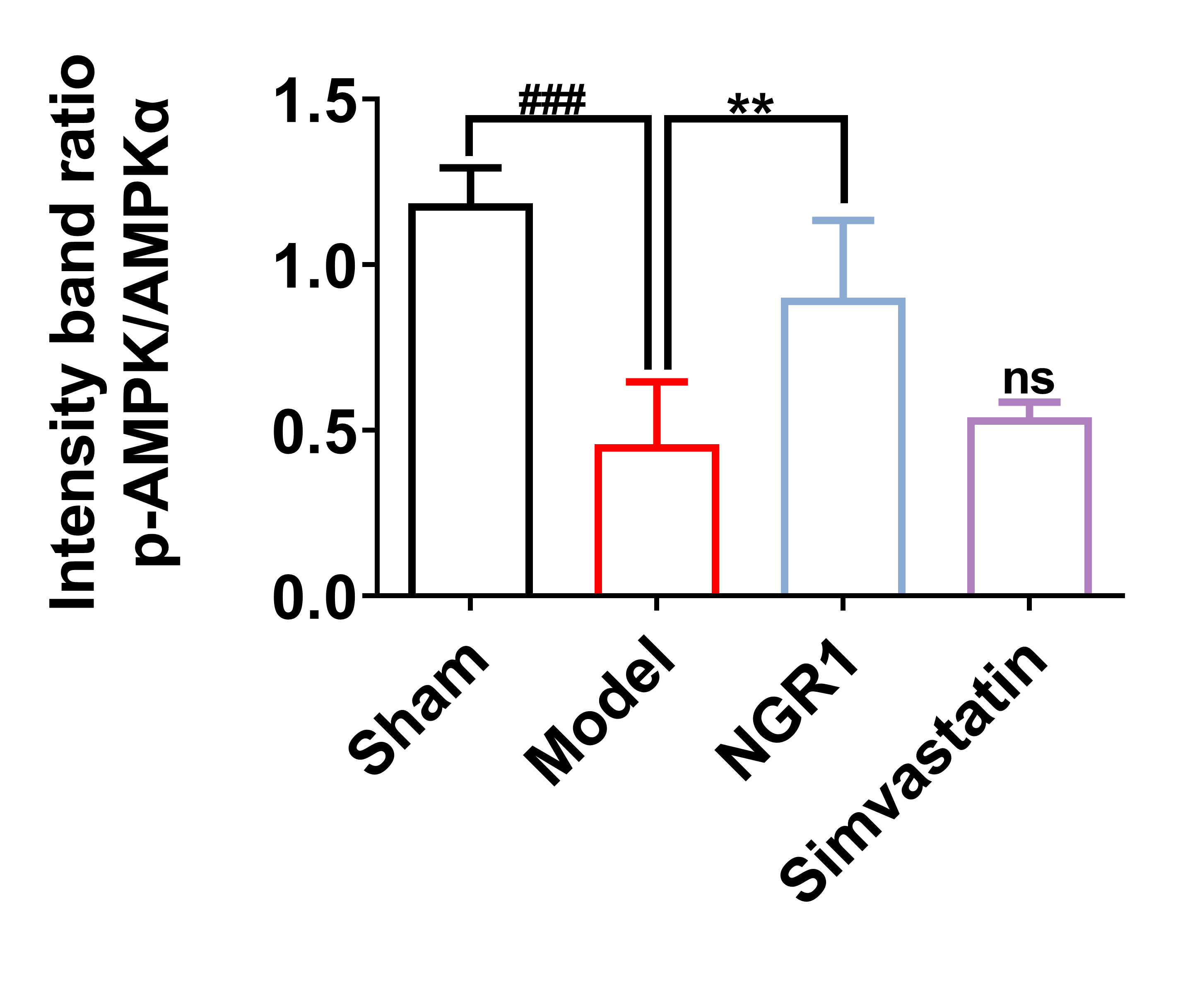

Supplement: Supplementary file 5 [file DataSheet4.ZIP › Figure 3(4)/p-AMPK_AMPK.tif]

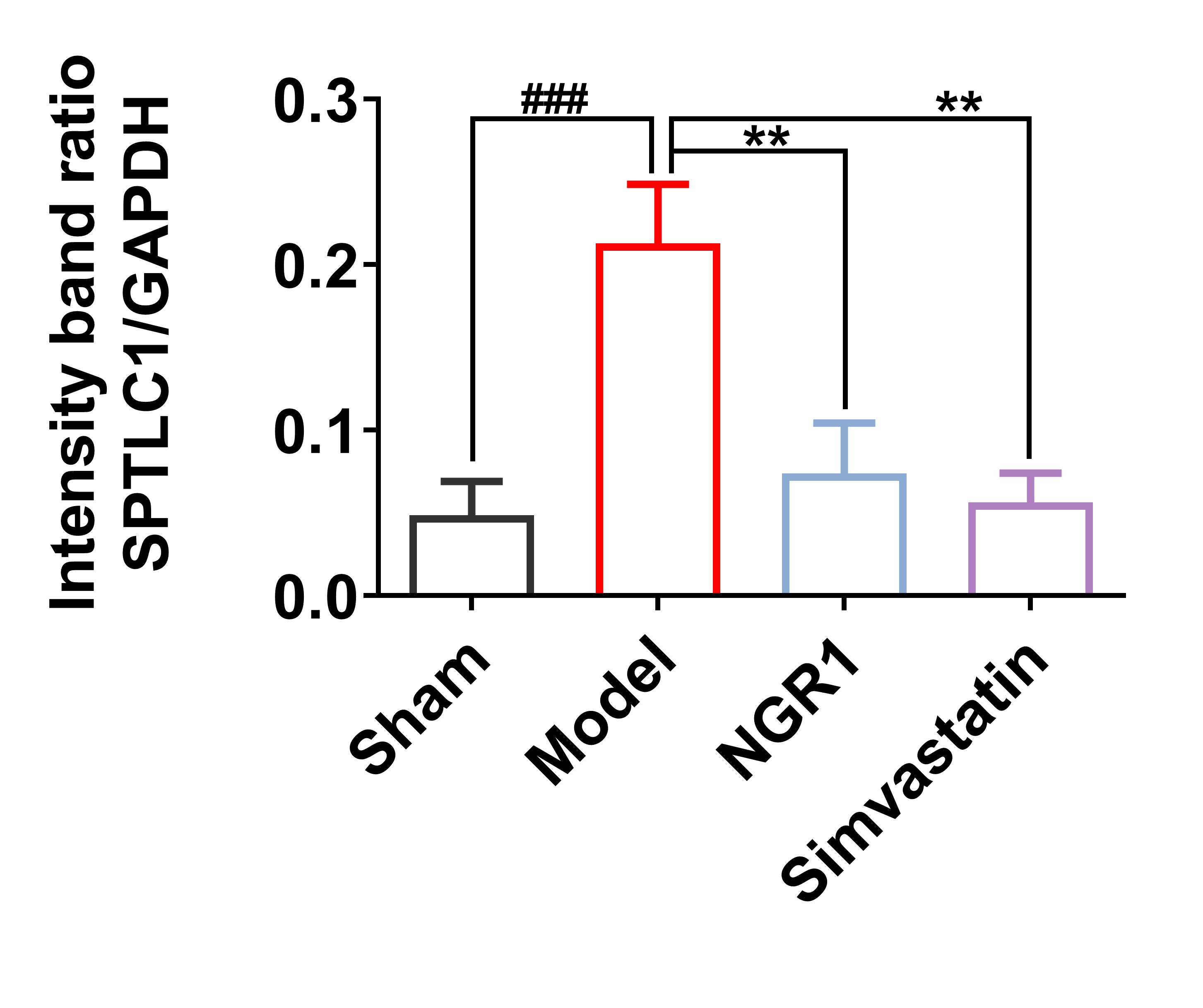

Supplement: Supplementary file 5 [file DataSheet4.ZIP › Figure 3(4)/SPTLC1.tif]

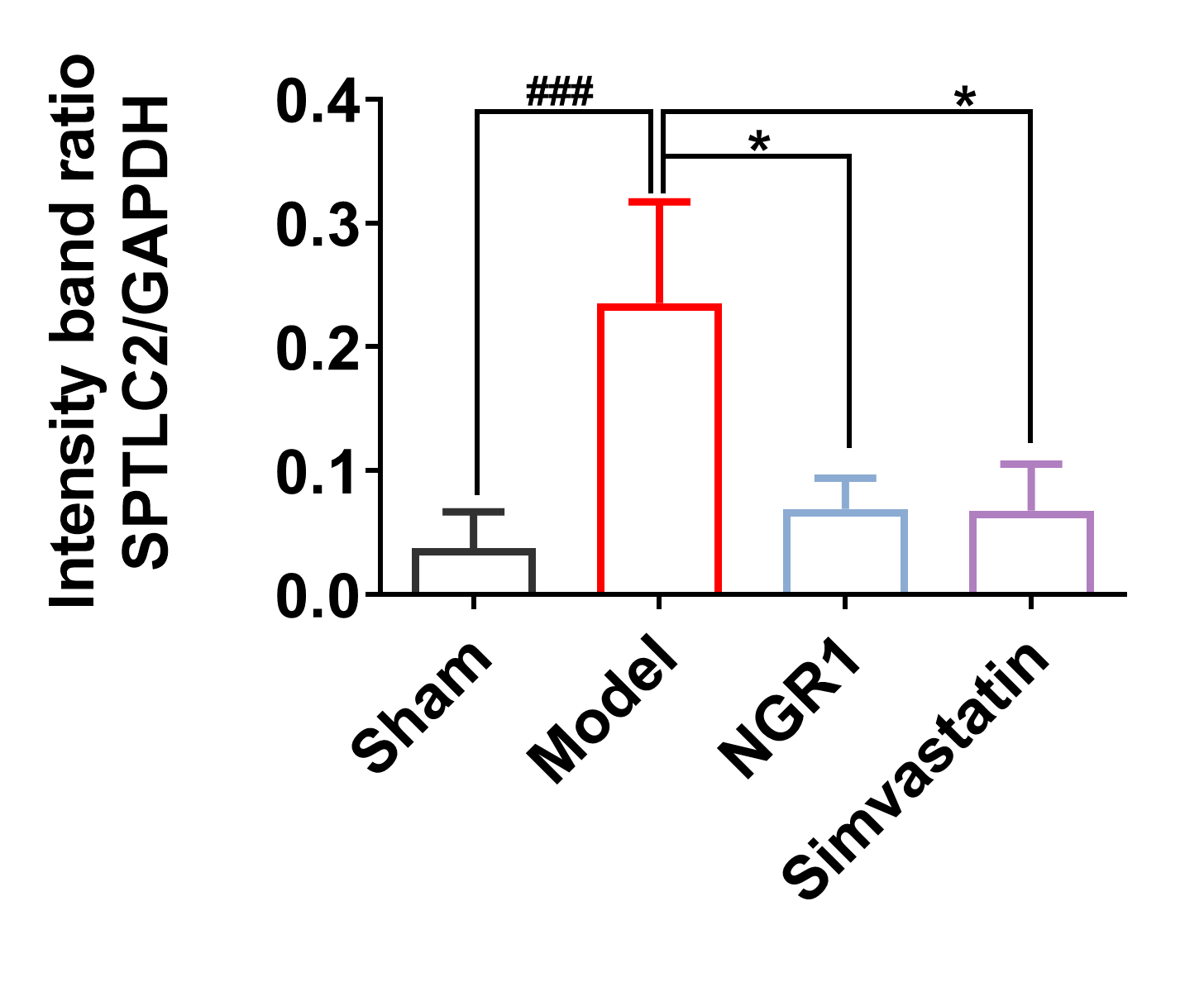

Supplement: Supplementary file 5 [file DataSheet4.ZIP › Figure 3(4)/SPTLC2.tif]

## Slide 1
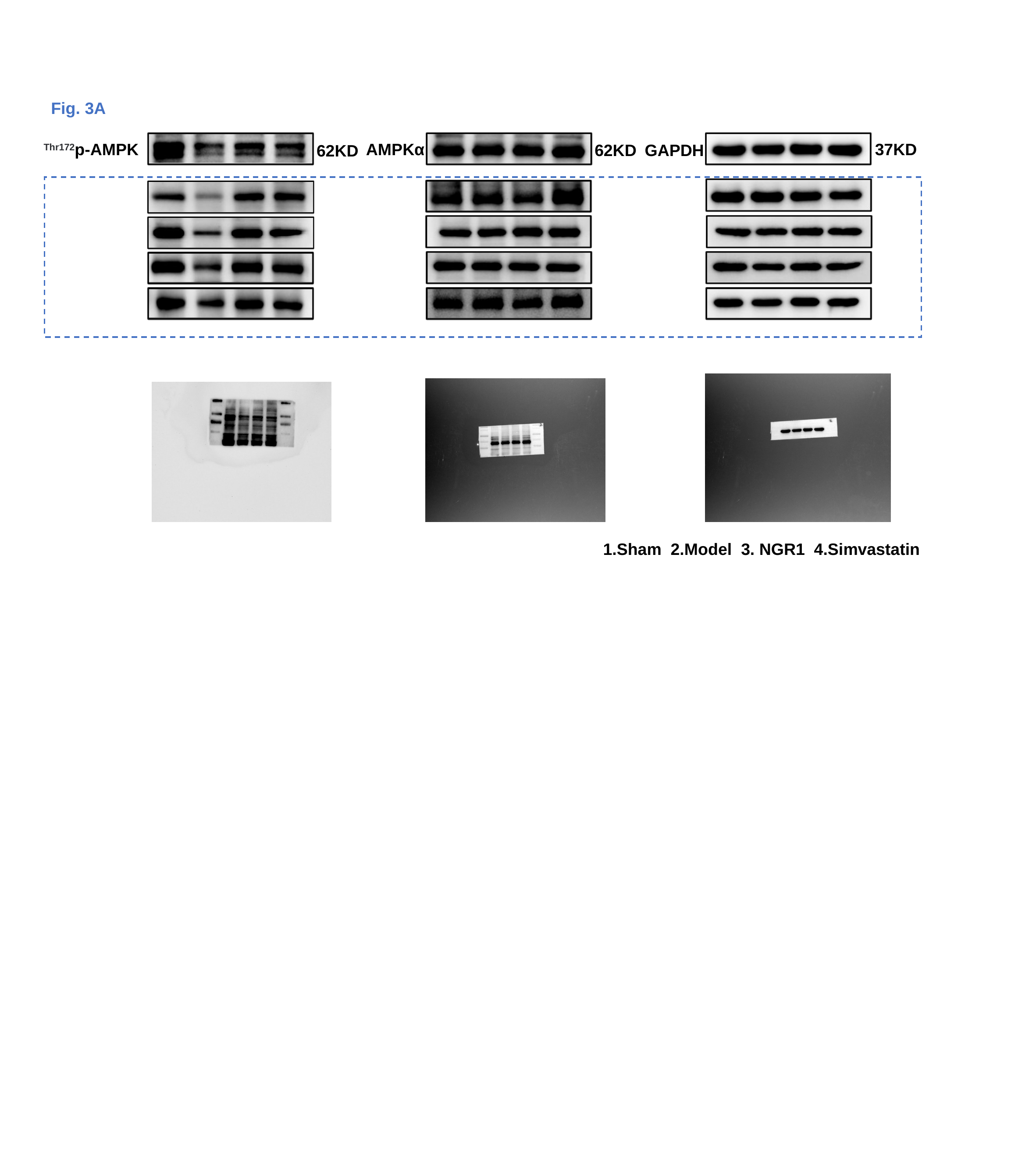

Fig. 3A
AMPKα
37KD
Thr172p-AMPK
GAPDH
62KD
62KD
1.Sham 2.Model 3. NGR1 4.Simvastatin

Supplement: Supplementary file 5 [file DataSheet4.ZIP › Figure 3.pptx]

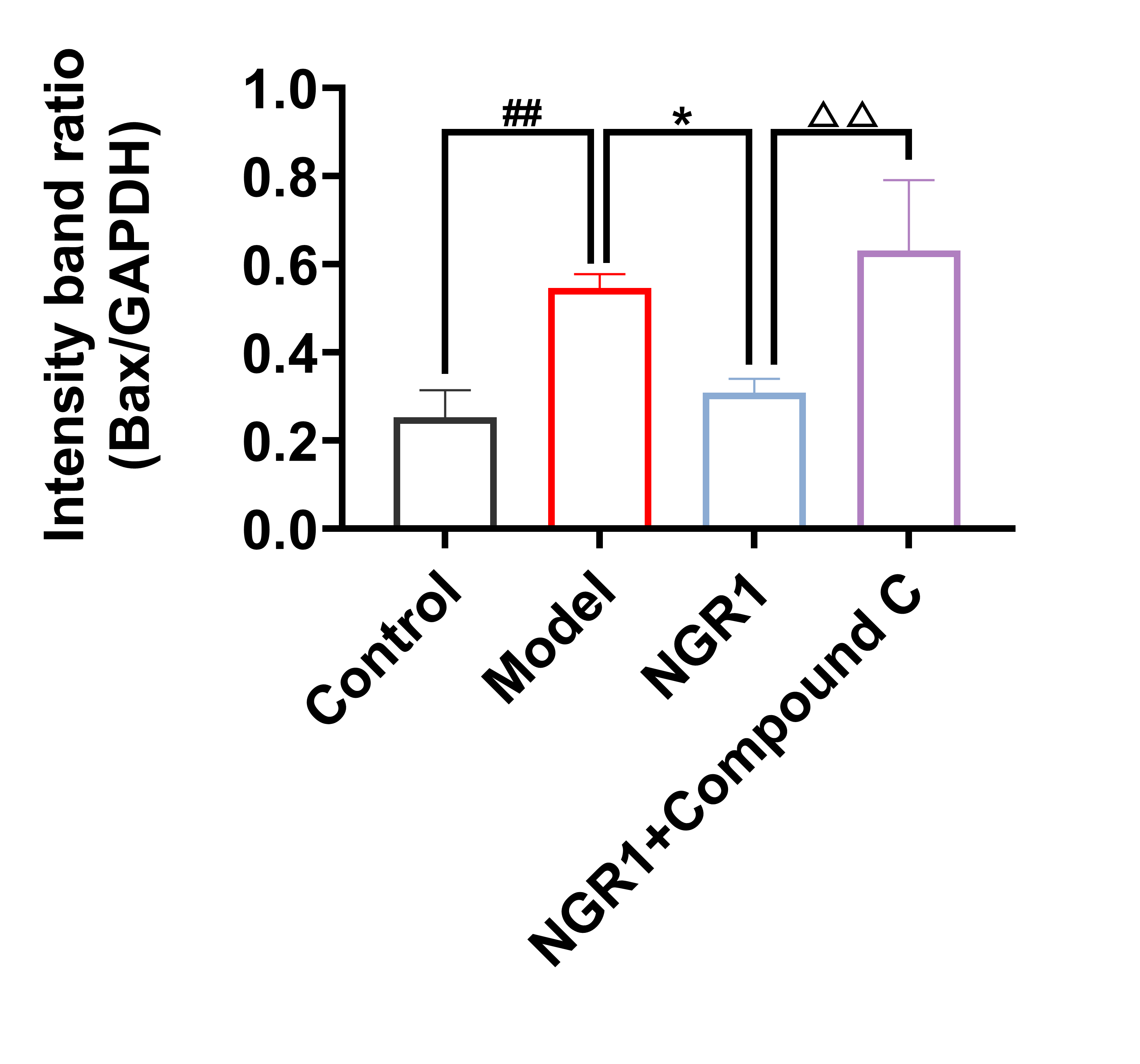

Supplement: Supplementary file 6 [file DataSheet1.ZIP › BAX.tif]

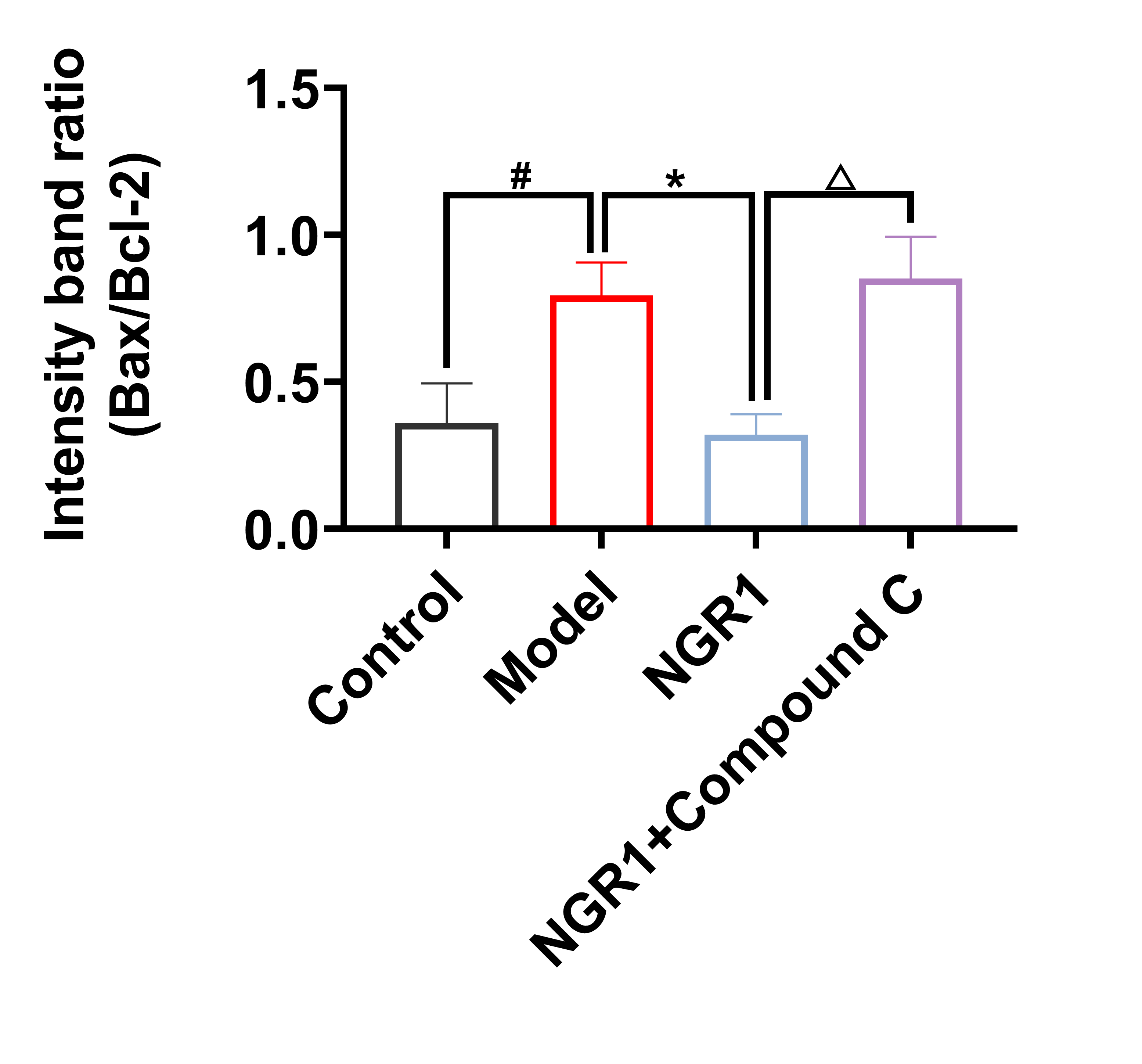

Supplement: Supplementary file 6 [file DataSheet1.ZIP › Bax_Bcl-2.tif]

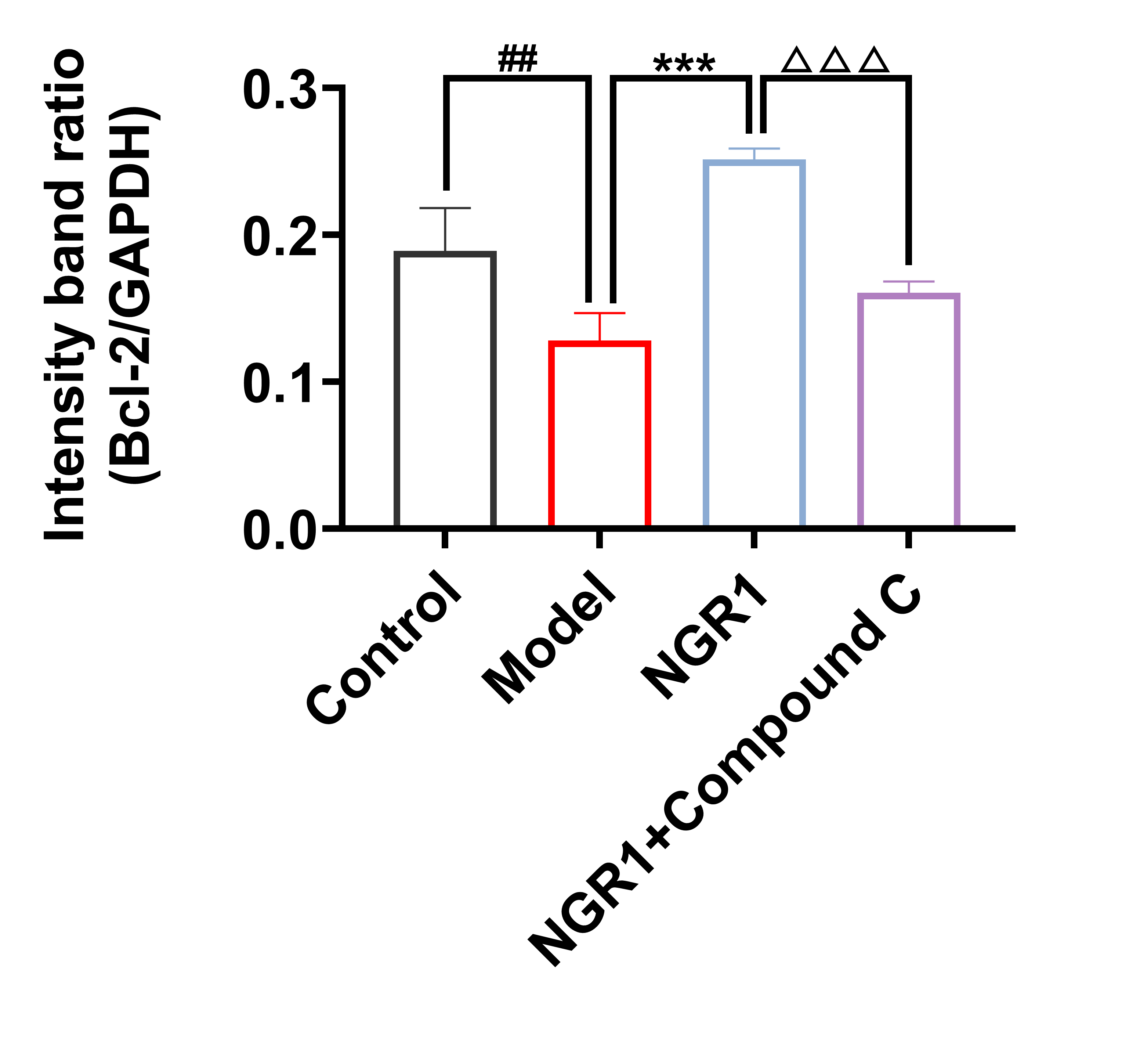

Supplement: Supplementary file 6 [file DataSheet1.ZIP › Bcl-2.tif]

## Slide 1
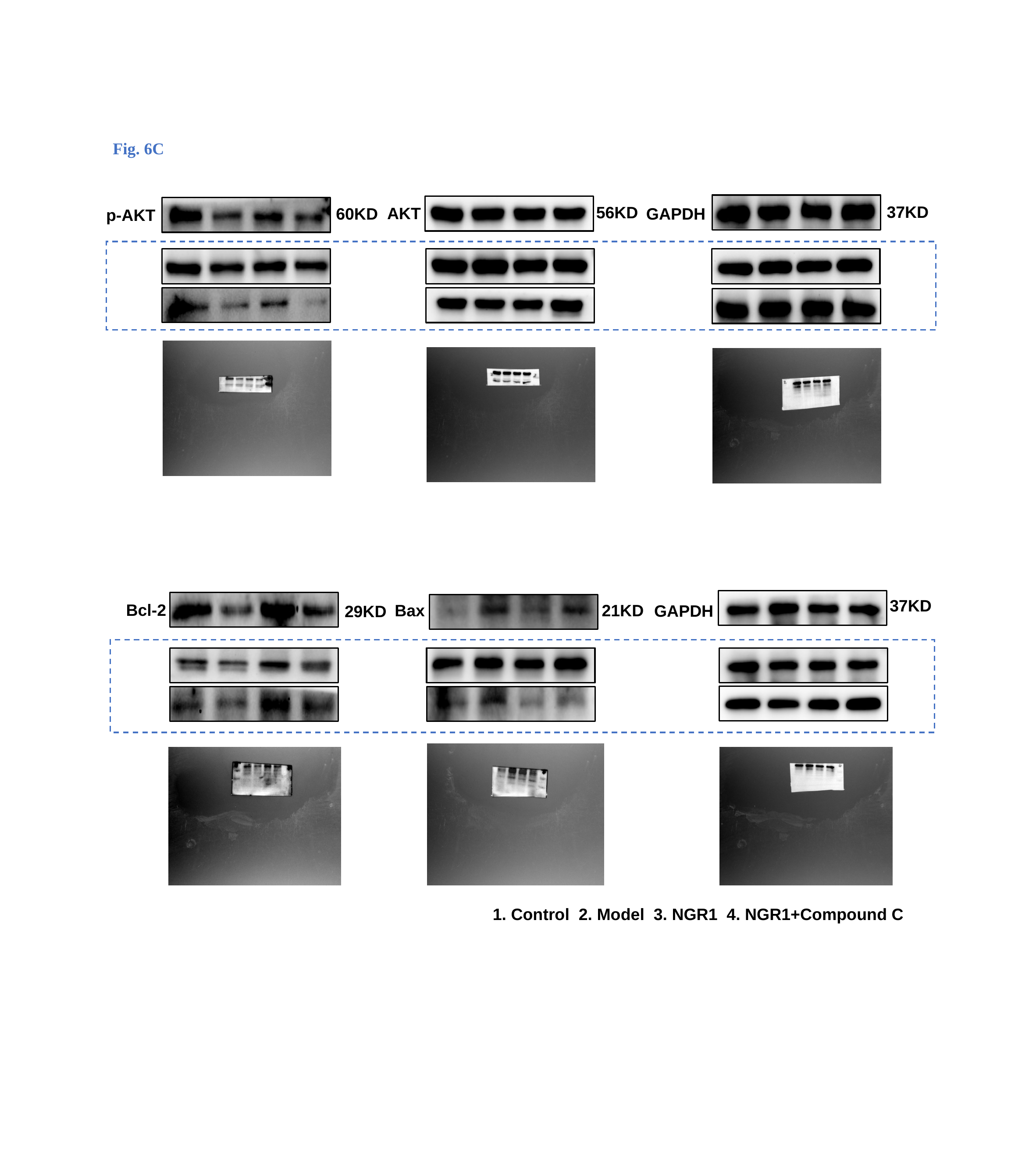

Fig. 6C
37KD
GAPDH
56KD
AKT
60KD
p-AKT
GAPDH
37KD
Bcl-2
29KD
21KD
Bax
1. Control 2. Model 3. NGR1 4. NGR1+Compound C

Supplement: Supplementary file 6 [file DataSheet1.ZIP › Figure 6C.pptx]

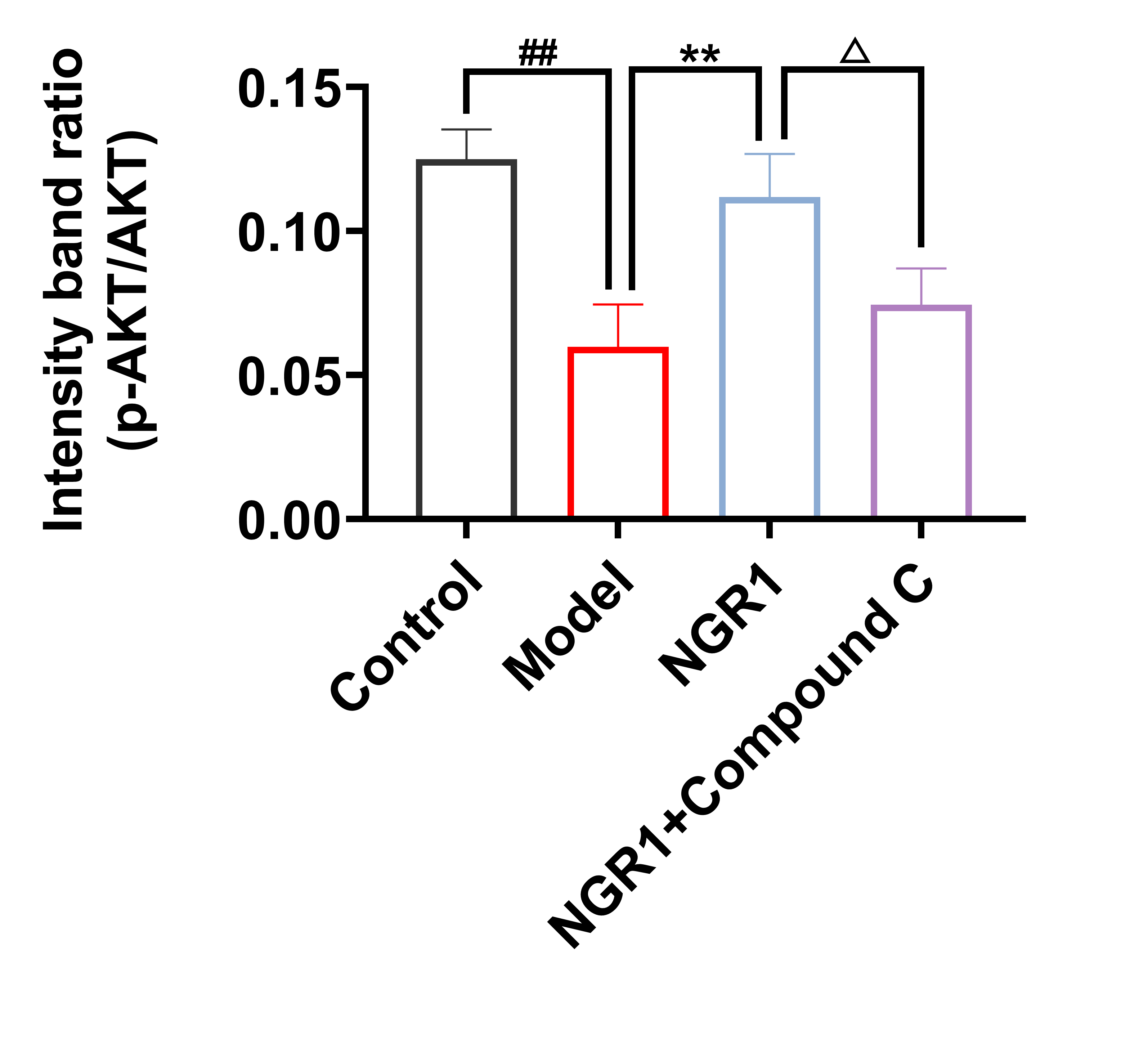

Supplement: Supplementary file 6 [file DataSheet1.ZIP › p-AKT_AKT.tif]

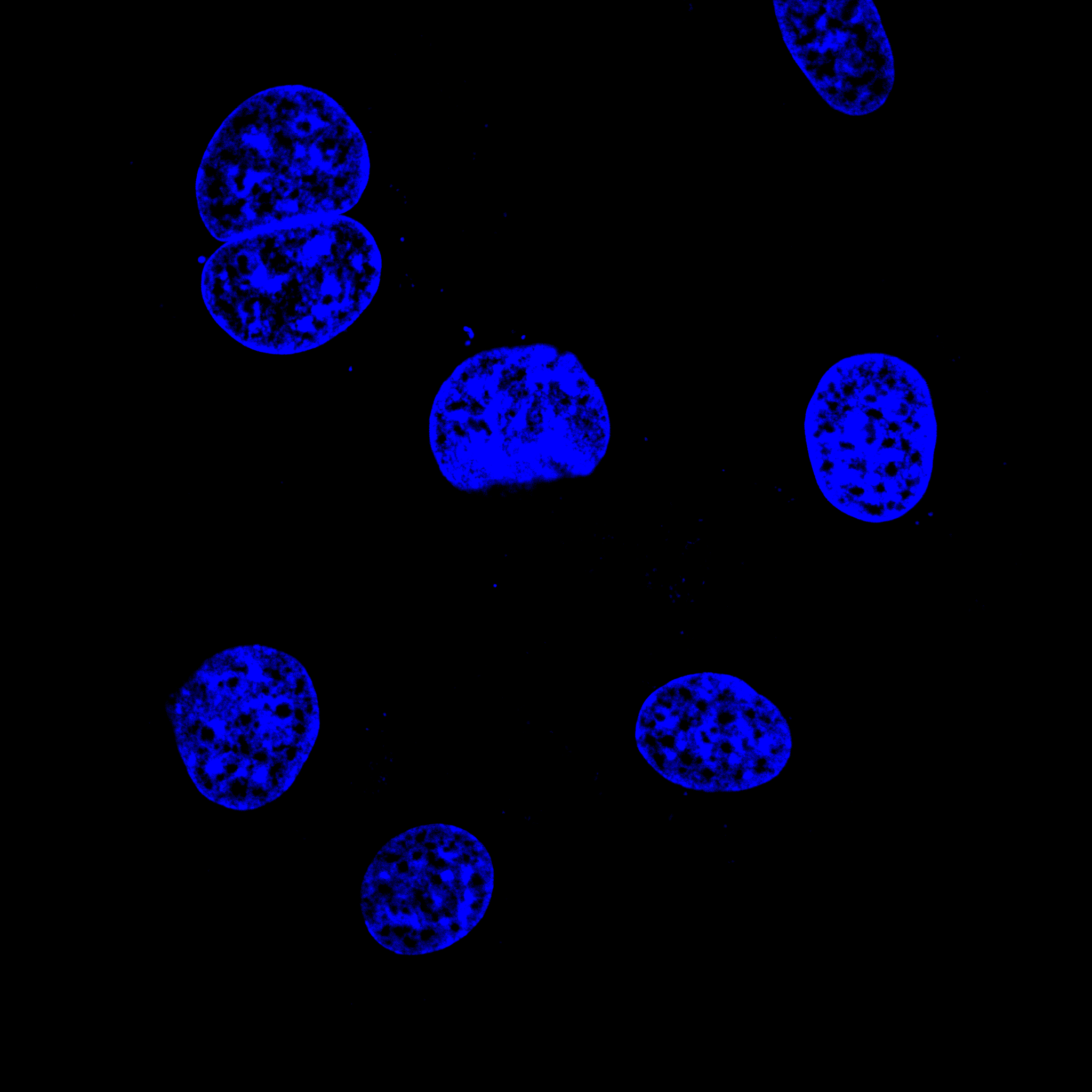

Supplement: Supplementary file 7 [file DataSheet10.ZIP › Fig 5D-E/Fig 5C/Control/Control-DAPI.tif]

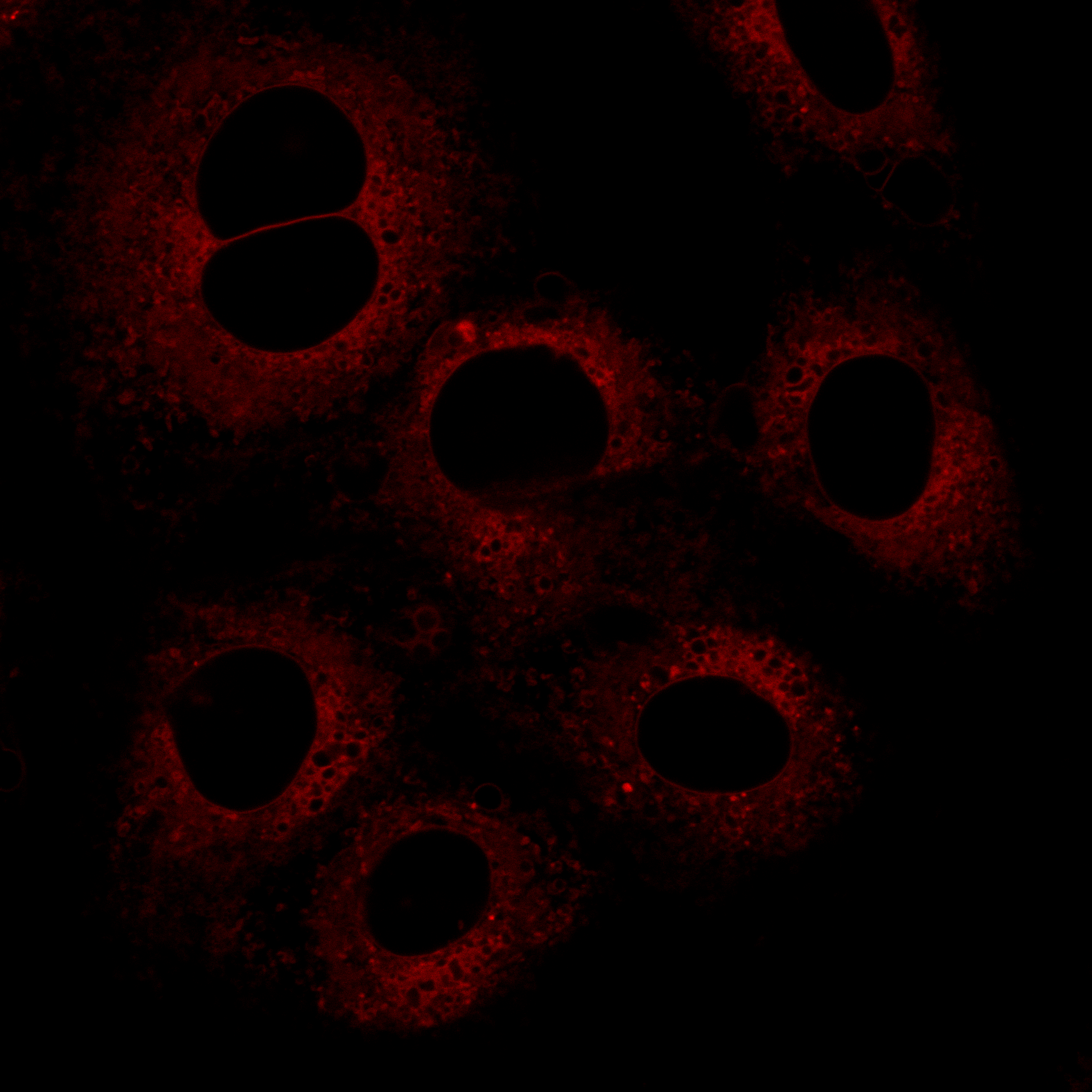

Supplement: Supplementary file 7 [file DataSheet10.ZIP › Fig 5D-E/Fig 5C/Control/Control-lipidTOX.tif]

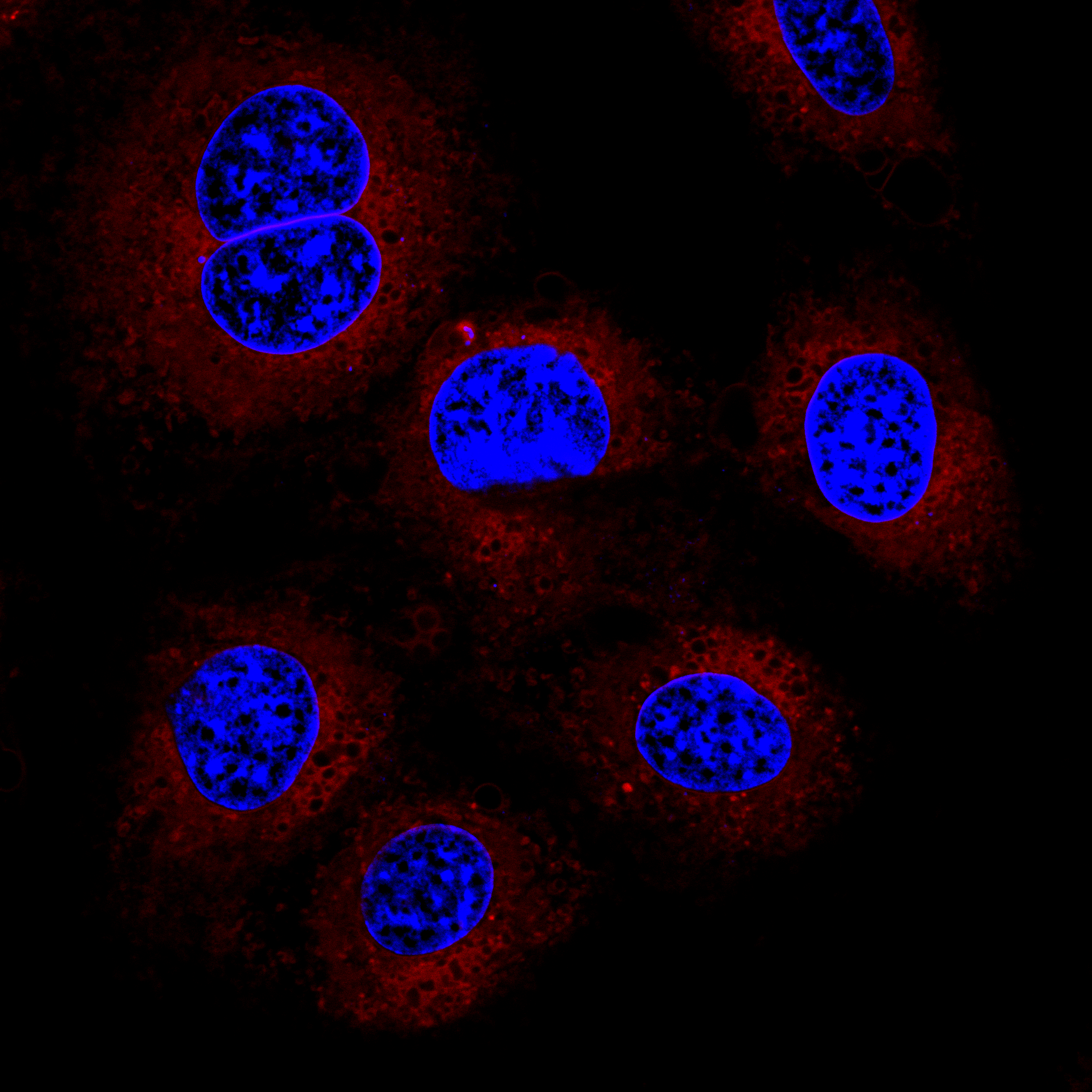

Supplement: Supplementary file 7 [file DataSheet10.ZIP › Fig 5D-E/Fig 5C/Control/Merge.tif]

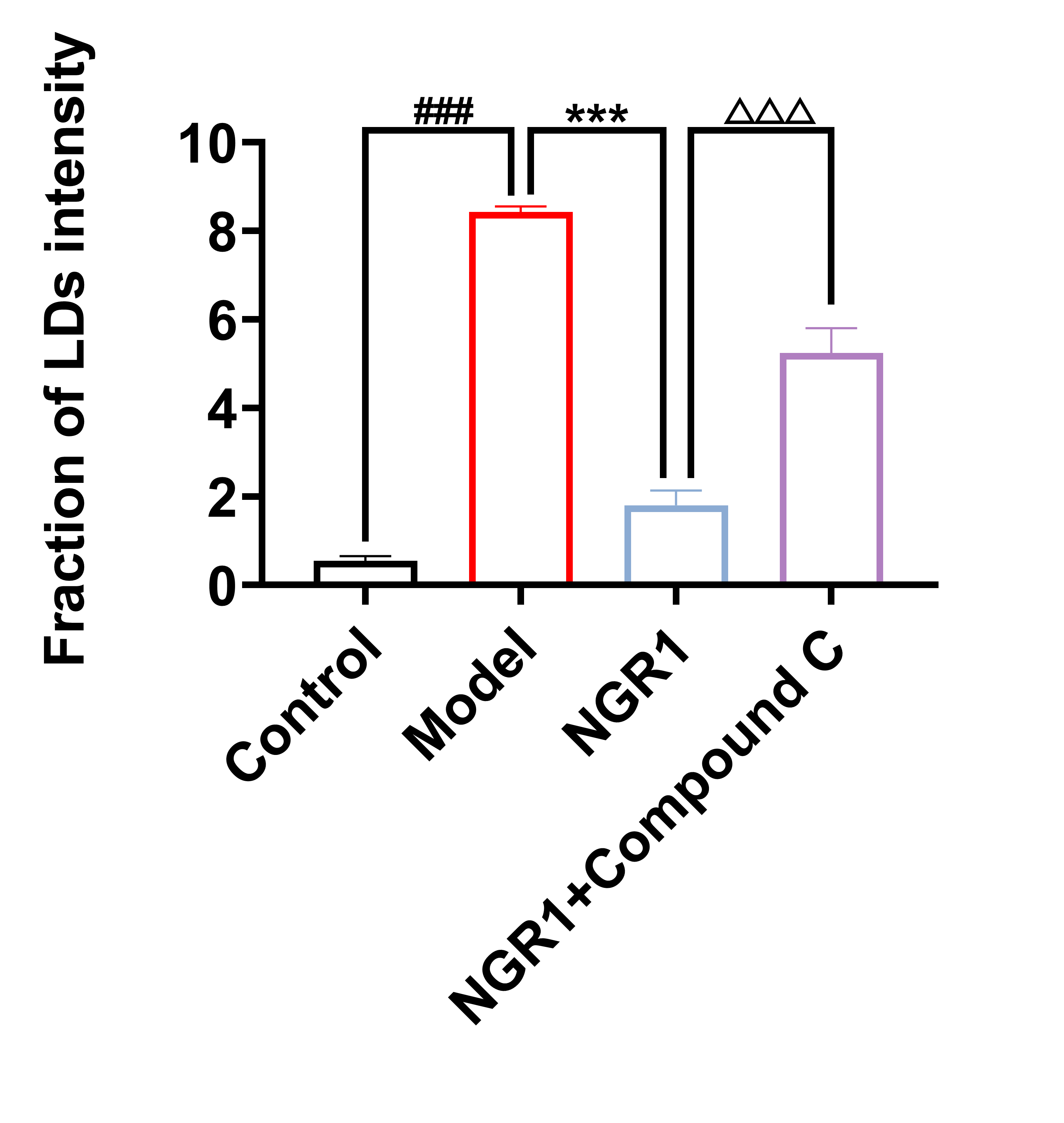

Supplement: Supplementary file 7 [file DataSheet10.ZIP › Fig 5D-E/Fig 5C/Fig 5C.tif]

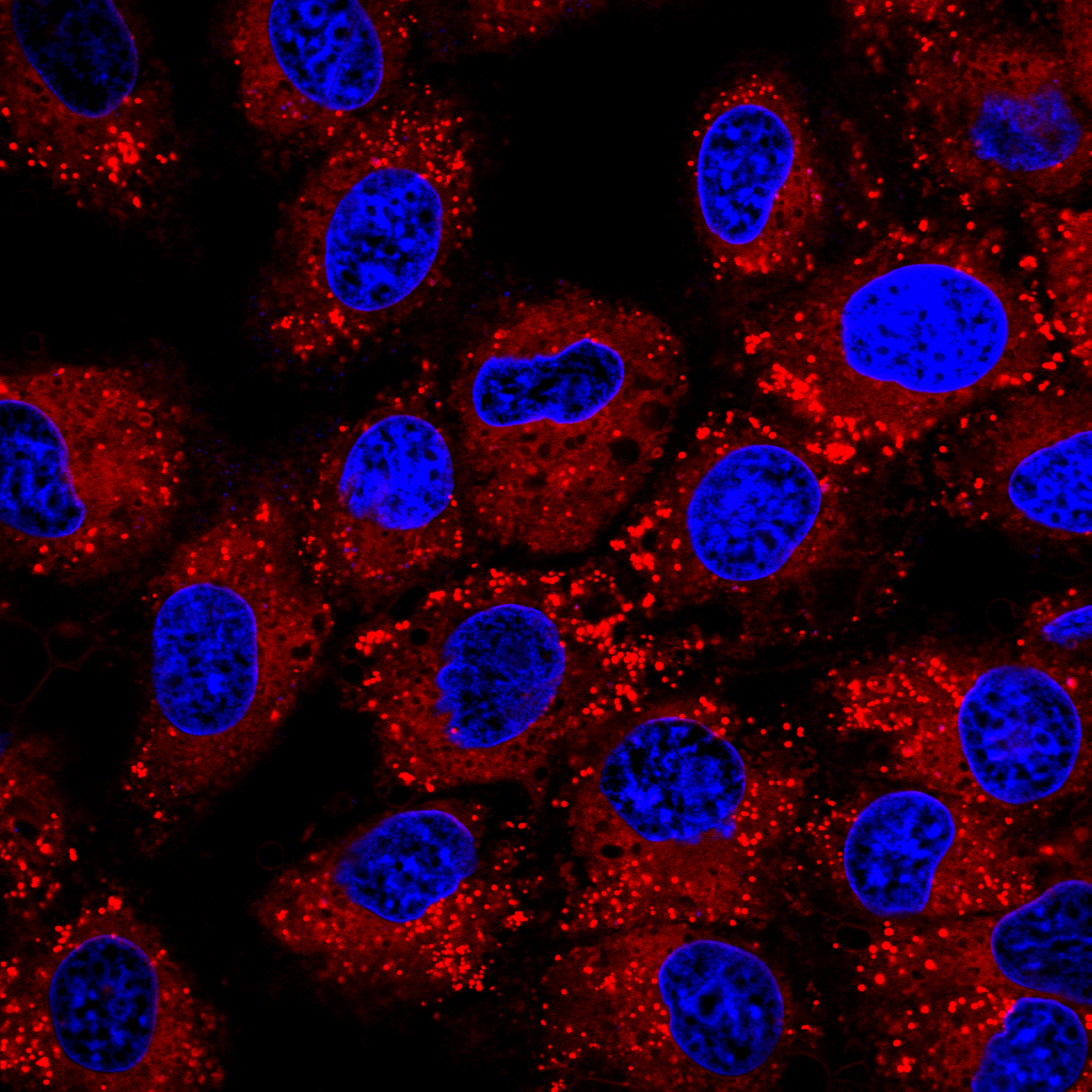

Supplement: Supplementary file 7 [file DataSheet10.ZIP › Fig 5D-E/Fig 5C/Model/Merge.tif]

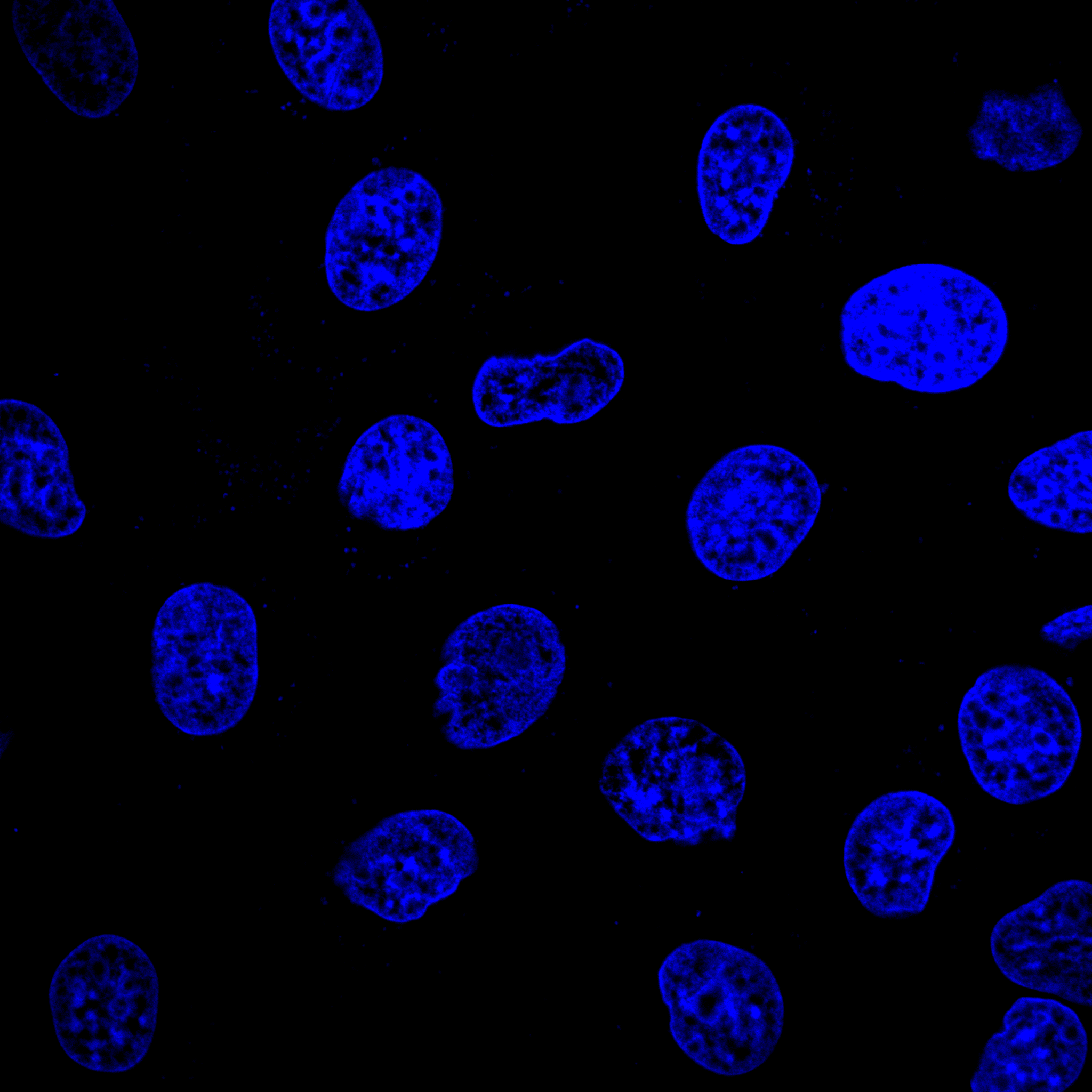

Supplement: Supplementary file 7 [file DataSheet10.ZIP › Fig 5D-E/Fig 5C/Model/Model-DAPI.tif]

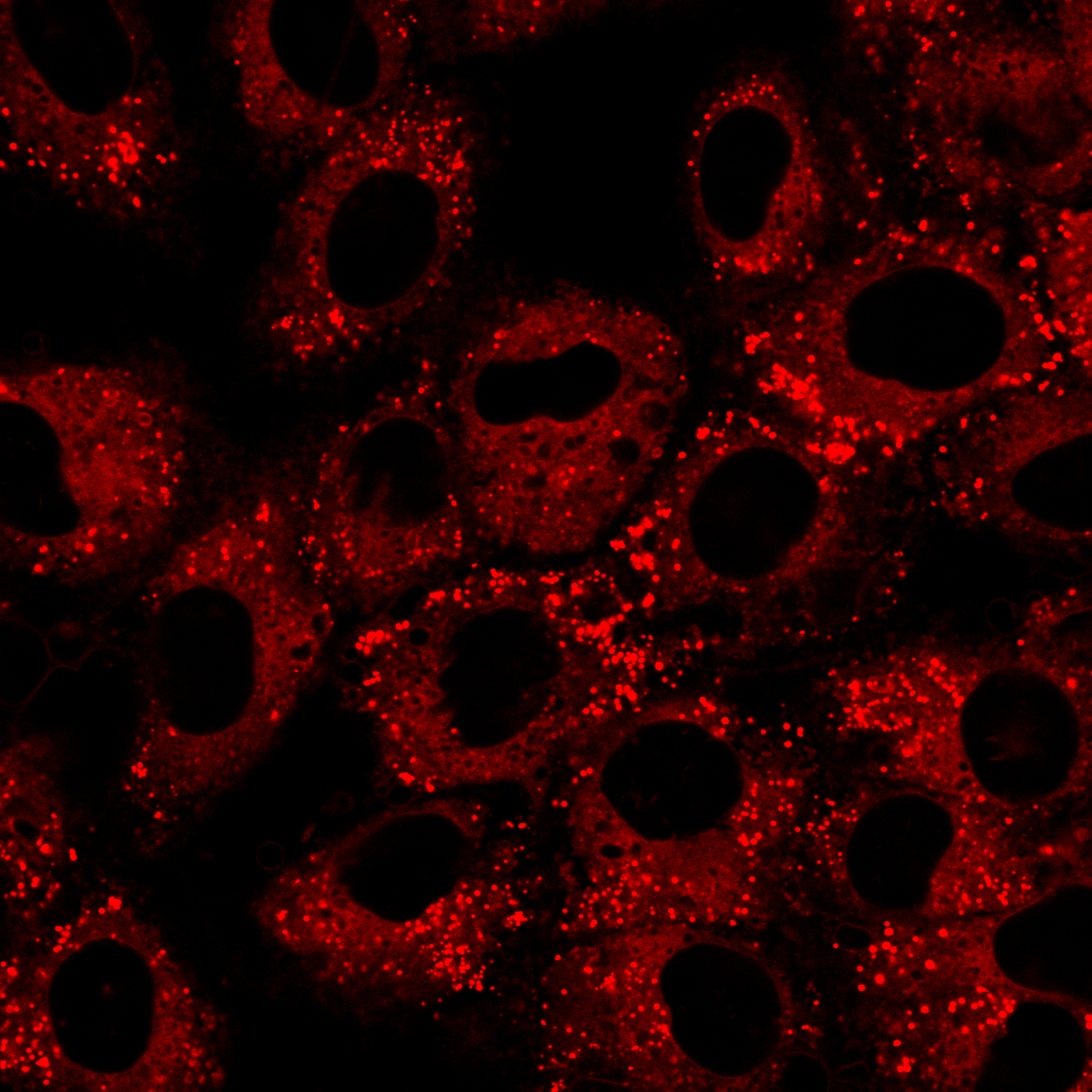

Supplement: Supplementary file 7 [file DataSheet10.ZIP › Fig 5D-E/Fig 5C/Model/Model-lipidTOX.tif]

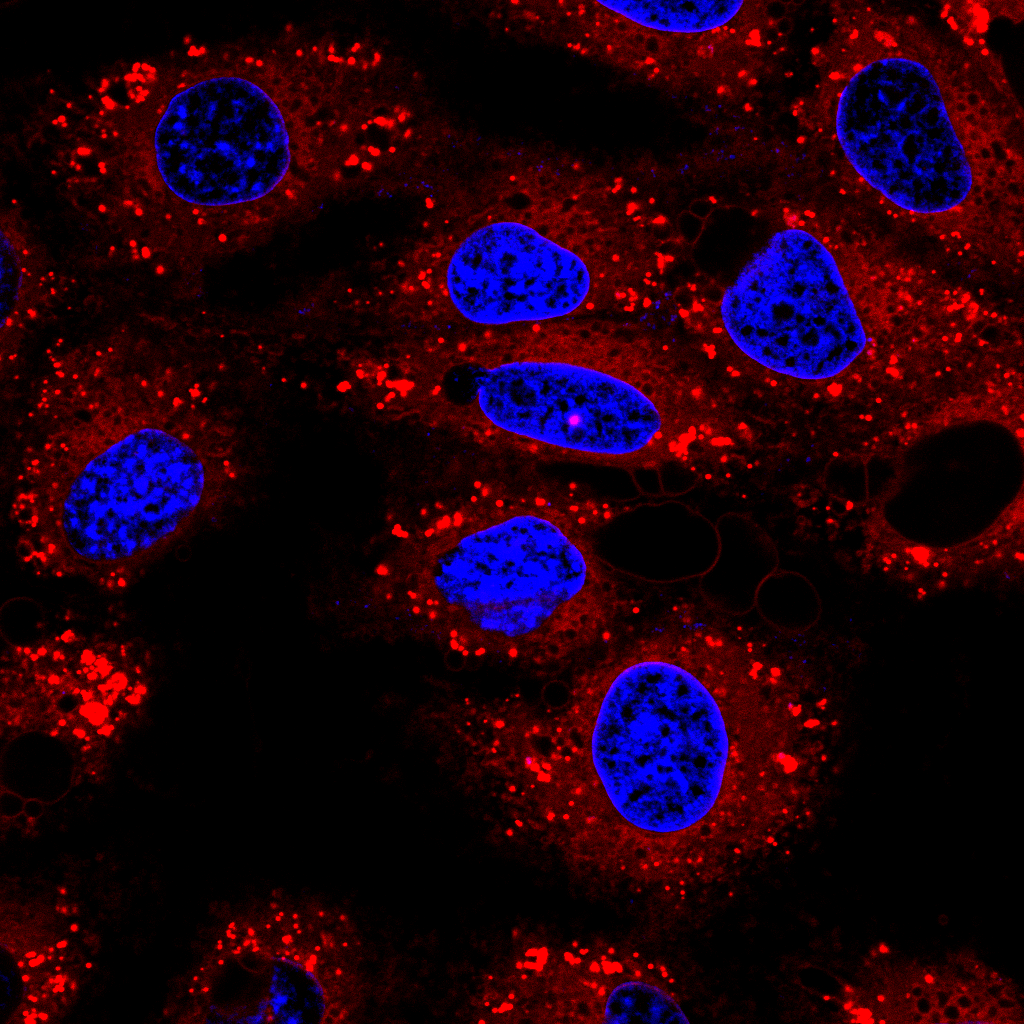

Supplement: Supplementary file 7 [file DataSheet10.ZIP › Fig 5D-E/Fig 5C/NGR1+Compound C/Merge.tif]

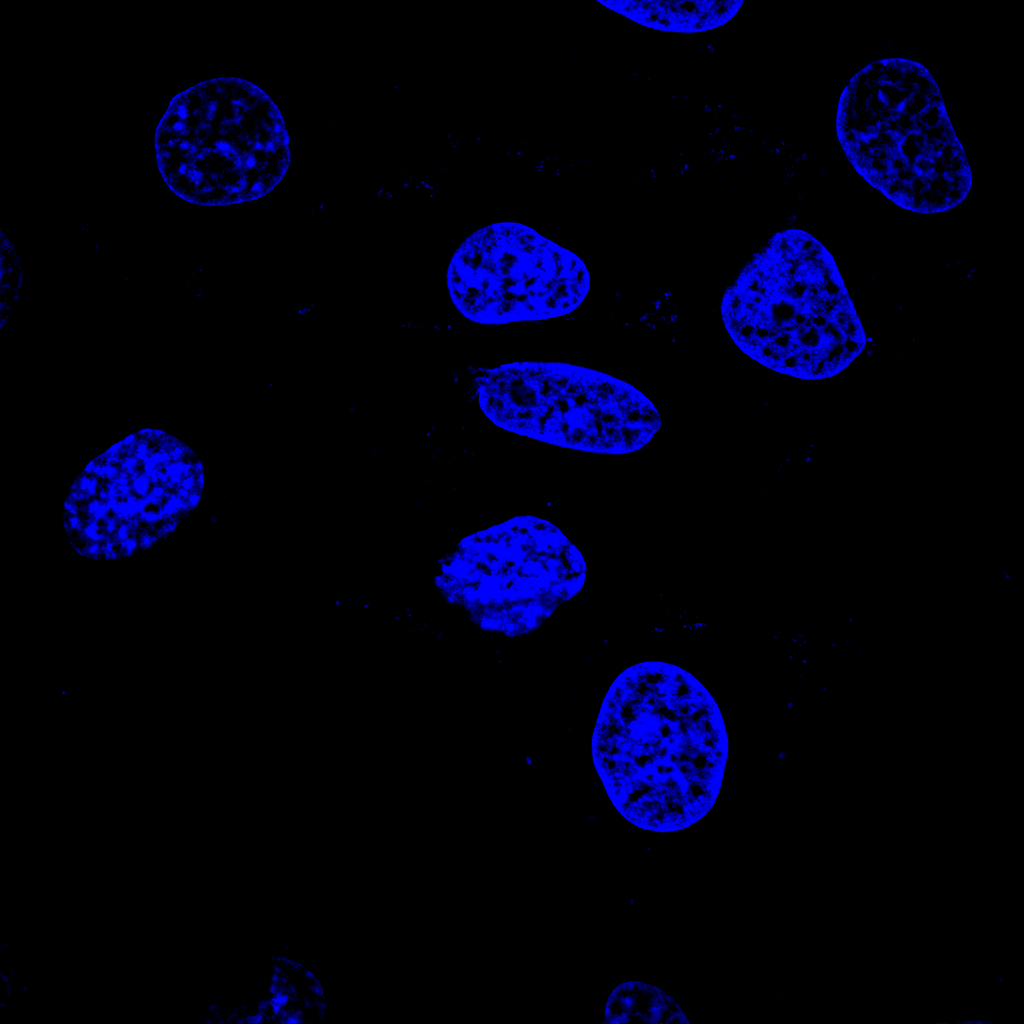

Supplement: Supplementary file 7 [file DataSheet10.ZIP › Fig 5D-E/Fig 5C/NGR1+Compound C/NGR1+Compound C-DAPI.tif]

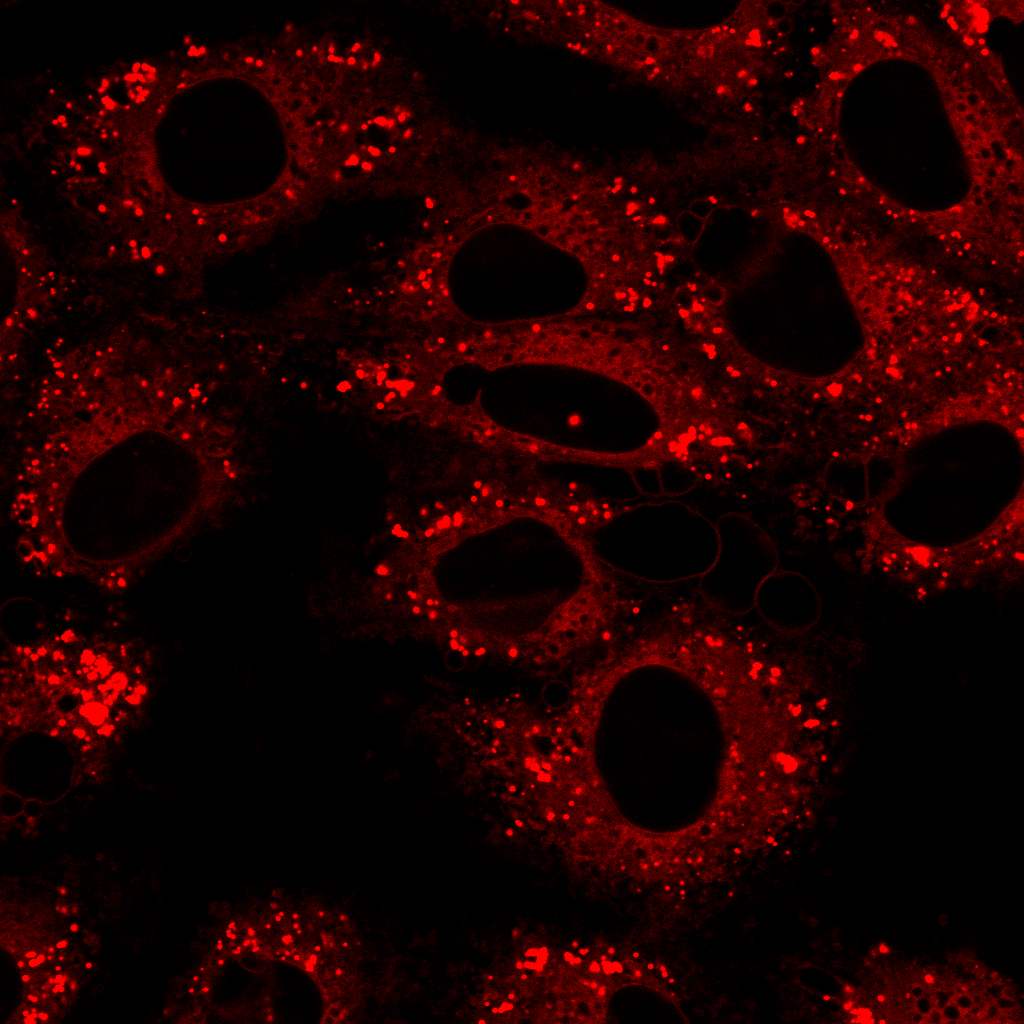

Supplement: Supplementary file 7 [file DataSheet10.ZIP › Fig 5D-E/Fig 5C/NGR1+Compound C/NGR1+Compound C-lipidTOX.tif]

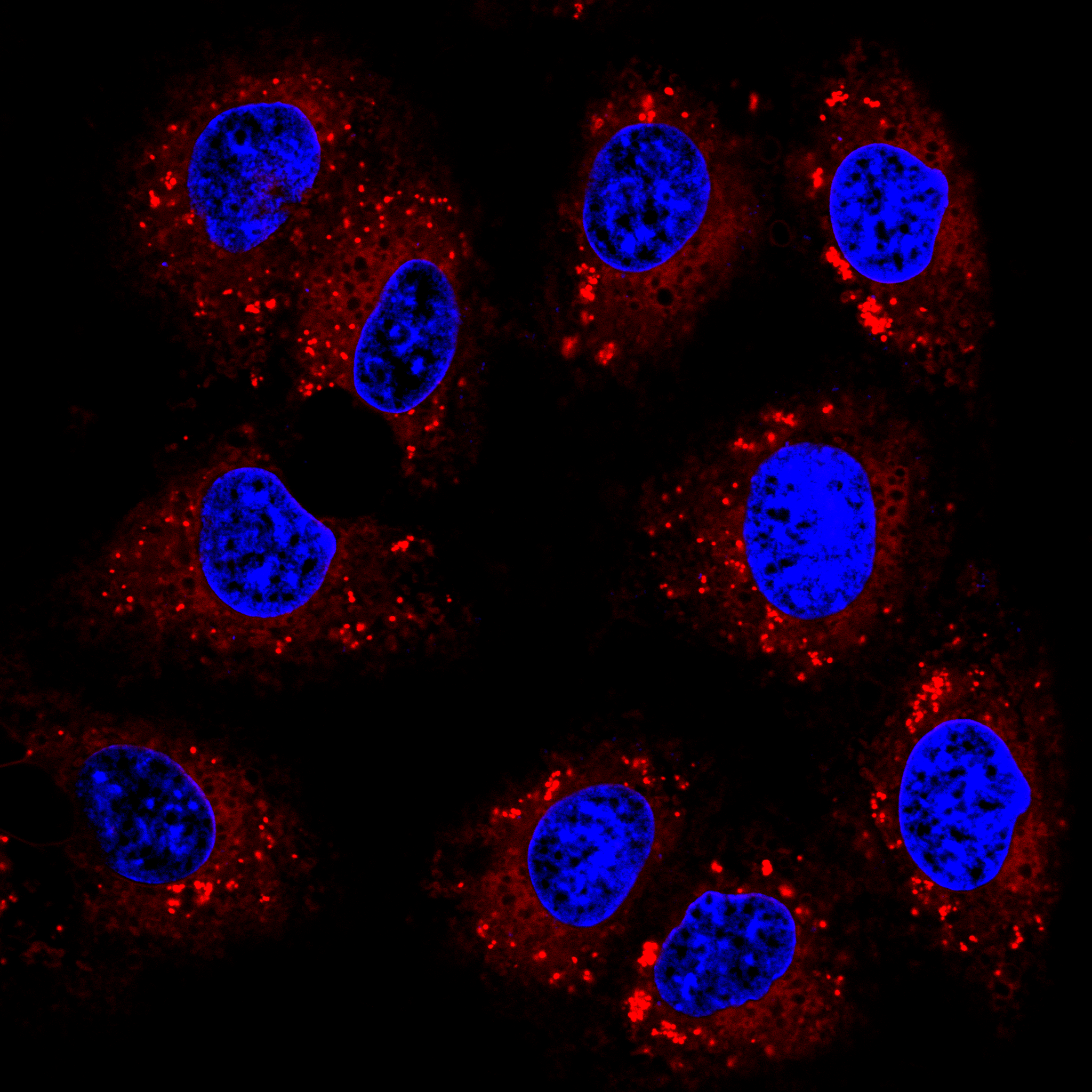

Supplement: Supplementary file 7 [file DataSheet10.ZIP › Fig 5D-E/Fig 5C/NGR1/Merge.tif]

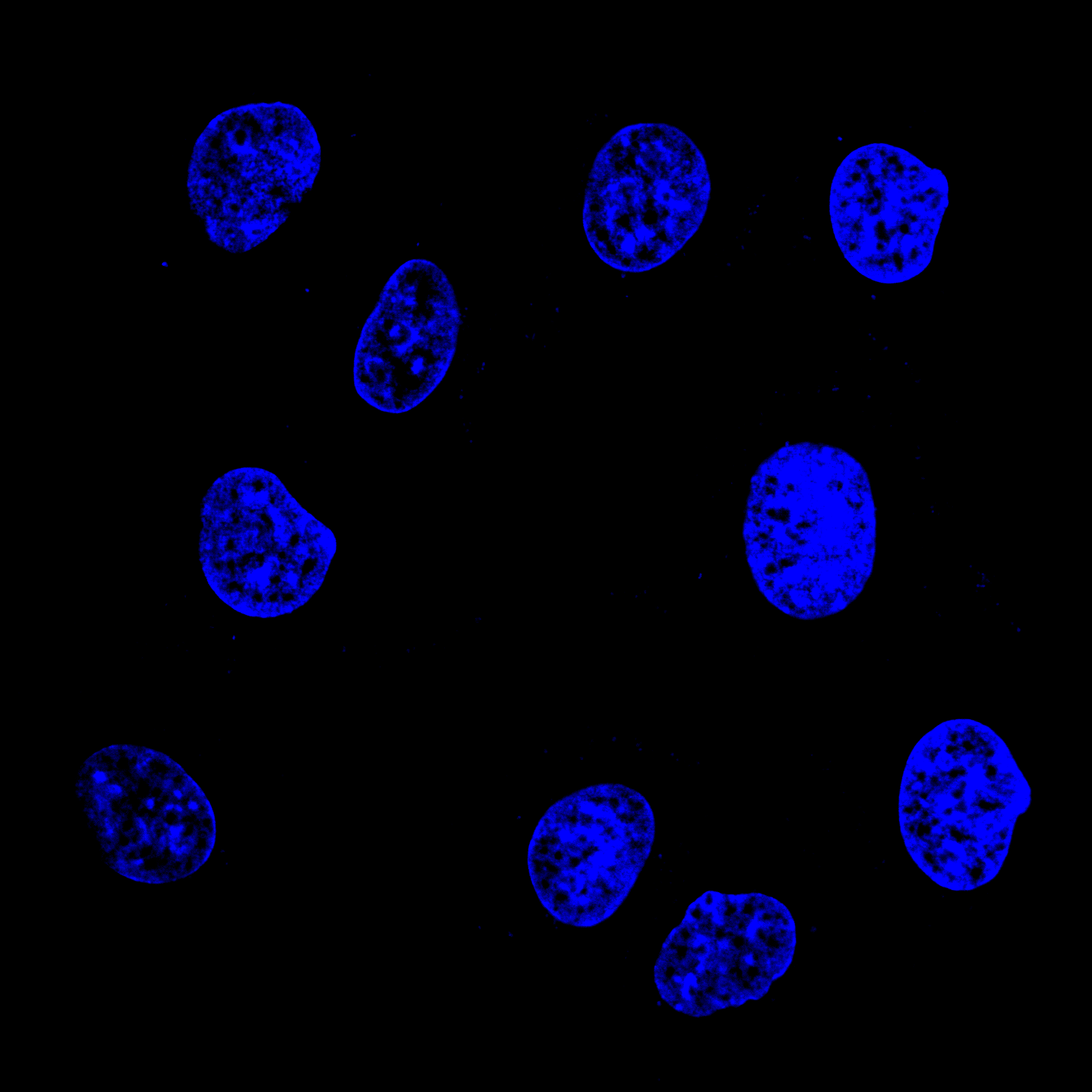

Supplement: Supplementary file 7 [file DataSheet10.ZIP › Fig 5D-E/Fig 5C/NGR1/NGR1-DAPI.tif]

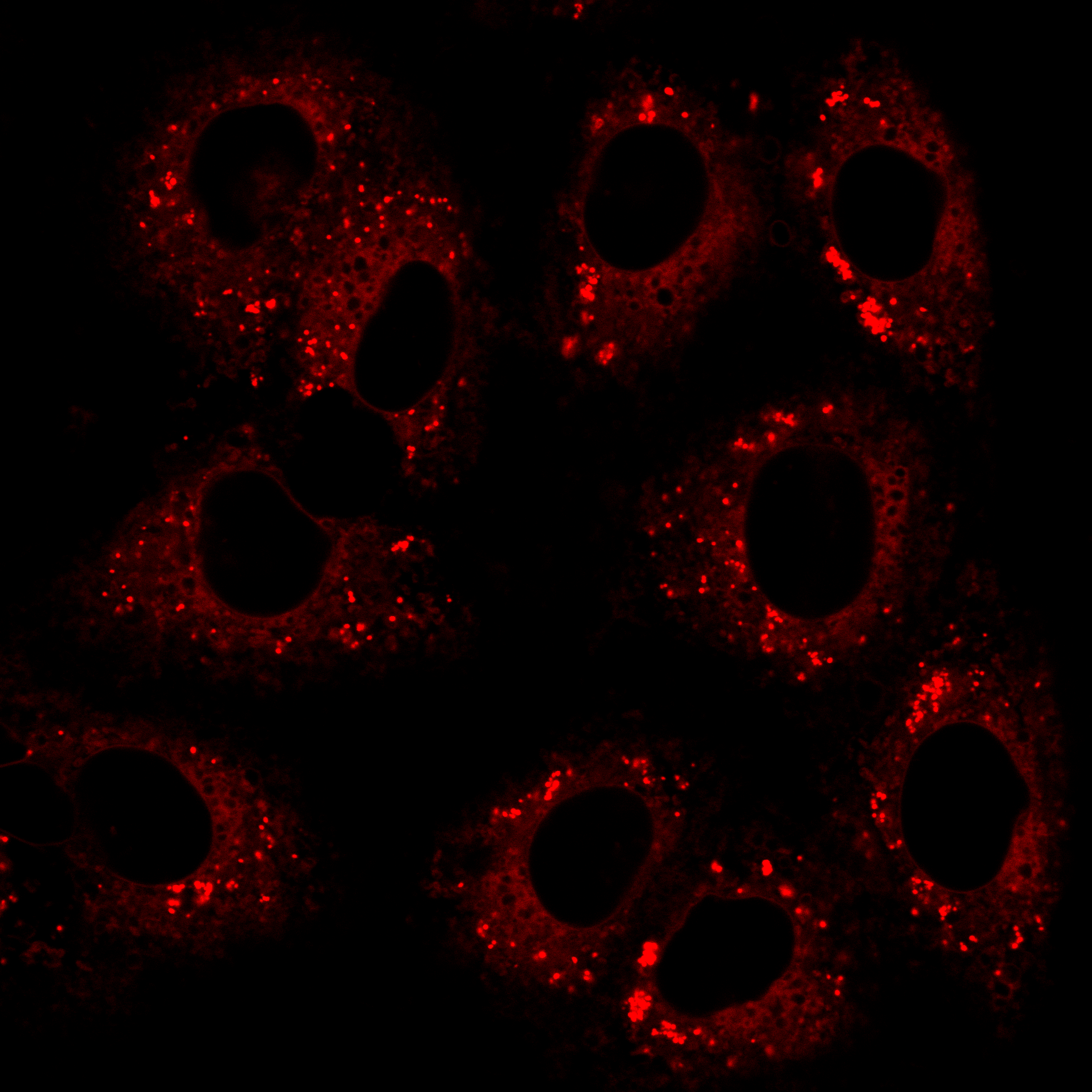

Supplement: Supplementary file 7 [file DataSheet10.ZIP › Fig 5D-E/Fig 5C/NGR1/NGR1-LipidTOX.tif]

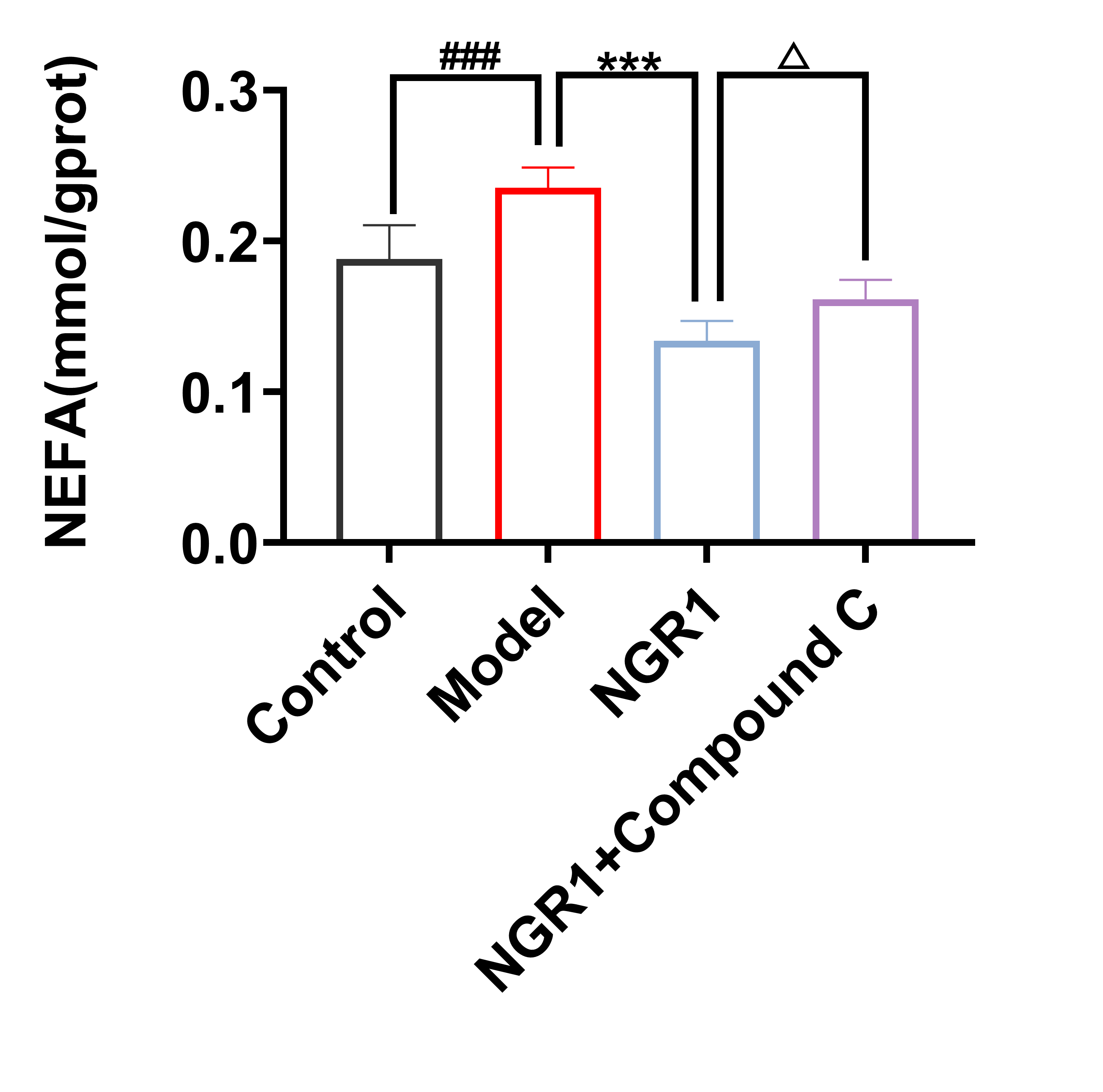

Supplement: Supplementary file 7 [file DataSheet10.ZIP › Fig 5D-E/NEFA.tif]

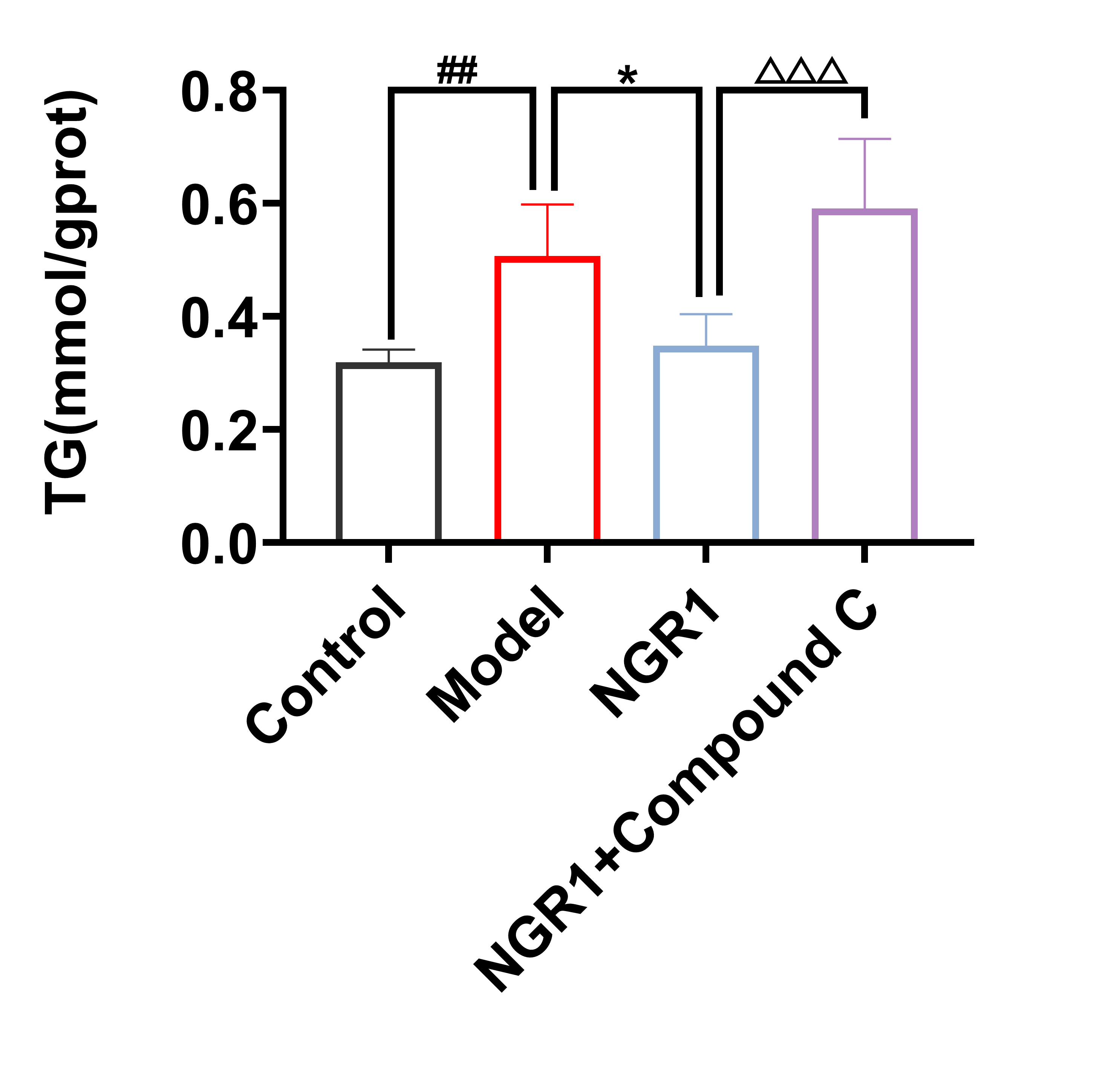

Supplement: Supplementary file 7 [file DataSheet10.ZIP › Fig 5D-E/TG.tif]

## Slide 1
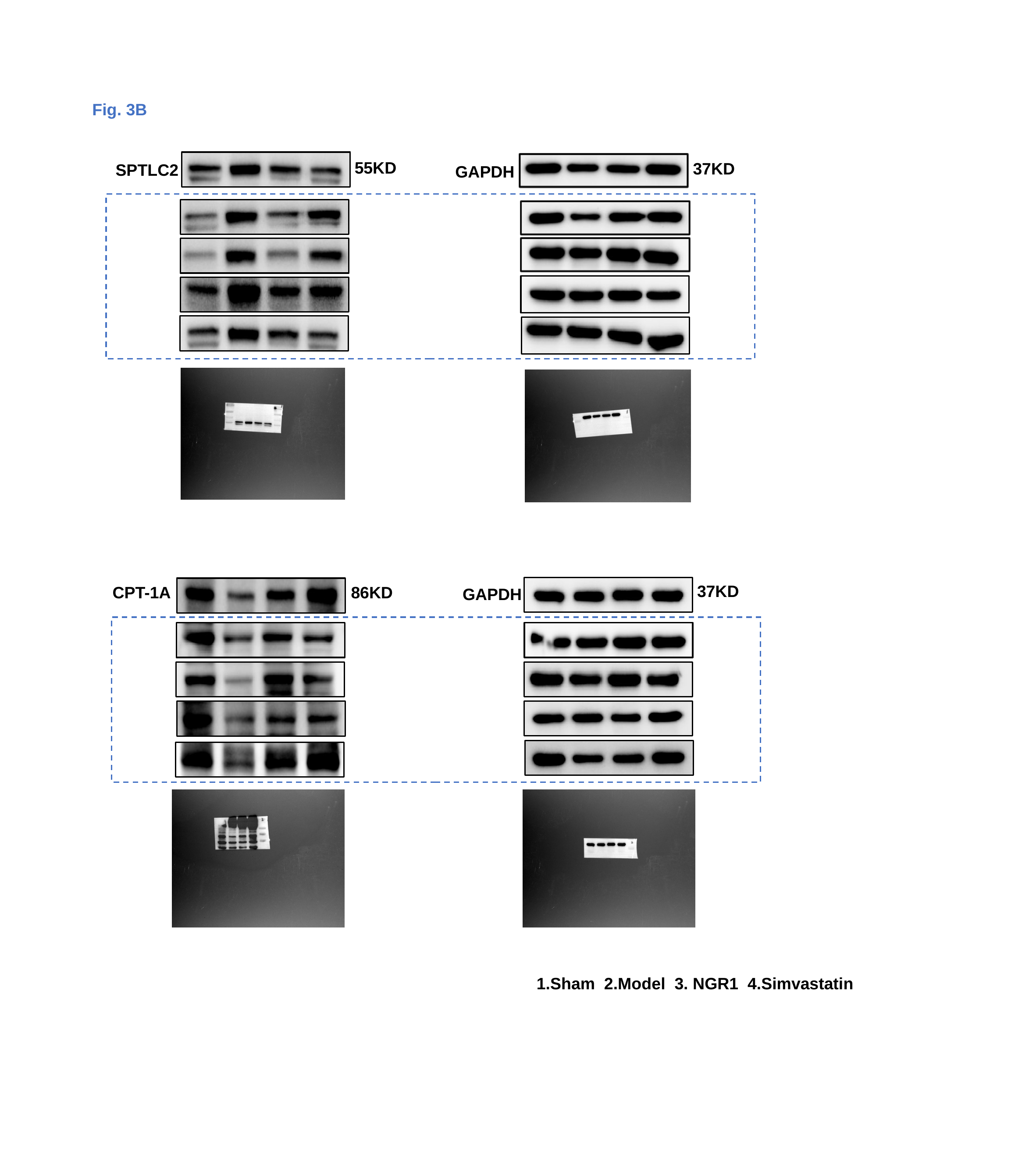

Fig. 3B
55KD
SPTLC2
37KD
GAPDH
37KD
GAPDH
CPT-1A
86KD
1.Sham 2.Model 3. NGR1 4.Simvastatin

Supplement: Supplementary file 8 [file DataSheet6.ZIP › Figure 3(3).pptx]

## Slide 1
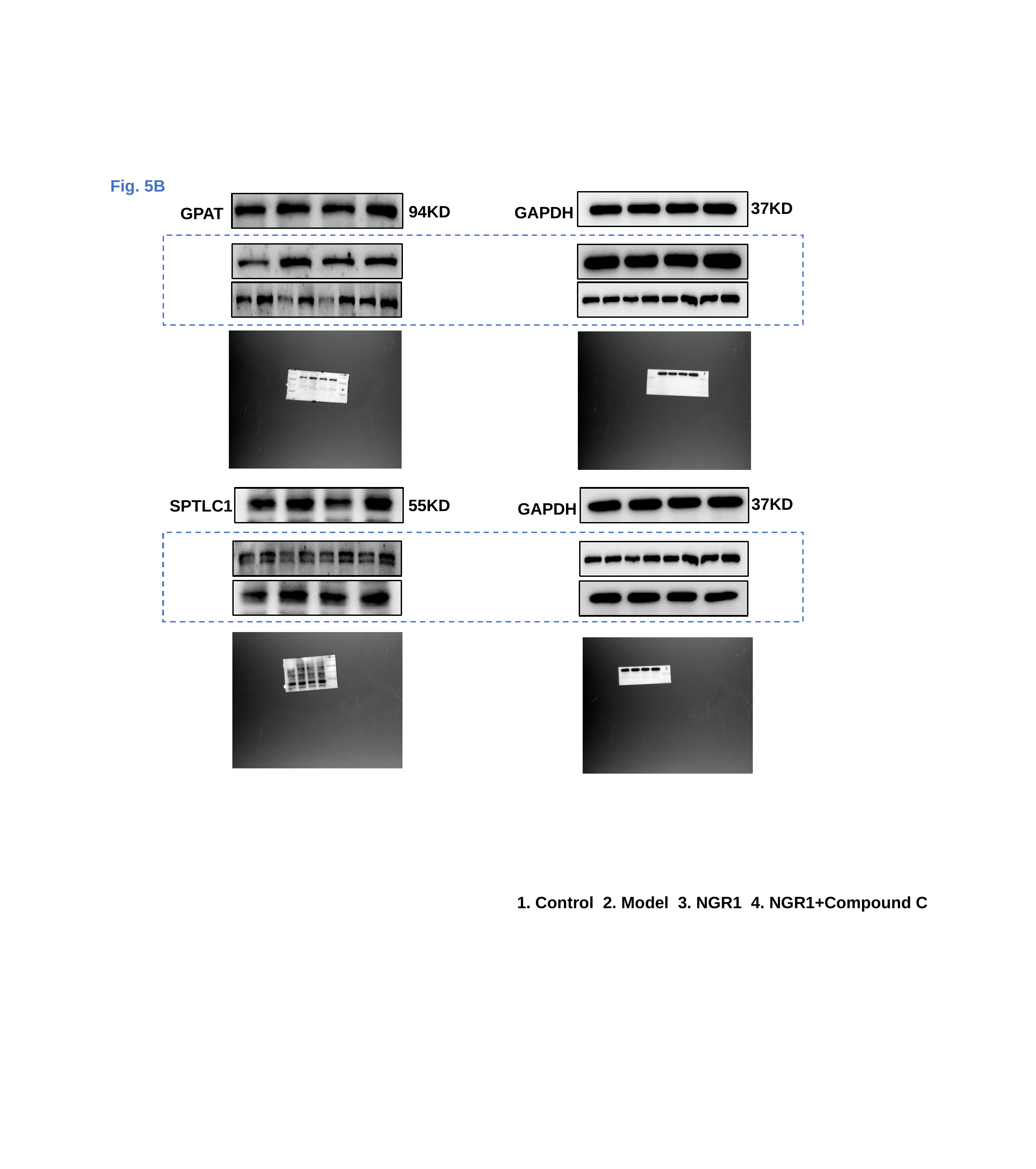

Fig. 5B
37KD
GAPDH
94KD
GPAT
55KD
SPTLC1
37KD
GAPDH
1. Control 2. Model 3. NGR1 4. NGR1+Compound C

Supplement: Supplementary file 9 [file DataSheet12.ZIP › Figure 5B.pptx]

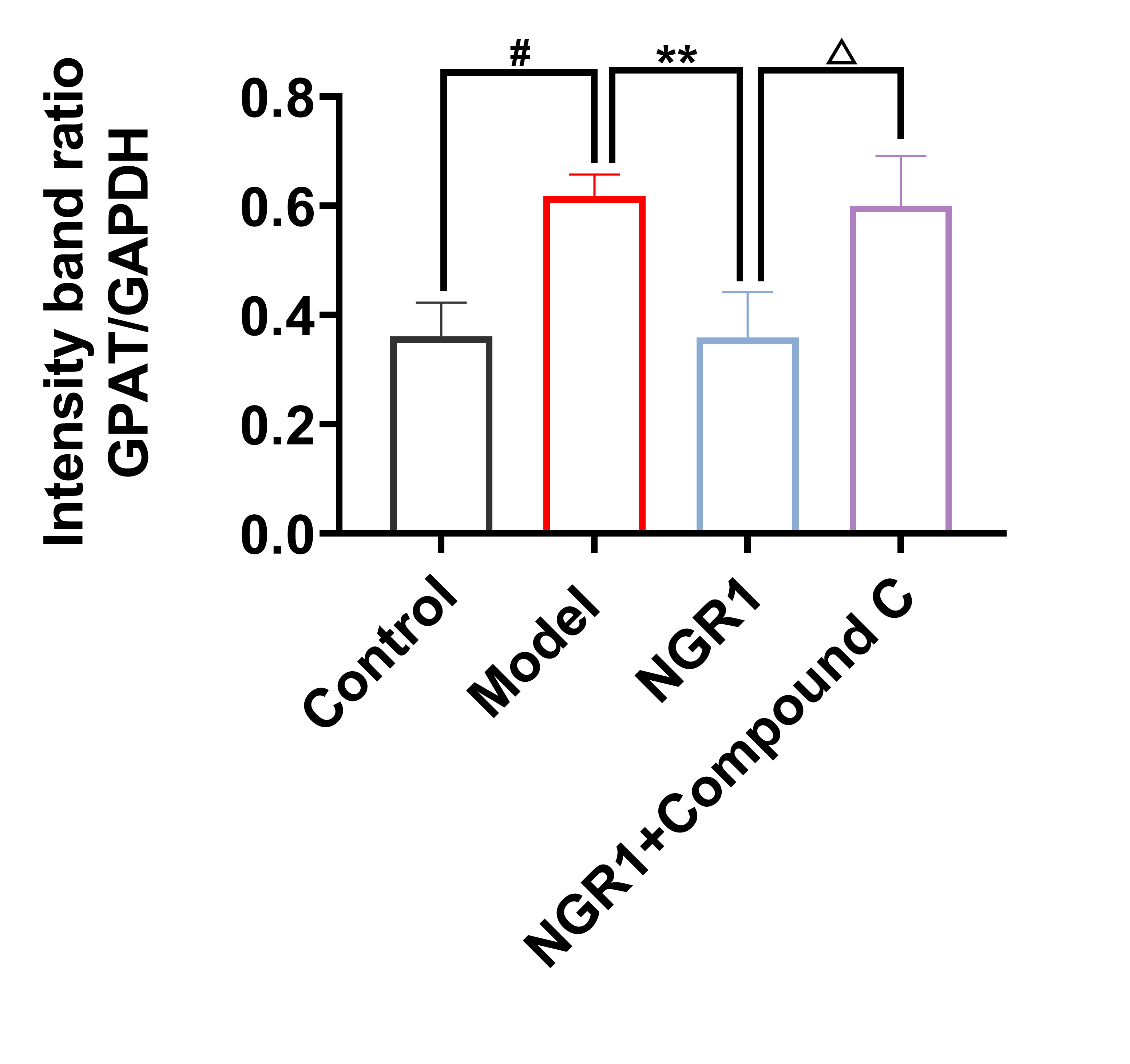

Supplement: Supplementary file 9 [file DataSheet12.ZIP › GPAT.tif]

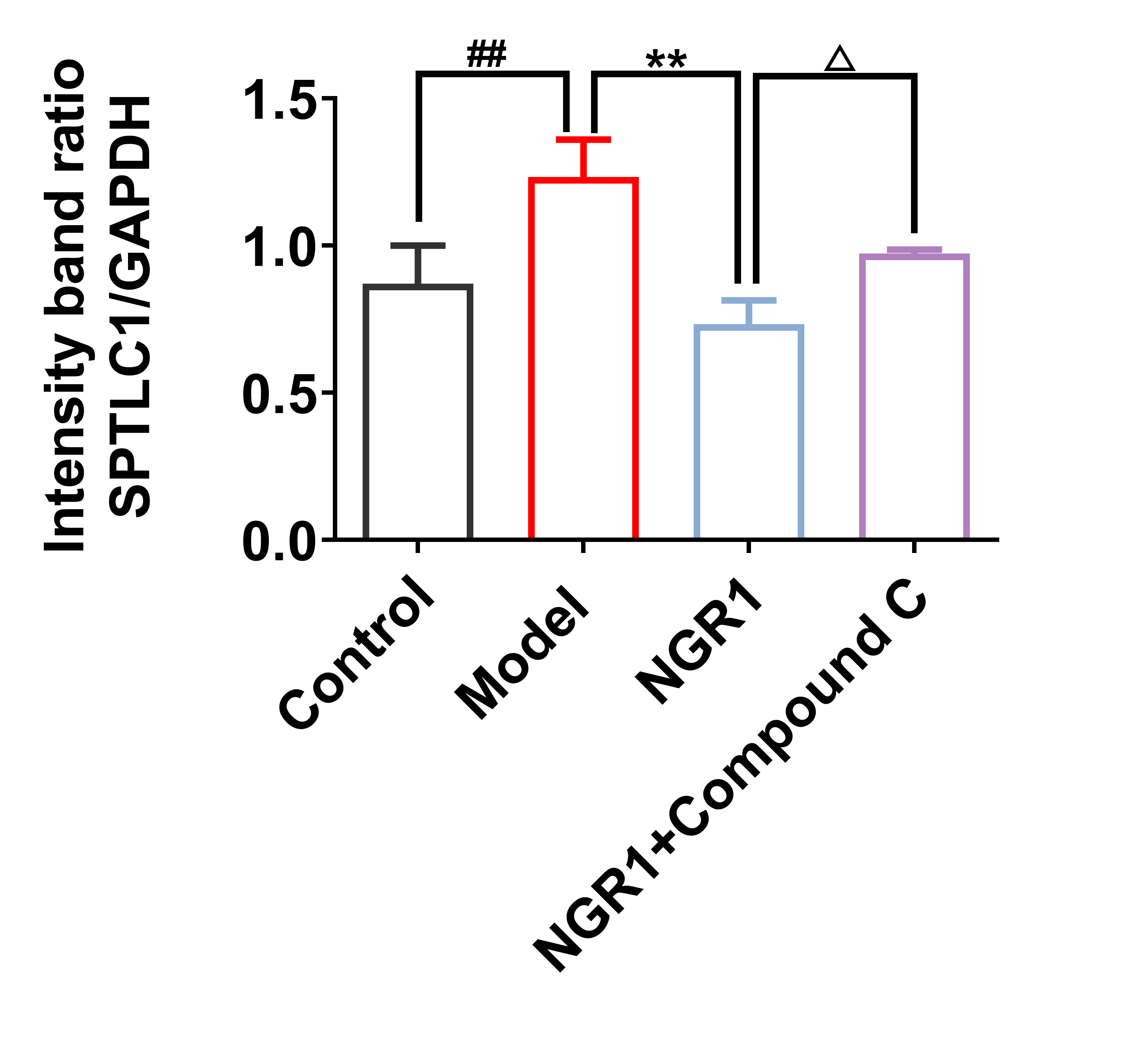

Supplement: Supplementary file 9 [file DataSheet12.ZIP › SPTLC1.tif]

## Slide 1
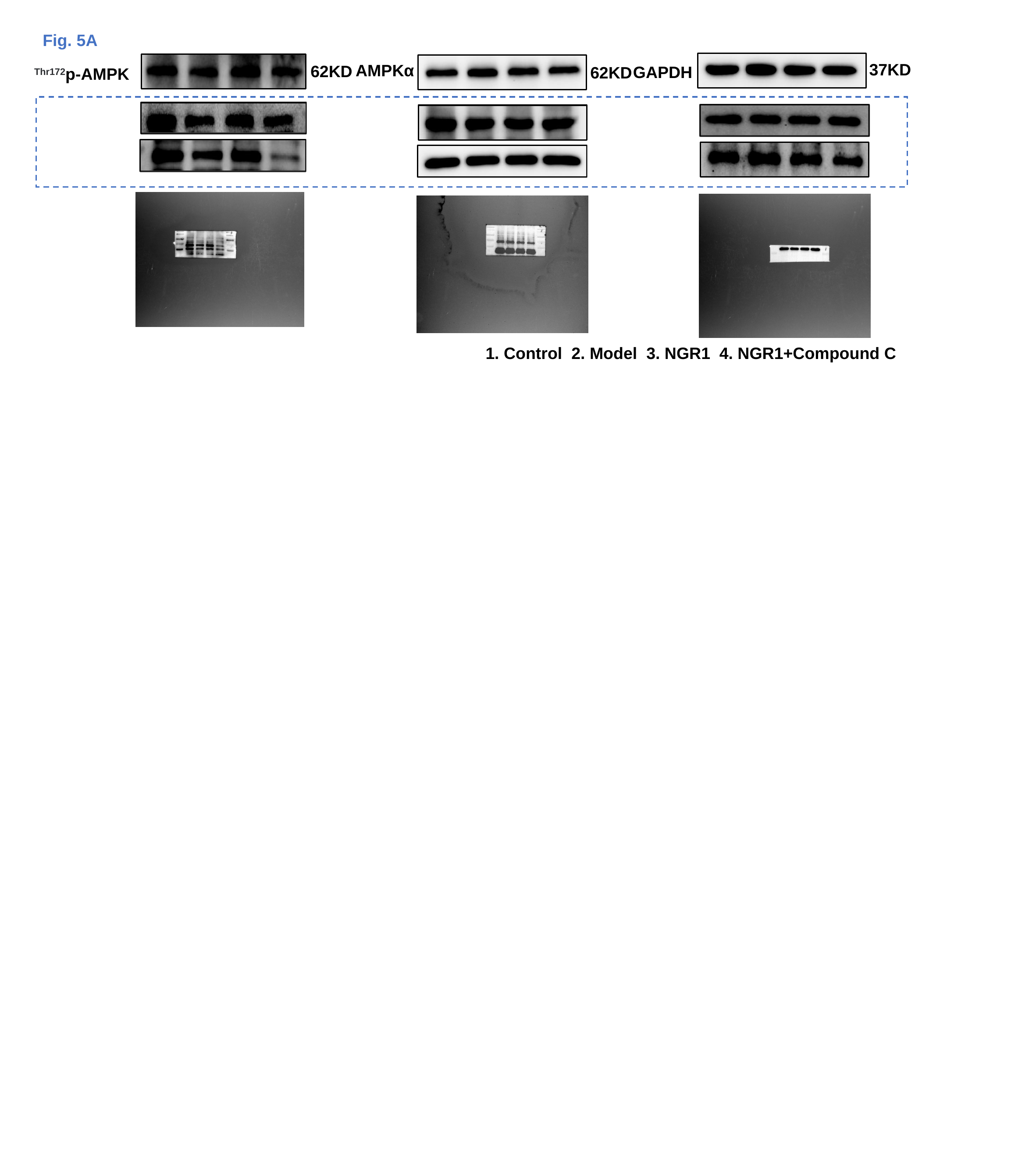

Fig. 5A
37KD
GAPDH
62KD
Thr172p-AMPK
AMPKα
62KD
1. Control 2. Model 3. NGR1 4. NGR1+Compound C

Supplement: Supplementary file 10 [file DataSheet2.ZIP › Figure 5A.pptx]

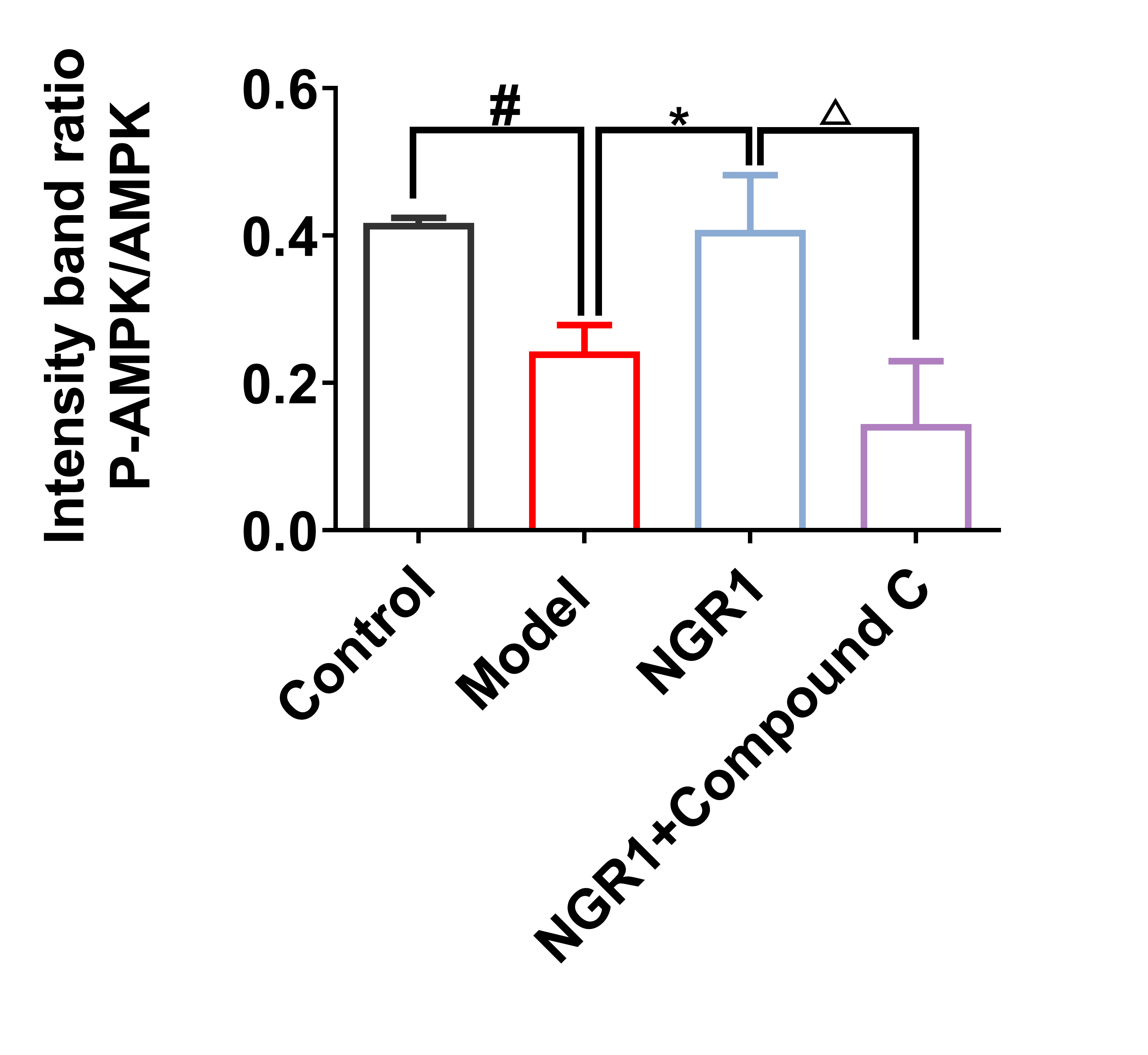

Supplement: Supplementary file 10 [file DataSheet2.ZIP › p-AMPK.tif]

## Slide 1
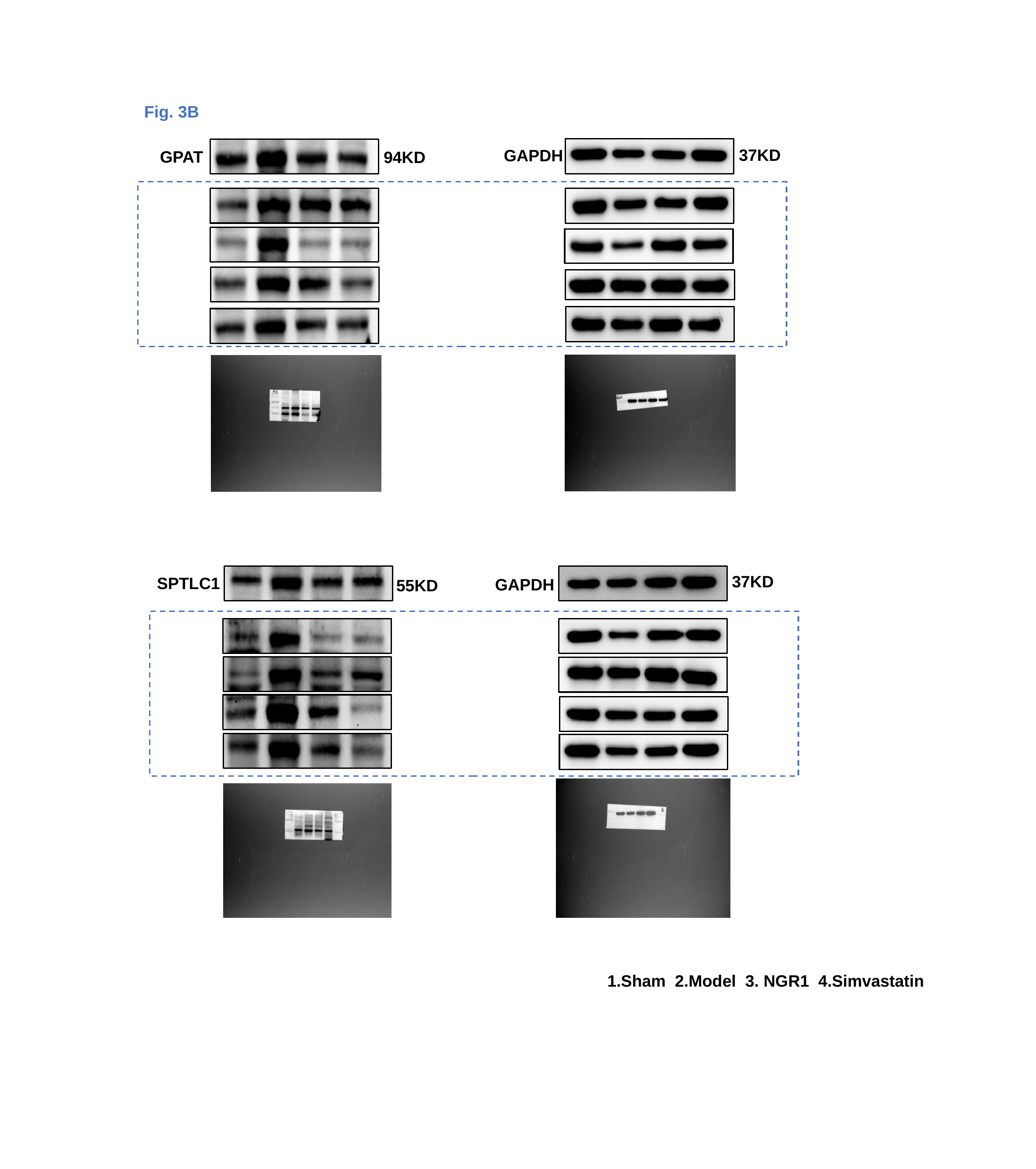

Fig. 3B
37KD
GAPDH
GPAT
94KD
SPTLC1
55KD
37KD
GAPDH
1.Sham 2.Model 3. NGR1 4.Simvastatin

Supplement: Supplementary file 11 [file DataSheet5.ZIP › Figure 3(2).pptx]

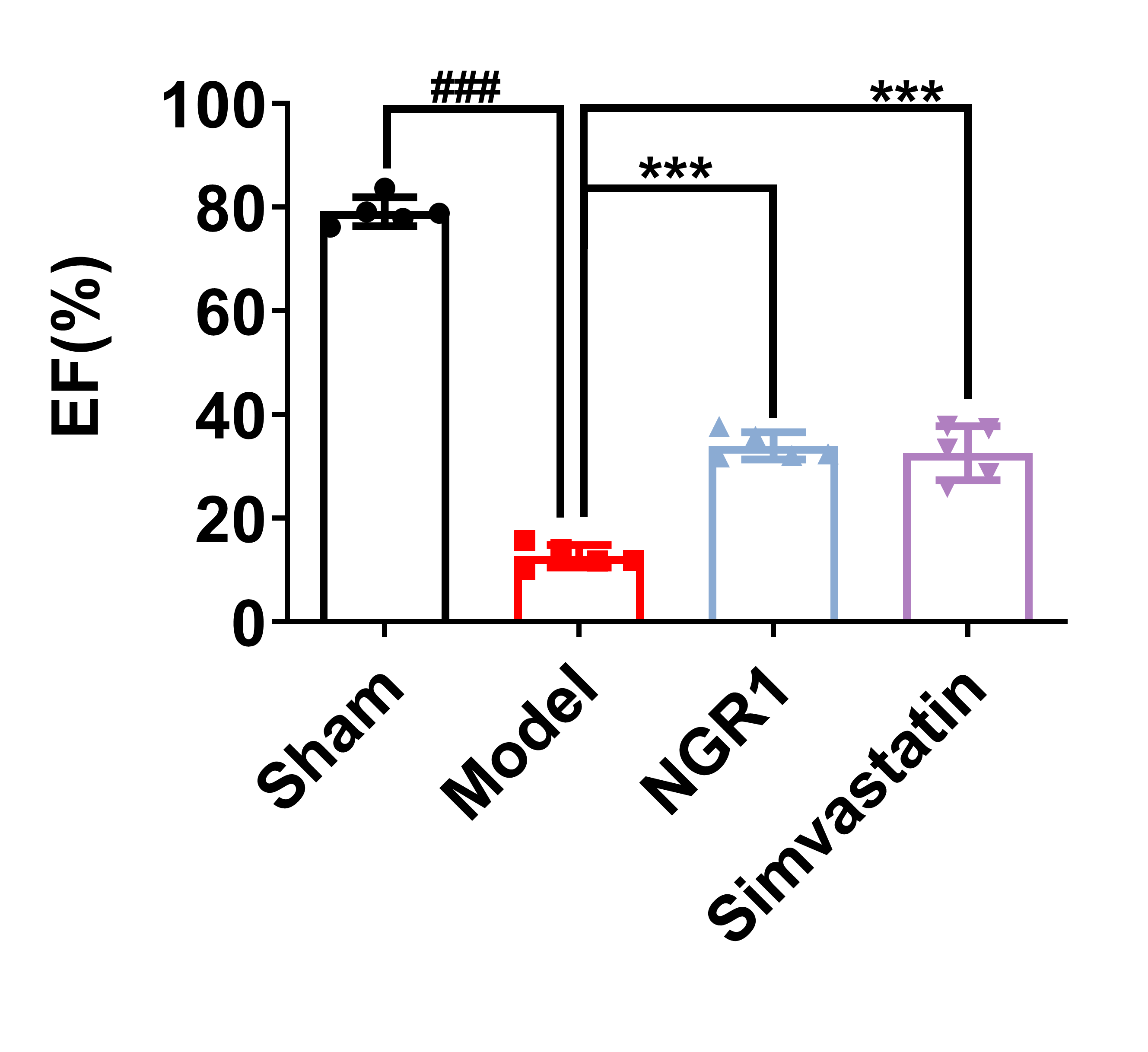

Supplement: Supplementary file 12 [file DataSheet7.ZIP › Fig 1A-B/EF.tif]

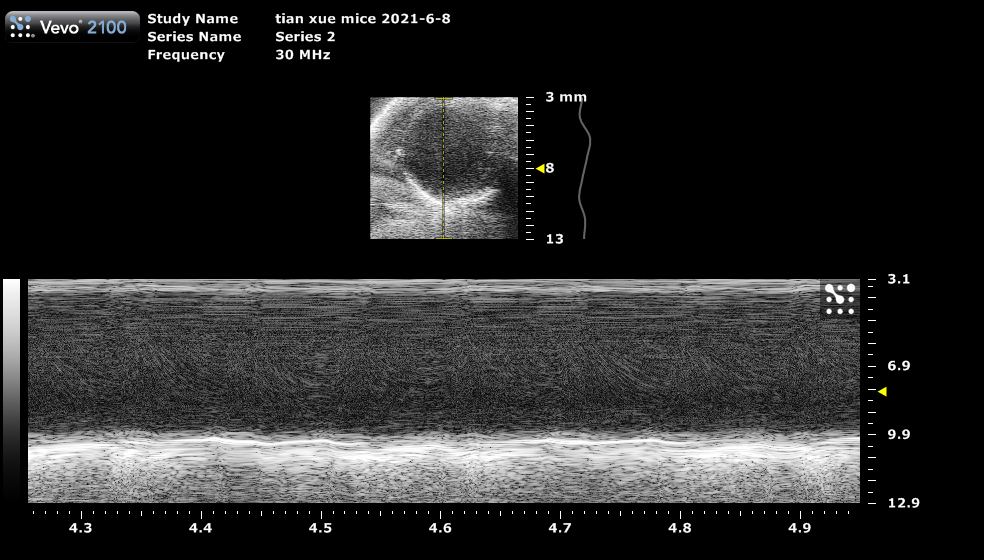

Supplement: Supplementary file 12 [file DataSheet7.ZIP › Fig 1A-B/Fig 1A-Model.tif]

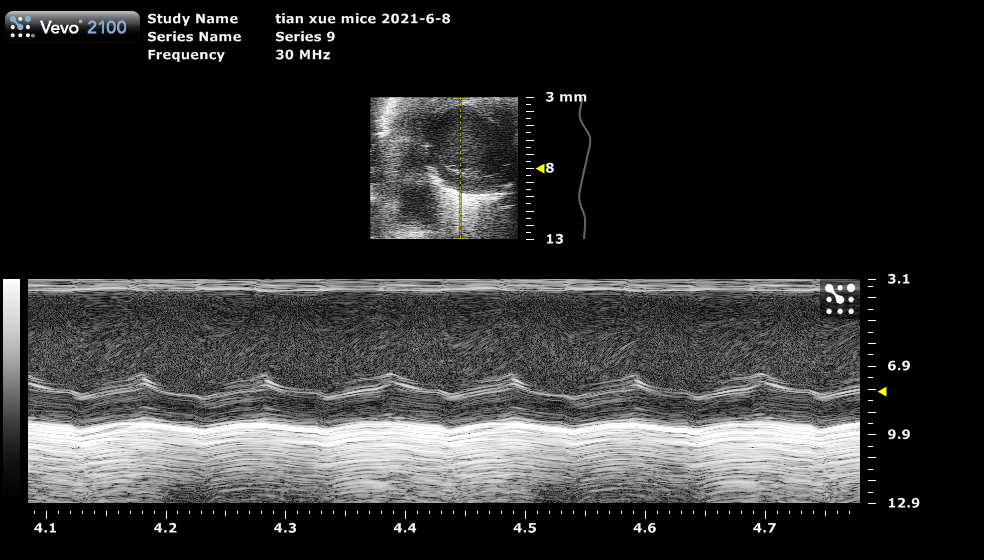

Supplement: Supplementary file 12 [file DataSheet7.ZIP › Fig 1A-B/Fig 1A-NGR1.tif]

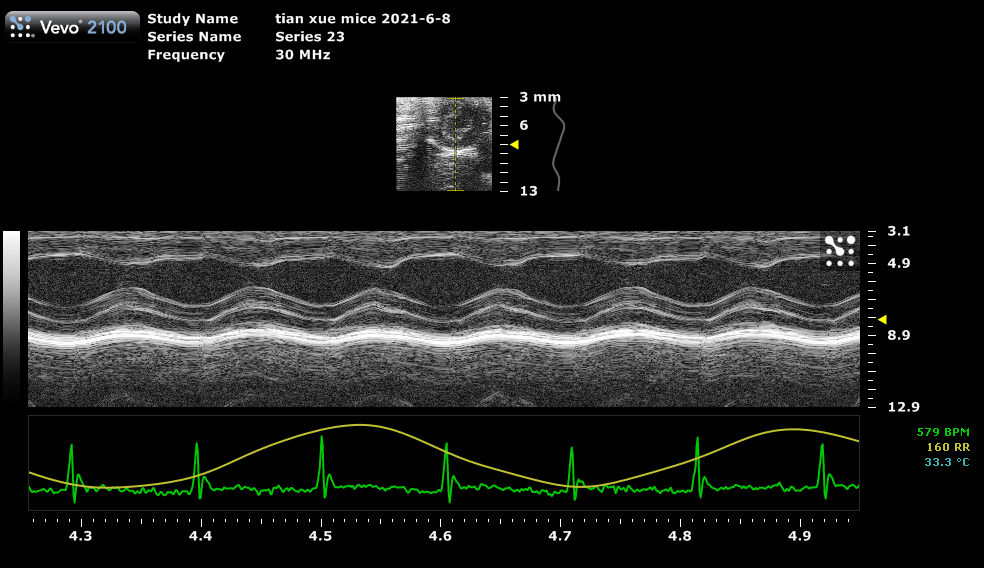

Supplement: Supplementary file 12 [file DataSheet7.ZIP › Fig 1A-B/Fig 1A-Sham.tif]

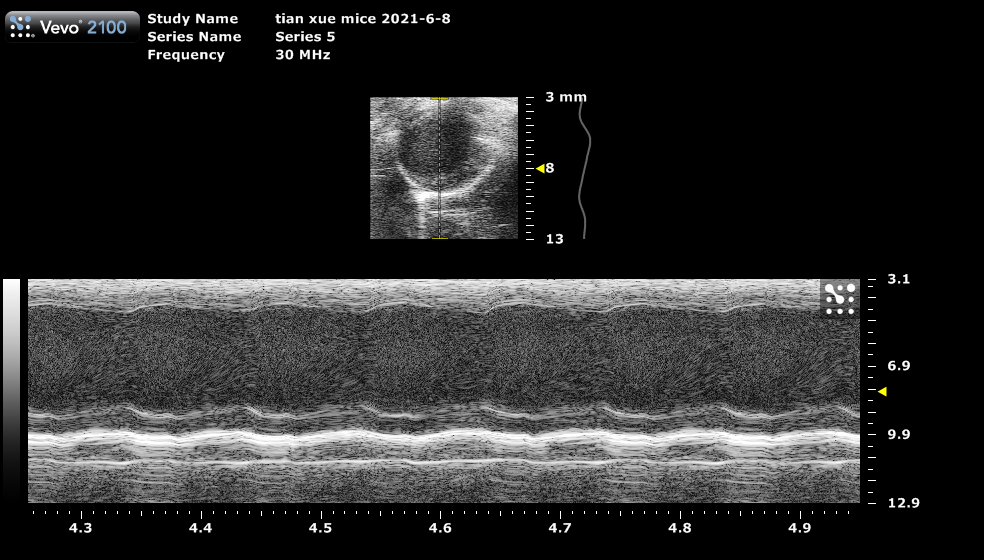

Supplement: Supplementary file 12 [file DataSheet7.ZIP › Fig 1A-B/Fig 1A-Simvastatin.tif]

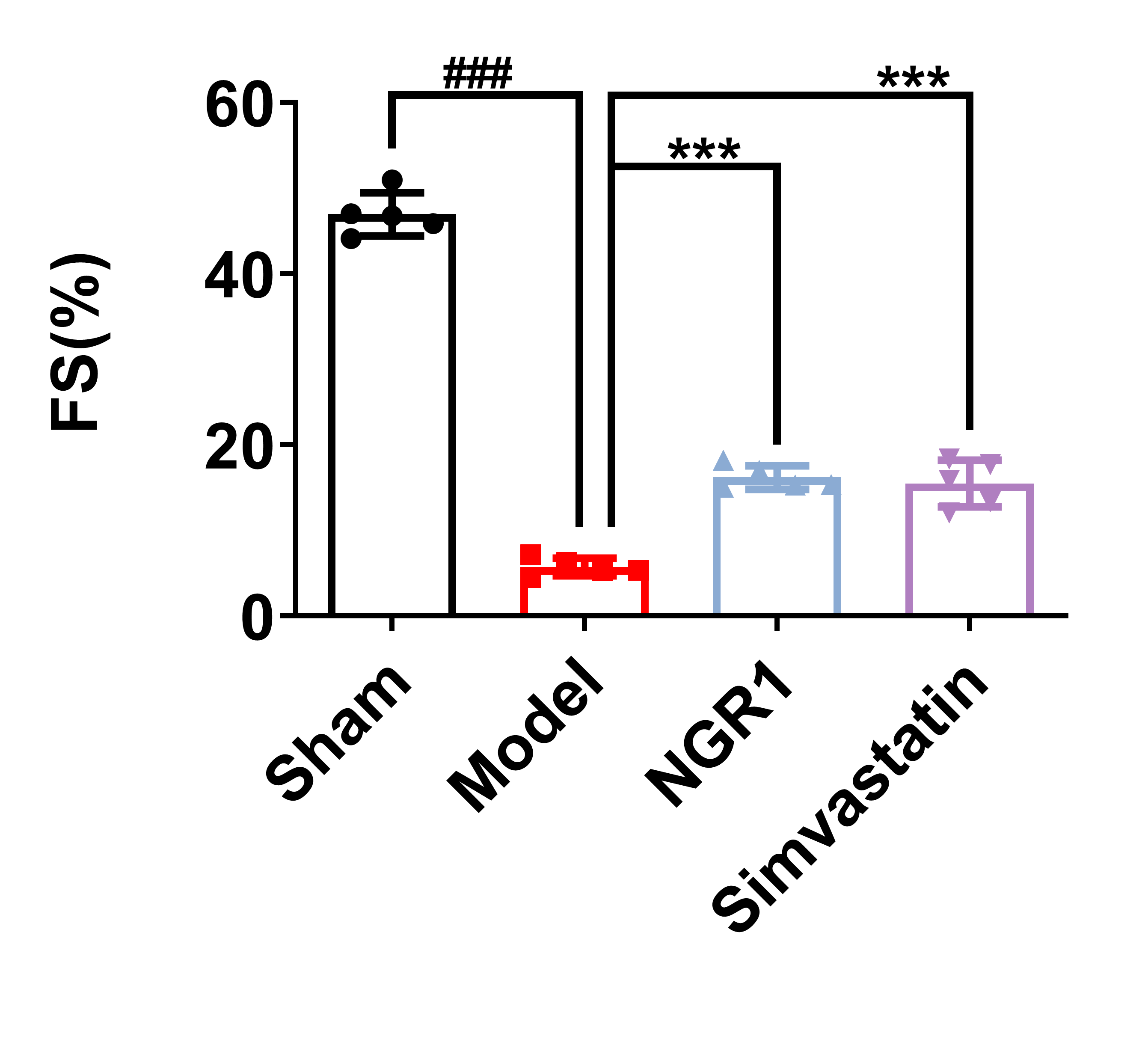

Supplement: Supplementary file 12 [file DataSheet7.ZIP › Fig 1A-B/FS.tif]

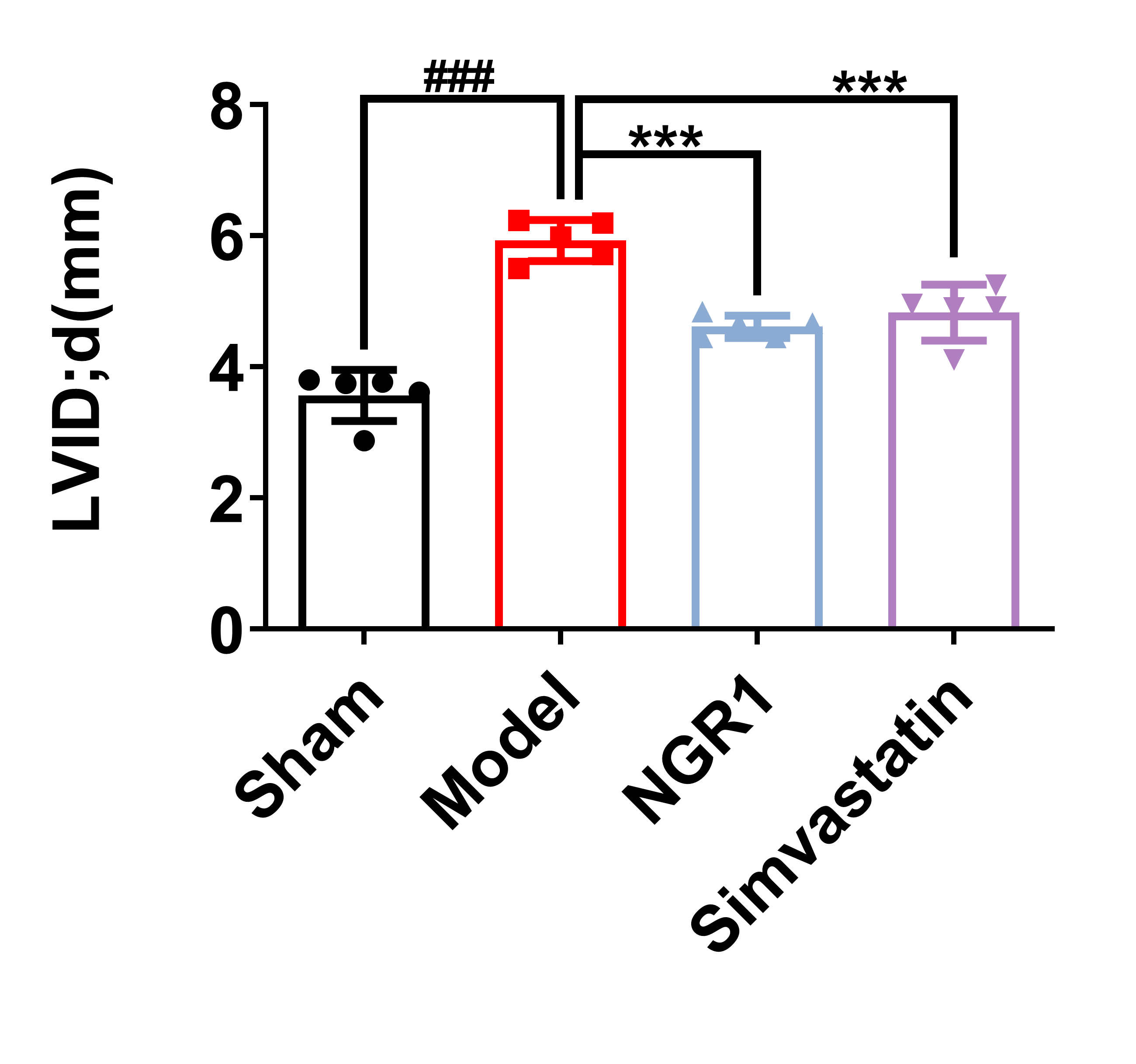

Supplement: Supplementary file 12 [file DataSheet7.ZIP › Fig 1A-B/LVIDD.tif]

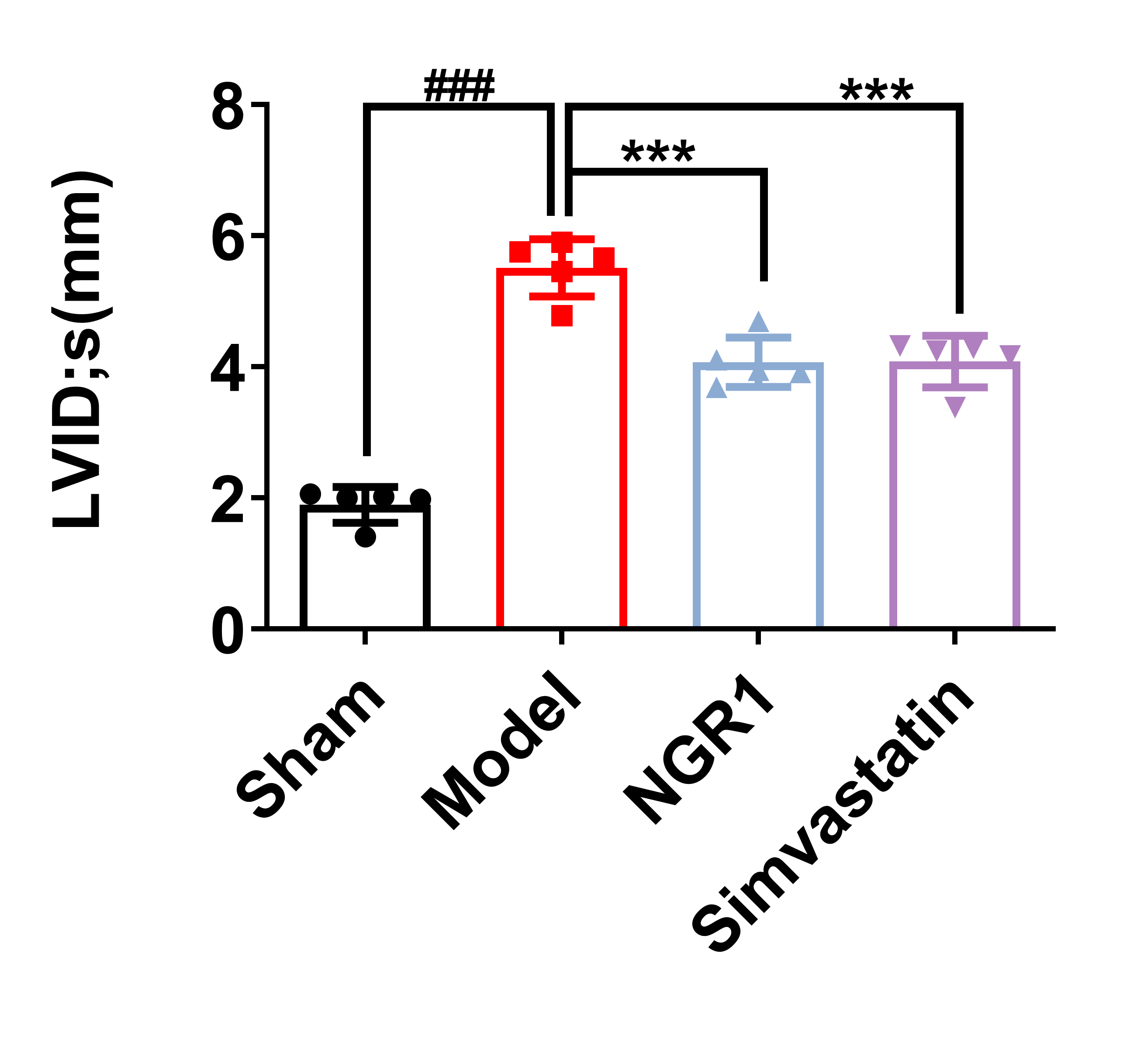

Supplement: Supplementary file 12 [file DataSheet7.ZIP › Fig 1A-B/LVIDS.tif]

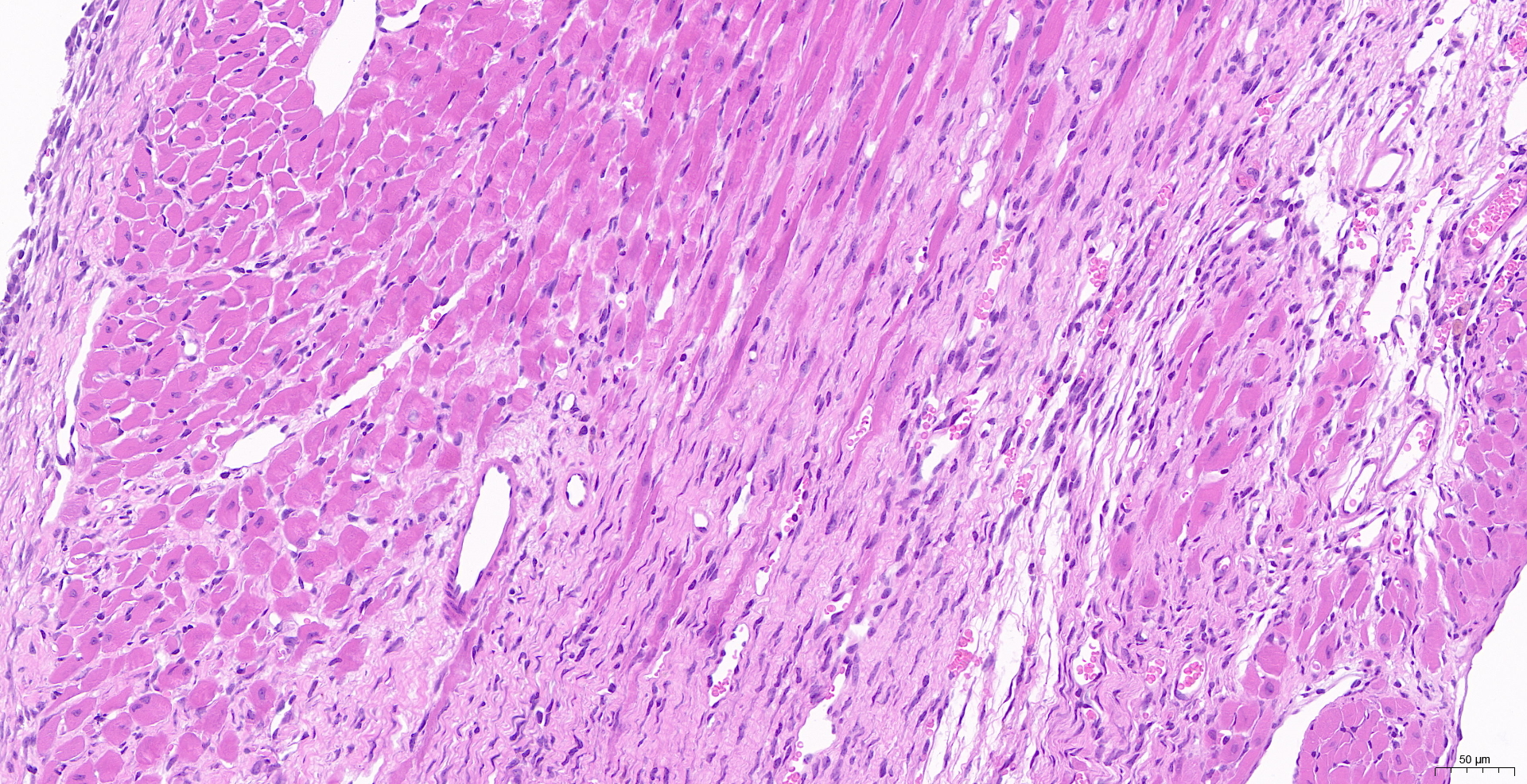

Supplement: Supplementary file 12 [file DataSheet7.ZIP › Fig 1C/20x/Fig 1C Model HE_20.0x.jpg]

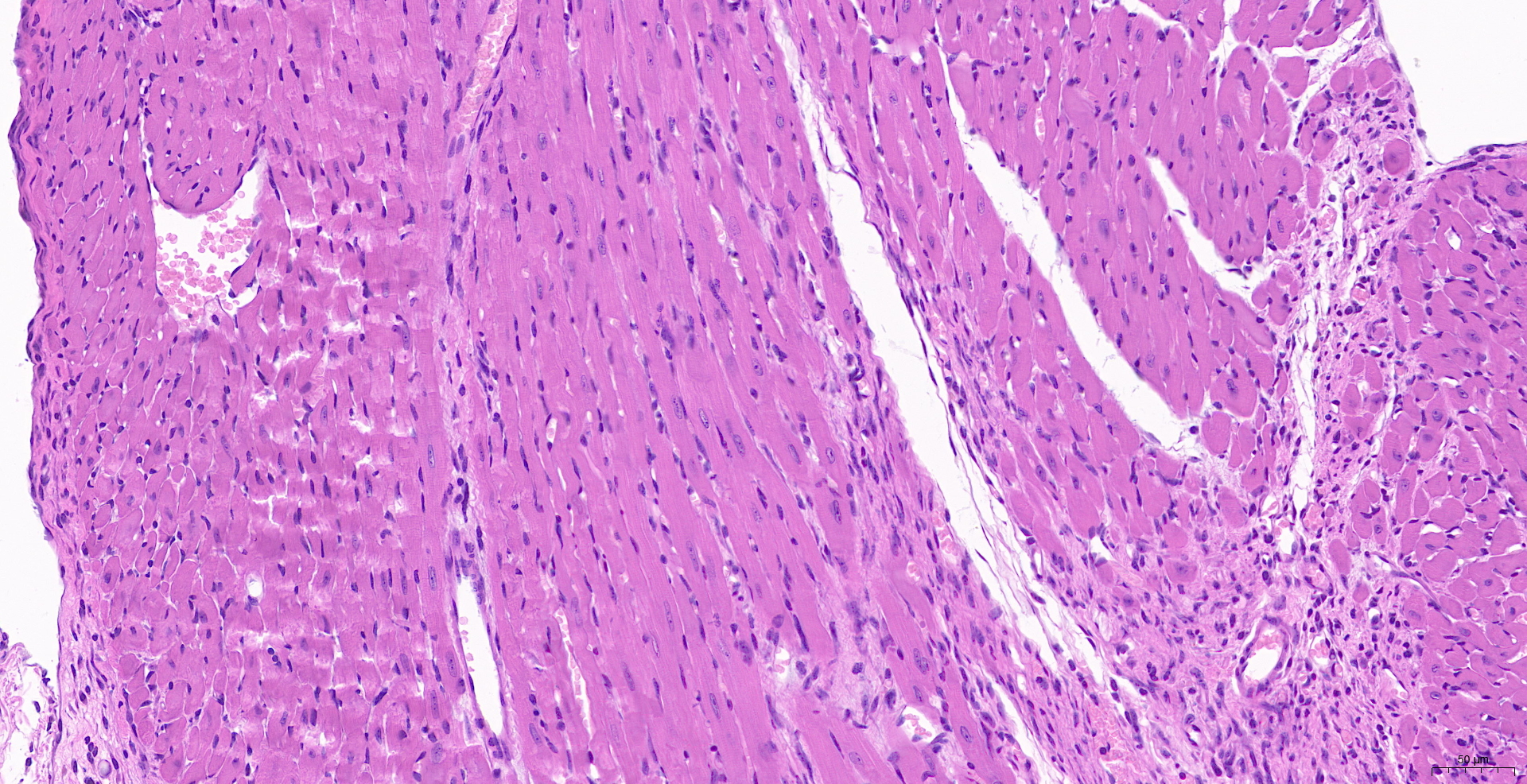

Supplement: Supplementary file 12 [file DataSheet7.ZIP › Fig 1C/20x/Fig 1C NGR1 HE_20.0x.jpg]

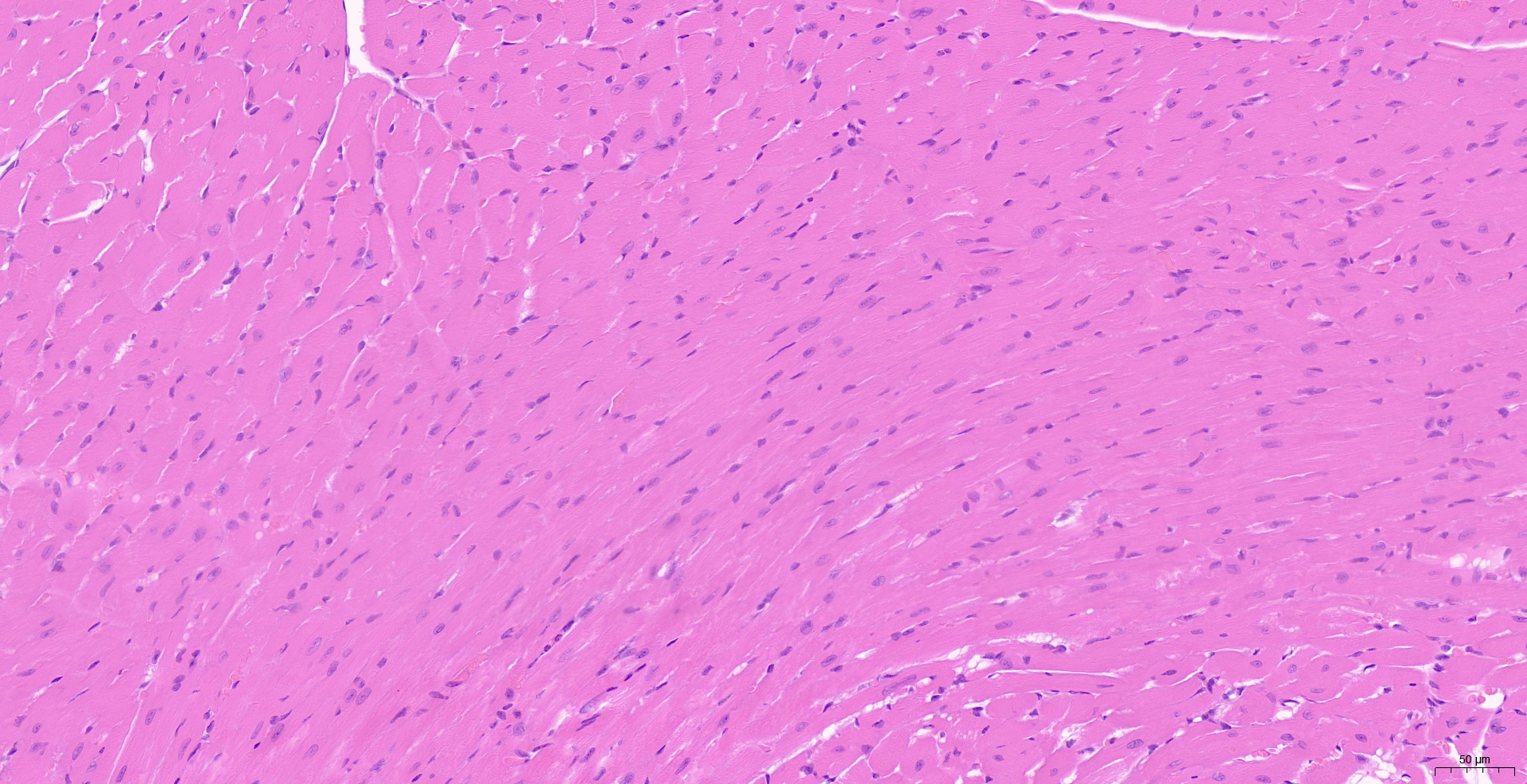

Supplement: Supplementary file 12 [file DataSheet7.ZIP › Fig 1C/20x/Fig 1C Sham HE _20.0x.jpg]

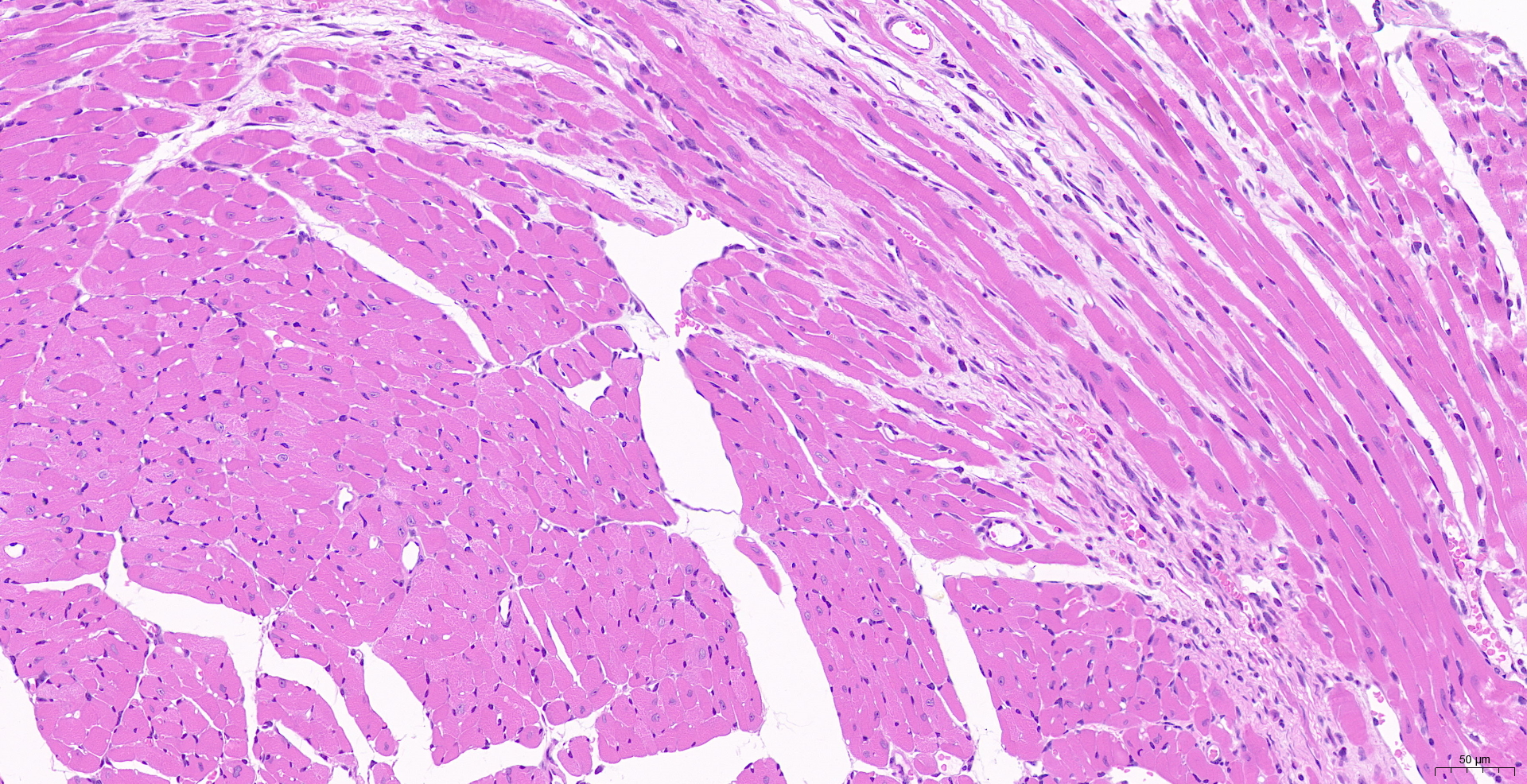

Supplement: Supplementary file 12 [file DataSheet7.ZIP › Fig 1C/20x/Fig 1C Simvastatin HE_20.0x.jpg]

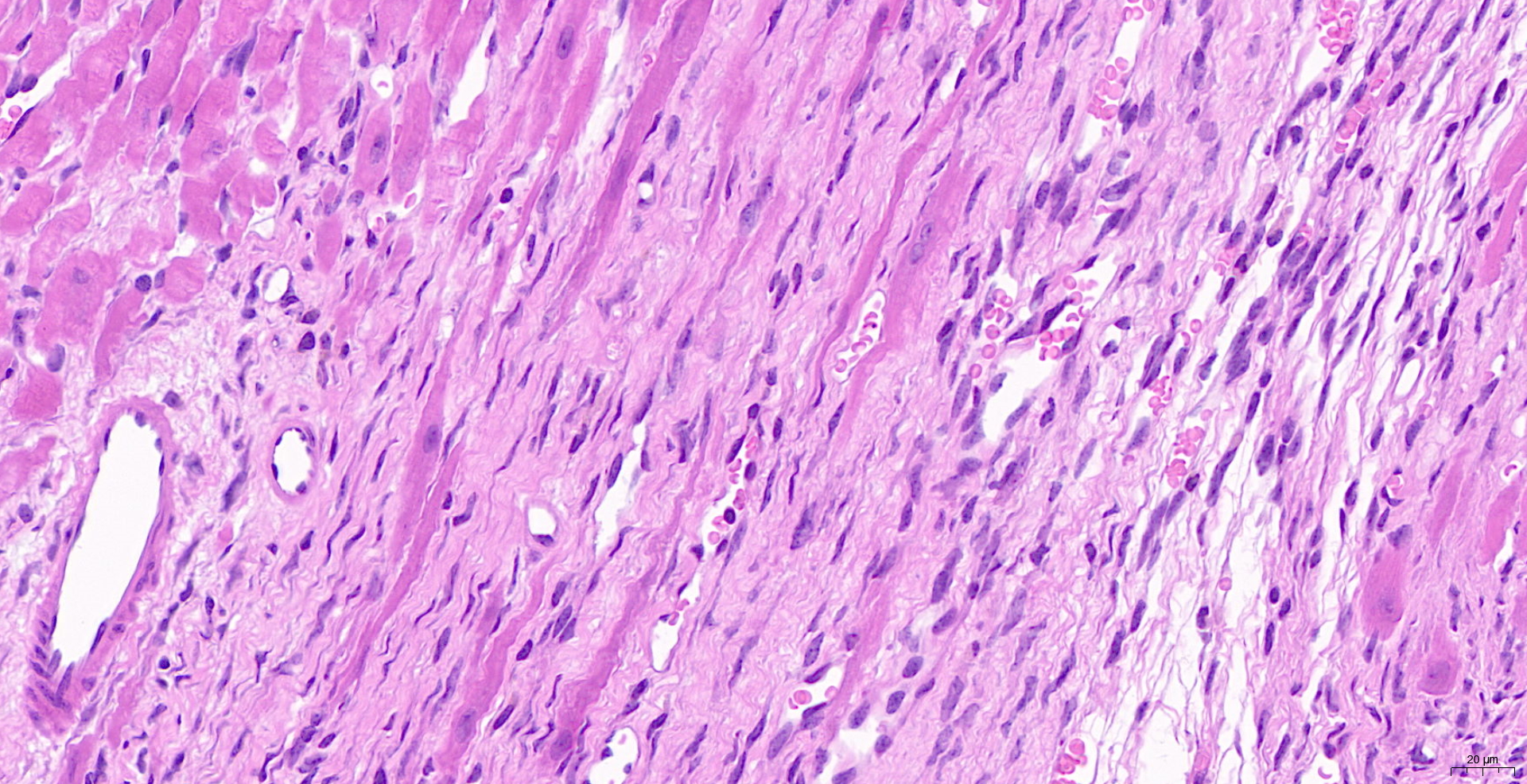

Supplement: Supplementary file 12 [file DataSheet7.ZIP › Fig 1C/40x/Fig 1C Model HE_40.0x.jpg]

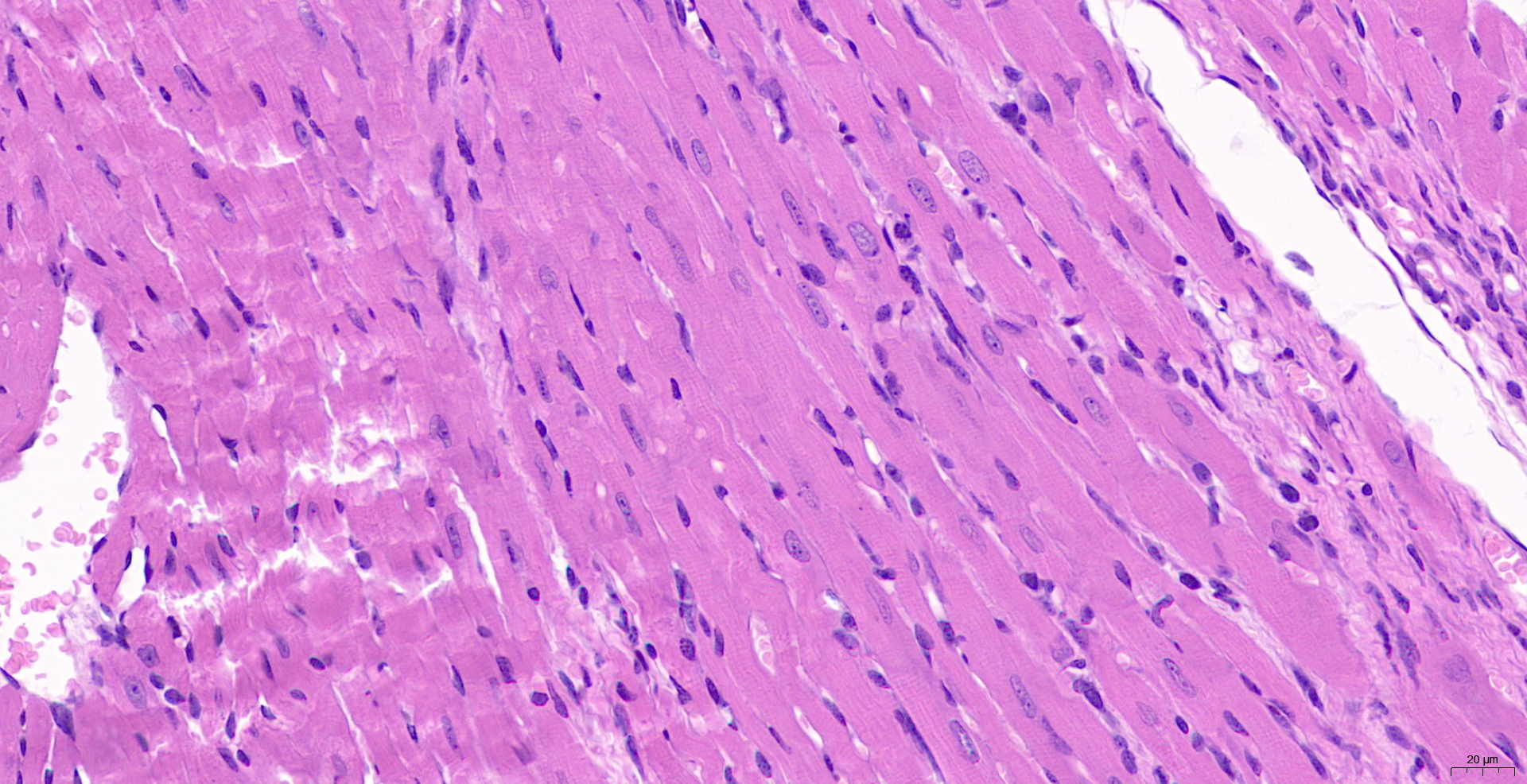

Supplement: Supplementary file 12 [file DataSheet7.ZIP › Fig 1C/40x/Fig 1C NGR1 HE_40.0x.jpg]

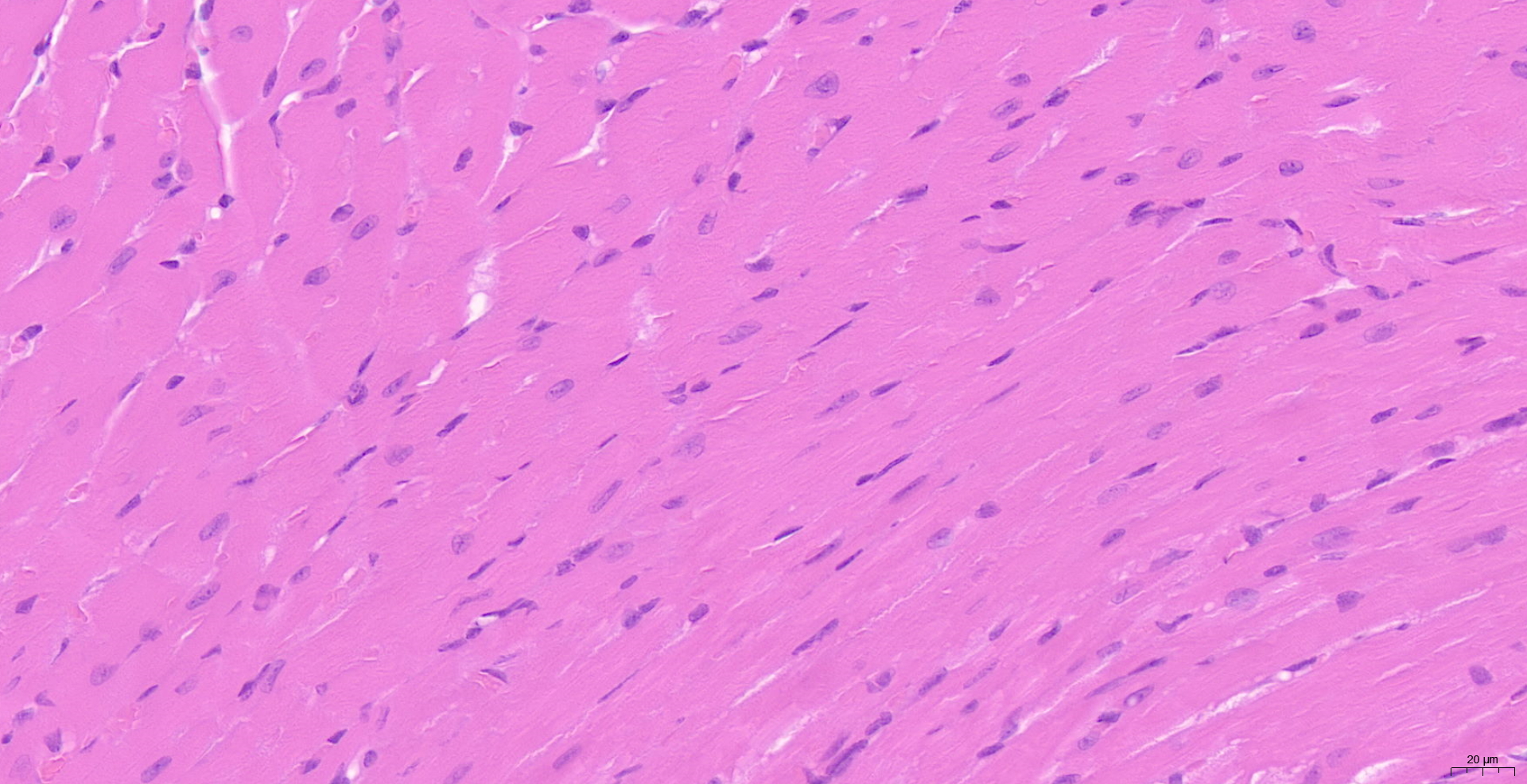

Supplement: Supplementary file 12 [file DataSheet7.ZIP › Fig 1C/40x/Fig 1C Sham HE _40.0x.jpg]

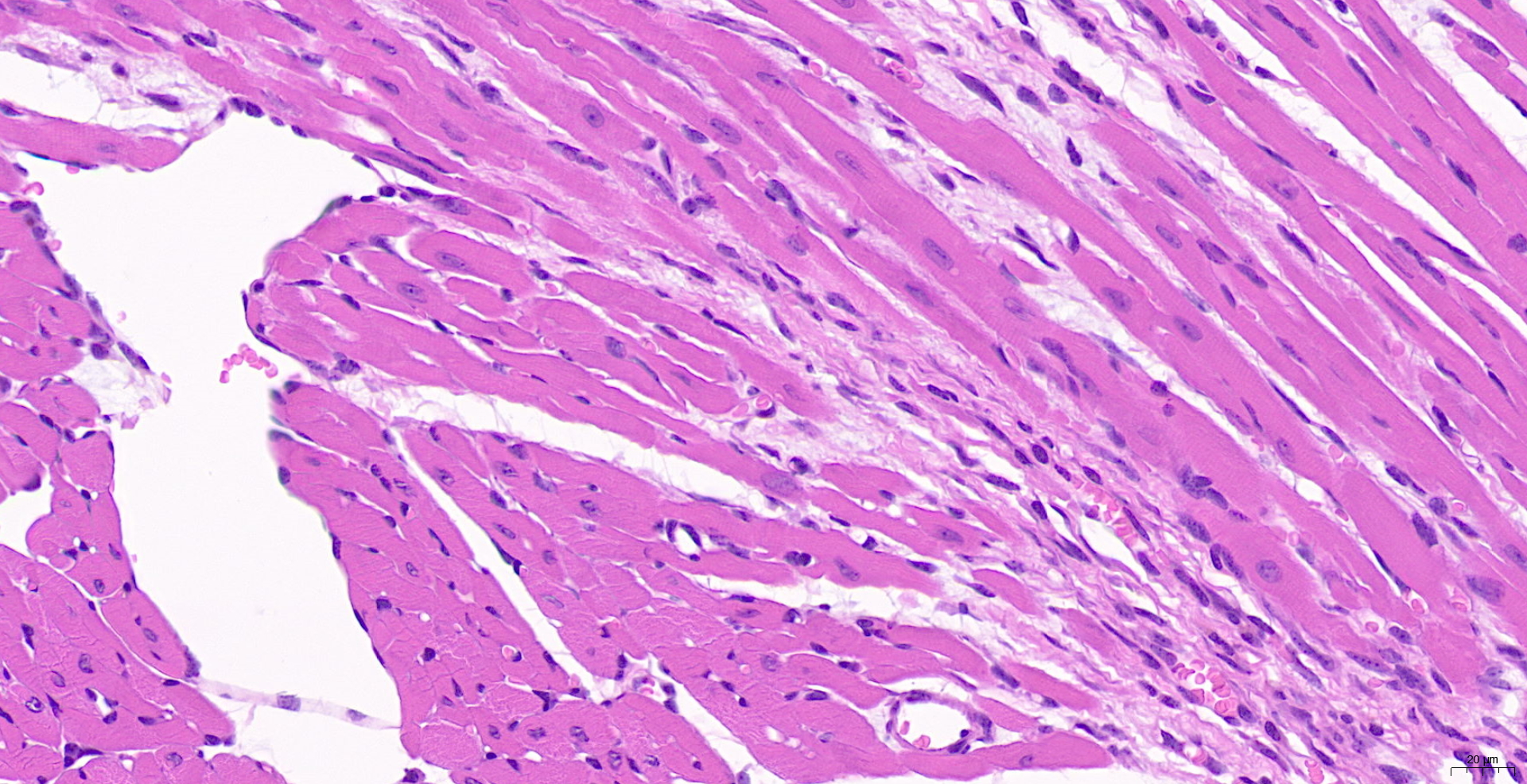

Supplement: Supplementary file 12 [file DataSheet7.ZIP › Fig 1C/40x/Fig 1C Simvastatin HE_40.0x.jpg]
